# Direct C–H functionalization of difluoroboron dipyrromethenes (BODIPYs) at β-position by iodonium salts†

**DOI:** 10.1039/c7ra13070h

**Published:** 2018-02-01

**Authors:** Wenming Ren, Huaijiang Xiang, Chengyuan Peng, Zulipali Musha, Jingjing Chen, Xin Li, Ruimin Huang, Youhong Hu

**Affiliations:** State Key Laboratory of Drug Research, Shanghai Institute of Materia Medica, Chinese Academy of Sciences 555 Zuchongzhi Road Shanghai 201203 China yhhu@simm.ac.cn rmhuang@simm.ac.cn; College of Pharmaceutical Sciences, Zhejiang University 866 Yuhangtang Road Hangzhou 310058 China; University of Chinese Academy of Sciences 19 Yuquan Road Beijing 110039 China

## Abstract

A copper-catalyzed direct C–H arylation or vinylation of BODIPYs at the β-position by iodonium salts has been developed, which provides facile access to a variety of mono-substituted BODIPY dyes. Interestingly, β-styryl BODIPY compound 9b exhibits apparent cytotoxicity after laser irradiation, which has great potential for photodynamic therapy.

## Abstract 2

A copper-catalyzed direct C–H arylation or vinylation of BODIPYs at the β-position by iodonium salts has been developed and β-styryl BODIPY 9b has great potential for photodynamic therapy.

## Graphical abstract

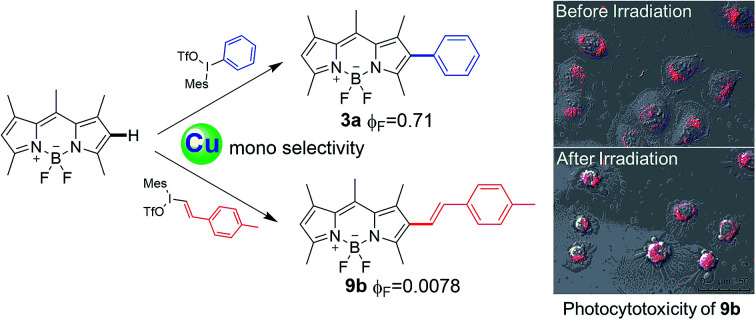

## Introduction

**Scheme 1:**
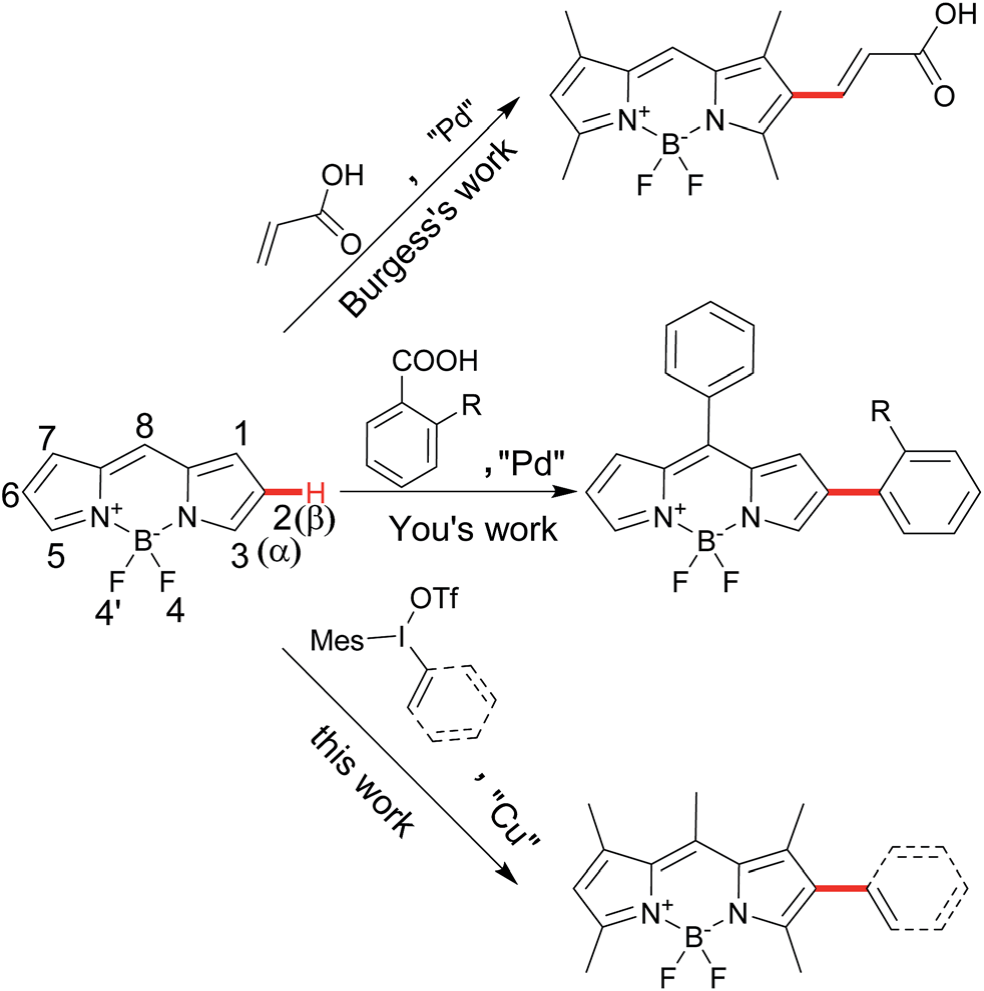
Direct arylation or vinylation of BODIPY at β-position.

Difluoroboron dipyrromethenes (BODIPYs) and their derivatives are most widely used as small molecule organic fluorophores due to its excellent features, such as high fluorescence quantum yield, narrow emission bandwidth with high peak intensity, good biocompatibility and high photophysical stability.^1^ Modification of the BODIPY framework can tune for their fluorescence profile and functionality for the specific application such as bio-imaging and photodynamic therapy (PDT).^2^ Besides the meso-derivation for chemosensors, the extension of BODIPY core by the conjugated groups at the α- or β-position of could lead the advantage of red-shifted emission band and large Stokes shift.^1*a*,3^ Introducing diverse functional groups based on the BODIPY directly is an efficient way to avoid the use of unstable intermediate and multiple-steps processes from pyrroles. Traditionally, the installation of functional conjugated groups has been proceeded by the coupling reaction from halogenated BODIPY or the condensation from active carbon (methyl group at α-position and formyl group at β-position).^3,4^ Direct C–H functionalization of BODIPY chromophores has become an attractive alternative due to the economy and efficiency.^5^ Several groups successfully developed the C–H α-arylation of BODIPYs from the different aryl sources.^6^ However, the conjugated β-derivation is rarely explored. Burgess and coworkers reported a few examples of palladium-catalyzed C–H Heck-type alkenylation at the β-position in 5 days convertion.^7^ You and coworkers reported decarboxylative C–H arylation at the β-position from specific *ortho*-substituted ben-zoic acids in the harsh conditions to obtain the mono-and disubstituted products.^8^ Herein, we report a direct C–H arylation or vinylation method for BODIPY postmodification by iodonium salts at the β-position for the mono-substitution as the major product, which could furtherly result the diversity at α- and β-positions of the BODIPYs (Scheme 1).

## Results and discussion

**Scheme 2:**
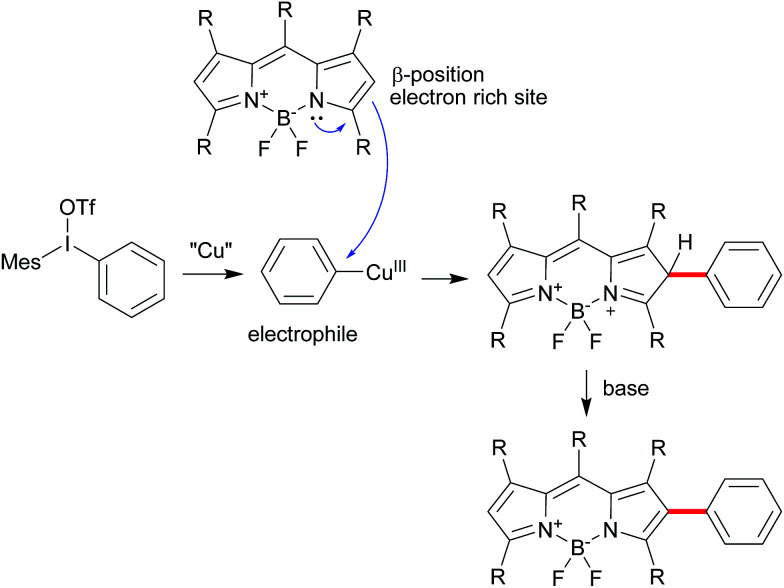
The possible reaction mechanism for β-arylation of BODIPY.

Diaryliodonium salts as highly reactive species have been widely applied to versatile arylation reaction.^9^ Direct α-selective arylation of BODIPYs by diaryliodonium salts could be smoothly proceeded through radical reaction pathway with metal free.^6*c*^ Since the β-position of the BODIPY is also potentially electron rich,^10^ we hypothesized the direct β-arylation of BODIPYs using diaryliodonium salt could be proceed by blocking the active α-position (Scheme 2). We selected the readily available pentamethyl-substituted BODIPY 1 to investigate the β-arylation reaction since the methyl group could block the active α-position and be easily functionalized further. Under the metal-free conditions reported previously,^6*c*^ the reaction of BODIPY 1 with diaryliodonium triflates 2a did not happen. Since diaryliodonium salt could behave as a highly activated aromatic electrophile by Cu catalyst,^11,12^ the reaction was carried out in the presence of copper trifluoromethanesulfonate (Cu(OTf)_2_, 0.1 equiv.) and 2,6-di-*tert*-butylpyridine (DTBP, 1.2 equiv.) as base in DCM at 70 °C for 6 hours. As expected, the desired mono-arylated product 3a was obtained in 48% yield with an excellent selectivity. Among the different solvents (Table 1, entries 1–4), the DCM is best. By the detailed analysis of the reaction, we found that de-boron difluoride byproduct was mainly formed in DCM and the starting material 1 could be easily recovered in toluene. Since the acidic conditions might cause the deboronation of the BODIPY,^13^ we tried to the mixture solvents of DCM and toluene. The co-solvents (DCM : toluene = 3 : 1) gave the best yield of 3a with high selective ratio (∼10 : 1) with the recovered starting material 1. Without base or with inorganic base, the reaction didn't give the satisfied results (Table 1, entries 8, 9). In addition, counter anions of diaryliodonium such as PF_6_ or Cl were tested (Table 2, entries 10, 11). The results uncovered the observation that electron-withdrawing counter anion PF_6_ afforded the similar yield and selectivity as OTf. By increasing the equivalents of iodonium triflate salt to 2.4, mono-arylated 3a and diarylated 4a were obtained in 21% and 50%, respectively.

**Table 1:** Optimization of reaction conditions for β-arylation of BODIPY^a^

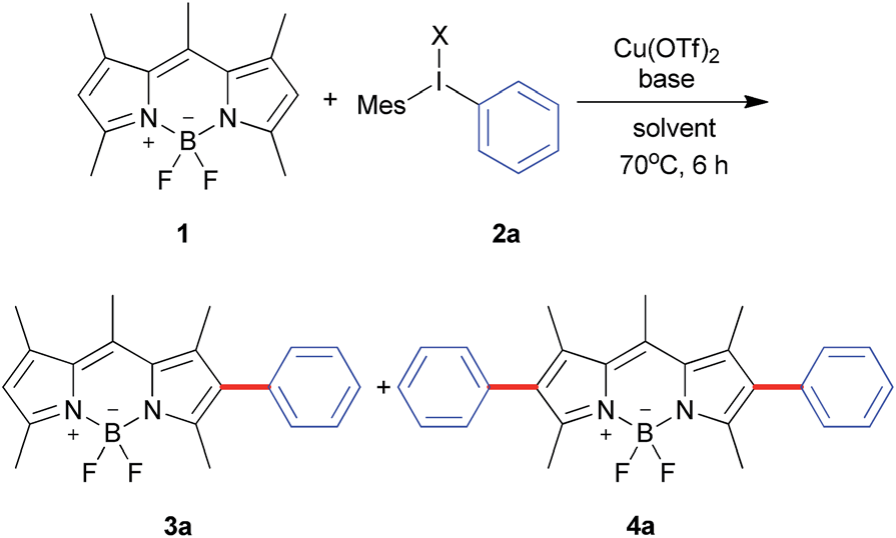					
Entry	Solvent	Base	X	Yield^b^ (%)	
				3a	4a
1	DCM	DTBP	OTf	48	6
2	DCE	DTBP	OTf	43	8
3	Toluene	DTBP	OTf	18	5
4	1,4-Dioxane	DTBP	OTf	8	—
5	DCM : toluene = 1 : 1	DTBP	OTf	38	4
**6**	**DCM : toluene = 3 : 1**	**DTBP**	**OTf**	**53**	**5**
7	DCM : toluene = 5 : 1	DTBP	OTf	39	4
8	DCM : toluene = 3 : 1	—	OTf	15	3
9	DCM : toluene = 3 : 1	NaHCO_3_	OTf	15	7
10	DCM : toluene = 3 : 1	DTBP	PF_6_	52	5
11	DCM : toluene = 3 : 1	DTBP	Cl	—	—
**12** c	**DCM : toluene = 3 : 1**	**DTBP**	**OTf**	**21**	**50**

a Reaction condition: 1 (0.24 mmol), 2a (0.20 mmol), solvent (2 mL), Cu(OTf)_2_ (0.02 mmol), base (0.24 mmol), 70 °C, 6 h, sealed tube under N_2_.

b Isolated yields.

c 1 (0.20 mmol), 2a (0.48 mmol), solvent (3 mL), Cu(OTf)_2_ (0.04 mmol), base (0.48 mmol), 70 °C, 6 h, sealed tube under N_2_.

**Table 2:** Photophysical properties of BODIPY dyes in CH_3_CN at room temperature

Dyes	*λ*_abs_ a (nm)	*ε* (M^−1^ cm^−1^)	*λ*_ex_ b (nm)	*λ*_em_ b (nm)	*Φ*_F_ a ^,^ c	Stokes shift (nm)
1	492	83 400	491	504	0.98	13
3a	503	86 500	502	529	0.71	27
3b	504	79 500	503	535	0.70	32
3c	504	35 200	503	530	0.78	27
3e	504	78 700	504	551	0.24	47
3f	501	72 000	501	523	0.86	22
3g	504	83 100	502	527	0.91	25
3h	502	81 600	502	527	0.70	25
3i	503	69 600	502	527	0.66	25
3j	502	80 300	502	530	0.77	28
3k	502	85 100	501	525	0.80	24
5	519	65 400	517	574	0.41	57
7	509	65 400	510	534	0.67	24

a Data were measured in a concentration of 3.0 × 10^−6^ M.

b Data were measured in a concentration of 5.0 × 10^−6^ M.

c All fluorescence quantum yields (*Φ*) were calculated using fluorescein in 0.1 N NaOH solution (*Φ* = 0.91, excitation = 488 nm) as the standard except 4l using Rhodamine 6G in anhydrous ethanol (*Φ* = 0.95, excitation = 530 nm) as the standard.

**Scheme 3:**
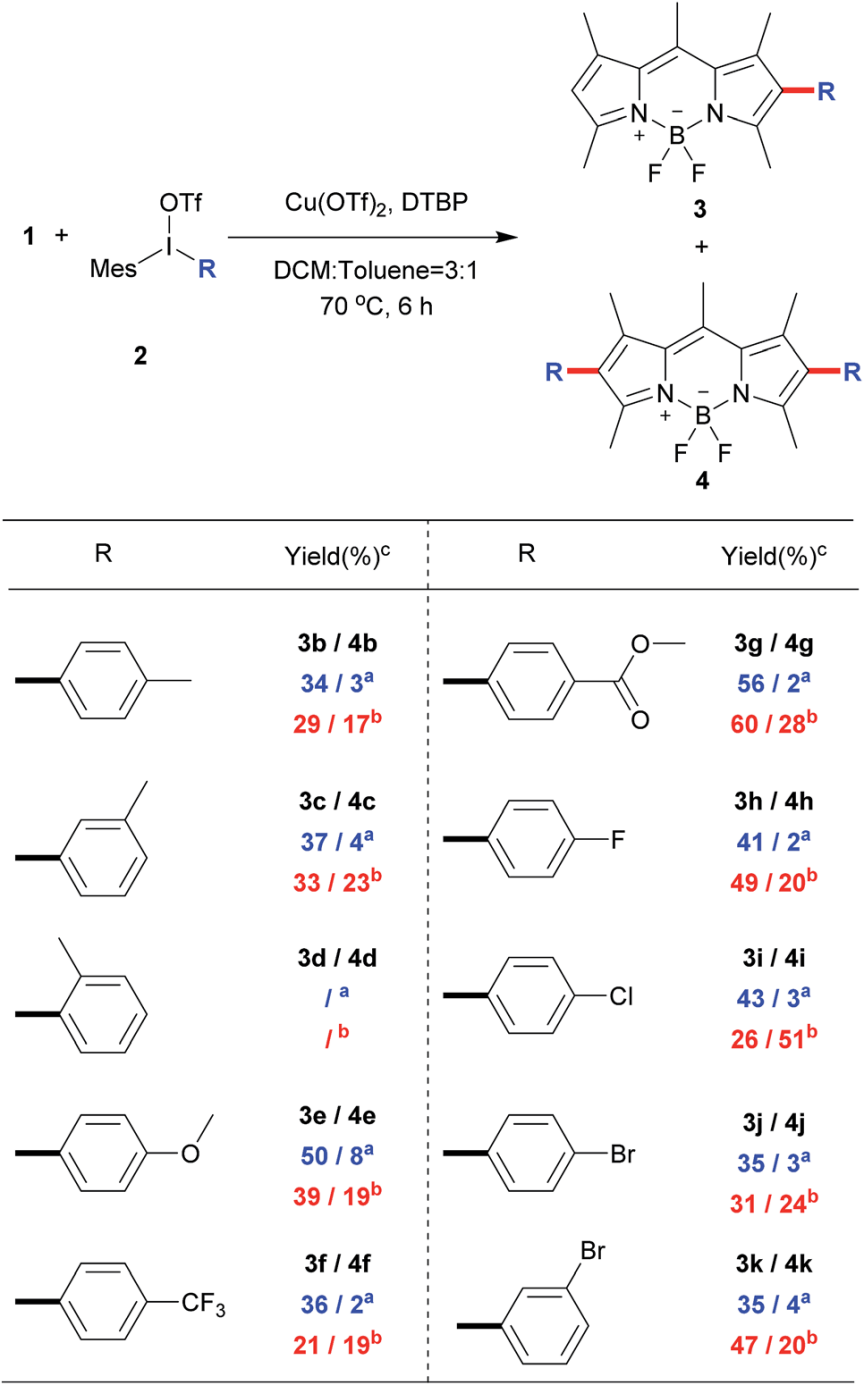
Direct C–H arylation of BODIPY 1 with different diaryliodonium triflates.^a^ Condition A: 0.8 equiv. of 2 was used; ^b^ condition B: 2.4 equiv. of 2 was used; ^c^isolated yield.

**Scheme 4:**
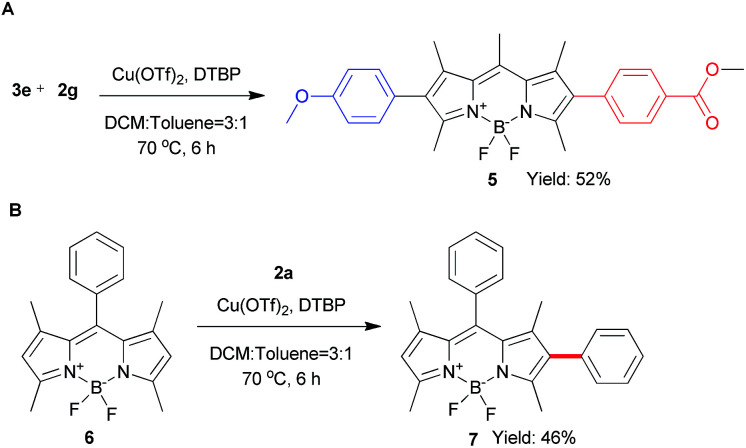
Extension for β-arylation of BODIPY.

To explore the phenyl substituent scope for this novel Cu(ii)-catalyzed C–H arylation process, various asymmetrical diaryl-iodonium triflates 2 were evaluated to react with 1 under the optimized conditions (Scheme 3). Gerenally, the results showed that both electron-withdrawing and electron-donating substituted diaryliodonium triflates 2 were suitable to this reaction, and desired products 3 were obtained in modest yields with good selectivity by using the 0.8 equiv. of 2 (condition A). Unfortunately, methyl group at *ortho*-position didn't form the desired product due to the probable steric hindrance of diaryliodonium salt (Scheme 3, compound 3d). Significantly, the bromo substituted diaryliodonium triflates performed well under this reaction conditions, producing compounds 3j and 3k, which could be further modified by coupling reactions. Increasing the amount of diaryliodonium triflates 2 to 2.4 equivalent (condition B), the overall yields of 3 and 4 could be improved with less selectivity. Since the resultant mono-substituted BODIPYs could be arylated by the different diaryliodonium triflates to generate the diversity, we applied compound 3e (Scheme 4A) as the substrate with *para*-methoxycarbonyl group as electron-donor to react with substituted diaryliodonium triflate 2g with electron-acceptor. As expected, the asymmetrical diarylated BODIPY 5 containing D–π–A (donor–π–acceptor) structure was obtained in 52% yield. In addition, *meso*-phenyl BODIPY 6 could react with diaryliodonium 2a (Scheme 4B) under the condition A to achieve product 7 in 46% yield.

The photophysical properties of 3a–3k, 5 and 7 were evaluated in different solvents system (Table 2, Fig. S1–5†). As shown in Table 2, 2-aryl BODIPYs 3a–3k exhibited the absorption maxima and excitation maxima among 501–504 nm no matter electron-withdrawing or electron-donating substituents. All 2-aryl BODIPYs showed high absorption coefficients and relatively high fluorescence quantum yields. Methoxyl substituent 3e showed a lower fluorescence quantum yield (∼24%) correspondingly. On the other hand, 3e exhibited the longest emission maxima at 551 nm and the largest stokes shift (47 nm) among 2-aryl BODIPYs. These data indicate that slight modification of BODIPY could change their photophysical property significantly. The asymmetrical diarylated BODIPY 5 containing D–π–A has the desired properties with red fluorescence, high quantum yield and large Stokes shift.

**Scheme 5:**
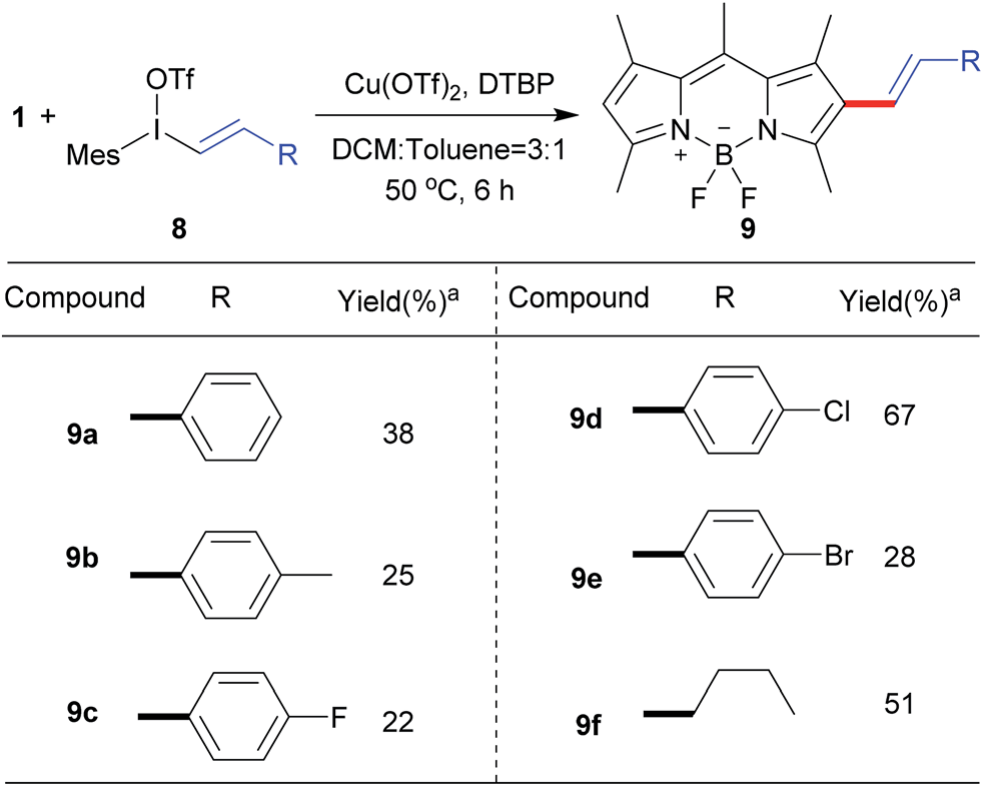
Scope of the direct C–H vinylation of BODIPY 1 with different vinyliodonium triflates.^a^Isolated yield.

Next, we explored the reaction of BODIPY 1 with the variety of styryliodonium triflates 8 to generate the different conjugated systems. Due to the higher reactivity of vinyliodonium than aryliodonium salts,^12*b*^ the reaction temperature at 50 °C gave the similar efficiency at 70 °C. To our delight, the different 2-styryl BODIPYs 9a–9e were delivered (Scheme 5). Additionally, alkyl vinyliodonium triflates can also be successfully applied in this reaction, maintaining 51% yield of 2-vinyl BODIPY 9f.

**Fig. 1:**
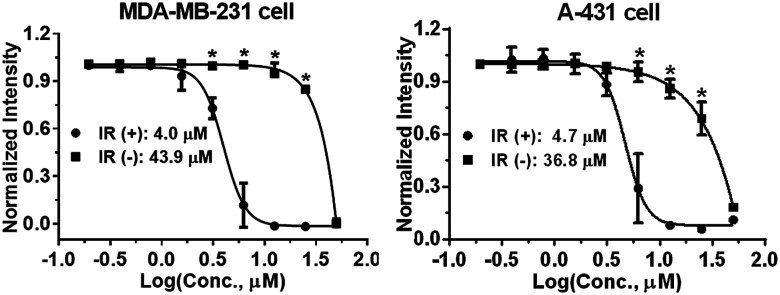
Light irradiation (IR)-induced cytotoxicity by compound 9b in MDA-MB-231 and A-431 cells using SRB assay. *P < 0.0001.

**Fig. 2:**
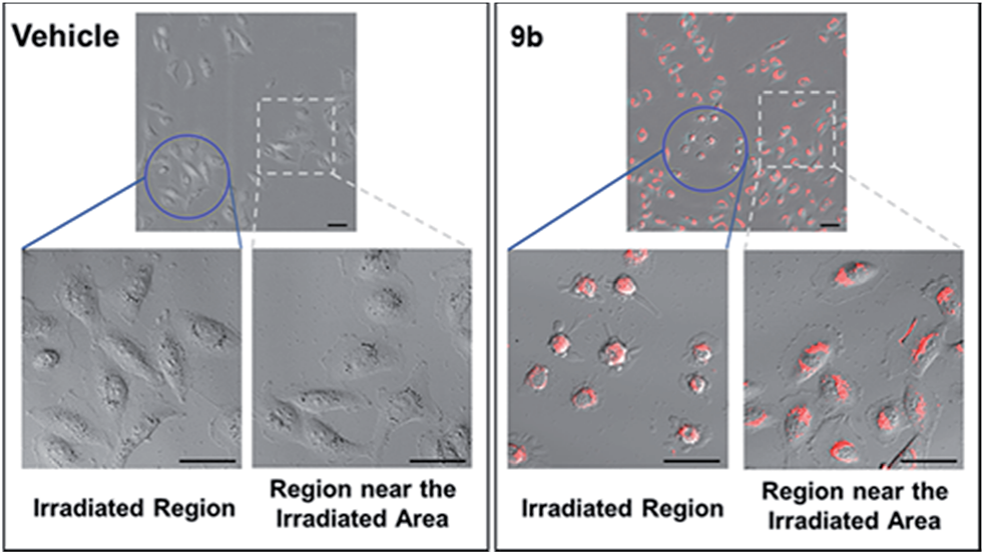
Overlay of fluorescent and differential interference contrast (DIC) images of MDA-MB-231 cells incubated with of compound 9b or vehicle control. Blue circles, laser irradiated regions; grey boxes, regions very near the laser-irradiated areas. Scale bars, 50 μm.

Photophysical properties of 9a–9f were investigated as shown in Table 3. The 2-vinyl BODIPYs 9 exhibited emission maxima up to 598–620 nm with large stokes shifts. Probably due to the geometry relaxation of fluorophores upon photoexcitation, the quantum yields of those dyes were as low as 0.78%. Dye with photoproperties such as high extinction coefficient, low quantum yield and red emission could be used as photosensitizer for photodynamic therapy.^2*a*,2*d*^ The BODIPY 9b was thus chosen to evaluate photocytotoxic activity *in vitro*. The half maximal inhibitory concentration (IC_50_) of compound 9b with or without light irradiation was assessed in human breast cancer cell line MDA-MB-231 and epidermoid carcinoma cell line A-431. Nine different concentrations (ranging from 0.195 to 50 μM) of 9b were applied to cells and 12 h exposure to mercury lamp decreased the cell viability significantly in presence of 9b (*P* < 0.0001) in both cells (Fig. 1). For example, light irradiation reduced the IC_50_ value from 43.9 μM to 4.0 μM (∼88% decline) in MDA-MB-231 cells. Photophysical property and laser-induced cytotoxicity of compound 9b were also characterized and visualized by a Leica confocal microscopy. As shown in Fig. 2 and S7,† high fluorescence signals were detected in cytoplasm within 5 min after 9b (2.5 μM) feeding. Then the photothermal treatment was performed by continuous irradiation of the cells using a 552 nm laser for 10 min, leading to a ∼200 μm diameter irradiation spot. Cell blebbing, a characteristic feature of injured cells, was only observed in compound 9b-treated cells but not in the vehicle-treated cells. Similar photothermal effects were exhibited in 9a- or 9f-treated cells (Fig. S7†). These results suggest that 2-vinyl BODIPYs could be excellent photosensitizers and have potential for photodynamic therapy of cancer.

**Table 3:** Photophysical properties of BODIPY dyes in CH_3_CN at room temperature

Dyes	*λ*_abs_ a (nm)	*ε* (M^−1^ cm^−1^)	*λ*_ex_ b (nm)	*λ*_em_ b (nm)	*Φ*_F_ a ^,^ c	Stokes shift (nm)
9a	523	50 100	517	608	0.0080	91
9b	520	38 100	519	620	0.0078	101
9c	522	52 400	505	600	0.0091	95
9d	520	70 600	518	610	0.011	92
9e	523	47 700	521	607	0.010	86
9f	511	43 100	535	598	0.063	63

a Data were measured in a concentration of 3.0 × 10^−6^ M.

b Data were measured in a concentration of 5.0 × 10^−6^ M.

c The fluorescence quantum yields (*Φ*) were calculated using rhodamine 6G in anhydrous ethanol (*Φ* = 0.95, excitation = 530 nm).

## Conclusions

In conclusion, we have developed the Cu-catalyzed direct C–H arylation and vinylation of BODIPYs at the β-position. This methodology provides a convenient synthetic procedure to prepare diversified BODIPY derivatives. These reactions exhibited good selectivity for mono-substituted product to allow the different modification of BODIPY at 2,6-position. Through the evaluation of photophysical properties of those dyes, 2-styryl BODIPYs showed intrinsic large Stokes shift up to 101 nm and low quantum yield, suggesting the potential for photodynamic therapy of cancer. In this way, the novel functionality of fluorophores with asymmetrical substitution can be synthesized economically and efficiently.

## Experimental

### General

All reactions were carried out under an atmosphere of argon in oven-dried glassware with magnetic stirring. All commercially available reagents were used as received. Chromatographic purifications were performed by flash chromatography with silica gel (40–63 μm) packed in glass columns. The eluting solvent for the purification of each compound was determined by thin-layer chromatography (TLC) on glass plates coated with silica gel 60 F254 and visualized by UV light (254 nm or 365 nM). ^1^H NMR spectra and proton-decoupled ^13^C NMR spectra were obtained on a 400 MHz or 500 MHz Bruker NMR spectrometer. ^1^H chemical shifts (*δ*) are reported in parts per million (ppm) relative to TMS (s, *δ* 0). ^13^C NMR chemical shifts are reported relative to CDCl_3_ (t, *δ* 77.4). High-resolution mass data were obtained on an Agilent 6224 accurate-mass TOF LC/MS (ESI). Absorption spectra were acquired using a Varian Cary 300 spectrophotometer. Fluorescence measurements were carried out on a Horiba FluoroMax-4 spectrometer. Quantum yields were determined in reference to either fluorescein or rhodamine 6G and corrected for solvent refractive index. The extinction coefficients were determined through Beer's law plots. All data were measured at room temperature. Human breast cancer cell line MDA-MB-231 and human epidermoid carcinoma cell line A-431 were obtained from the American Type Culture Collection (Manassas, VA, USA) and were cultured in DMEM (high glucose) and RPMI 1640 medium respectively, supplemented with 10% fetal bovine serum (Hyclone, Logan, UT). Cells were incubated at 37 °C in 5% CO_2_ in air.

### General procedure for the Cu(ii)-catalyzed mono-arylation or vinylation of BODIPYs (reaction condition A)

Copper(ii) trifluoromethanesulfonate (7.23 mg, 0.02 mmol) was adequately suspended in 2 mL mixture solution (dichloromethane/toluene = 3/1, dried by 4 Å molecular sieves) in 10 mL sealed tube, then 1 (62.91 mg, 0.24 mmol), appropriate diaryliodonium salt (0.2 mmol) and 2,6-di-*tert*-butylpyridine (54 μL, 0.24 mmol) were sequentially added to the solution. Seal up the tube with argon atmosphere. The resulting solution was stirred at 50 °C (R = styryl) or 70 °C (R = aryl group) for 6 h. The solution was concentrated and purified by silica gel column chromatography (hexane: dichloromethane: acetic ether = 300 : 100 : 0 to 300 : 100 : 5) to afford crude product. Single and double substituted products were validated by 400 MHz or 500 MHz Bruker NMR spectrometer. The residue was purified by TLC silica gel plate (10 × 200 × 200 mm, 10–40 μm, separating solvent and ratio: dichloromethane/toluene = 1/5) or reversed phase chromatography (separating solvent and ratio: H_2_O/MeOH = 15/85 to 5/95) to afford pure products 3a–k, 5, 7 and 9a–g. The yield data was calculated by diaryliodonium stoichiometric equivalence.

### General procedure for the Cu(ii)-catalyzed diarylation of BODIPYs (reaction condition B)

Copper(ii) trifluoromethanesulfonate (14.5 mg, 0.04 mmol) was adequately suspended in 3 mL mixture solution in 10 mL sealed tube, then 1 (52.4 mg, 0.20 mmol), appropriate diaryliodonium salt (0.48 mmol) and 2,6-di-*tert*-butylpyridine (109 μL, 0.48 mmol) were sequentially added to the solution. Seal up the tube with argon atmosphere. The purification procedures were the same with reaction condition A to afford 4a–4k. The yield data was calculated by BODIPY 1 stoichiometric equivalence.

### Cell viability assay for photocytotoxic effect

Cells were seeded in 96-well plates at a density of 1600–3000 cells per well in triplicate. Twenty-four hours later, the cells were incubated with fresh medium with compounds at different concentrations (ranging from 0.195 to 50 μM). The cells were exposed under the mercury lamp (HXP R120W/45C; Osram, Germany) for 12 h, and then were kept under normal culture condition for another 72 h. At the endpoint, the cells were fixed with 10% pre-cooled trichloroacetic acid (Sigma, St Louis, MO, USA) over 4 h followed by staining with 4 mg mL^−1^ sulforhodamine B (SRB; Sigma) in 1% acetic acid for 15 min. SRB in the cells was dissolved in 10 mmol L^−1^, Tris–HCl and measured at 560 nm using a microplate reader (SpectraMAX190; Molecular Devices, Sunnyvale, CA, USA). The half maximal inhibitory concentration (IC_50_) was decided using (log(inhibitor) *vs.* response – variable slope (four parameters)) method in the GraphPad Prism Software. The data were presented as the mean ± SD. Differences were considered statistically significant at *P* < 0.0001 by 2 way ANOVA test.

### Visualization of laser-induced cytotoxicity

Cells were grown on the 8-well chambered coverglass (Thermo Fisher Scientific, Waltham, MA, USA) for 36 h. Immediately after adding the compound with indicated concentrations to cells, a small region of interest (ROI) was randomly chosen and irradiated using the laser line at 552 nm from a confocal microscopy (Leica TCS SP8 STED, Germany) over 10 min. During that period, the fluorescent and differential interference contrast (DIC) images were acquired at 0 min, 5 min and 10 min, respectively. The power of the laser was kept at 80% output to ensure the consistent irradiation between experiments.

#### 5,5-Difluoro-1,3,7,9,10-pentamethyl-2-phenyl-5*H*-5^λ^,6^λ^-dipyrrolo[1,2-*c*:2′,1′-*f*][1,3,2]diazaborinine(3a)

An orange solid (yield: 35.9 mg, 53%, single substitute: double substitute = 91 : 9), ^1^H NMR (400 MHz, CDCl_3_) *δ* 7.43 (t, *J* = 7.5 Hz, 2H), 7.34 (t, *J* = 7.5 Hz, 1H), 7.21 (d, *J* = 6.9 Hz, 2H), 6.08 (s, 1H), 2.65 (s, 3H), 2.54 (s, 3H), 2.47 (s, 3H), 2.44 (s, 3H), 2.32 (s, 3H). ^13^C NMR (126 MHz, CDCl_3_) *δ* 153.8, 152.2, 141.6, 141.1, 137.0, 133.9, 133.5, 132.4, 131.9, 130.4, 128.4, 127.1, 121.4, 17.5, 16.8, 15.4, 14.5, 13.2. HRMS (ESI): *m*/*z* [M + H]^+^ calcd for C_20_H_22_^11^BF_2_N_2_^+^: 339.1839; found: 339.1841, *Δ* = −0.66 ppm.

#### 5,5-Difluoro-1,3,7,9,10-pentamethyl-2-(*p*-tolyl)-5*H*-5^λ^,6^λ^-dipyrrolo[1,2-*c*:2′,1′-*f*][1,3,2]diazaborinin (3b)

An orange solid (yield: 25.9 mg, 37%, single substitute: double substitute = 93 : 7), ^1^H NMR (400 MHz, CDCl_3_) *δ* 7.24 (d, *J* = 7.7 Hz, 2H), 7.10 (d, *J* = 7.7 Hz, 2H), 6.07 (s, 1H), 2.64 (s, 3H), 2.54 (s, 3H), 2.47 (s, 3H), 2.43 (s, 3H), 2.41 (s, 3H), 2.32 (s, 3H). ^13^C NMR (126 MHz, CDCl_3_) *δ* 153.5, 152.4, 141.5, 140.8, 137.2, 136.8, 133.5, 132.3, 131.9, 130.8, 130.3, 129.1, 121.3, 21.3, 17.5, 16.8, 15.4, 14.5, 13.3. HRMS (ESI): *m*/*z* [M + H]^+^ calcd for C_21_H_24_^11^BF_2_N_2_^+^: 353.1995; found: 353.1998, *Δ* = −0.78 ppm.

#### 5,5-Difluoro-1,3,7,9,10-pentamethyl-2-(*m*-tolyl)-5*H*-5^λ^,6^λ^-dipyrrolo[1,2-*c*:2′,1′-*f*][1,3,2]diazaborinine (3c)

An orange solid (yield: 24.2 mg, 34%, single substitute: double substitute = 91 : 9), ^1^H NMR (400 MHz, CDCl_3_) *δ* 7.31 (t, *J* = 7.5 Hz, 1H), 7.16 (d, *J* = 7.6 Hz, 1H), 7.00 (d, *J* = 11.3 Hz, 2H), 6.08 (s, 1H), 2.65 (s, 3H), 2.54 (s, 3H), 2.47 (s, 3H), 2.44 (s, 3H), 2.40 (s, 3H), 2.32 (s, 3H). ^13^C NMR (126 MHz, CDCl_3_) *δ* 153.6, 152.4, 141.5, 140.8, 138.0, 137.1, 133.8, 132.3, 131.9, 131.0, 128.2, 127.9, 127.5, 121.3, 21.5, 17.4, 16.8, 15.4, 14.5, 13.2. HRMS (ESI): *m*/*z* [M + H]^+^ calcd for C_21_H_24_^11^BF_2_N_2_^+^: 353.1995; found: 353.1992, *Δ* = 0.92 ppm.

#### 5,5-Difluoro-2-(4-methoxyphenyl)-1,3,7,9,10-pentamethyl-5_H_-5^λ^,6^λ^-dipyrrolo [1,2-*c*:2′,1′-*f*] [1,3,2] diazaborinine (3e)

An orange solid (yield: 36.9 mg, 50%, single substitute: double substitute = 86 : 14), ^1^H NMR (400 MHz, CDCl_3_) *δ* 7.16–7.10 (m, 2H), 6.99–6.95 (m, 2H), 6.07 (s, 1H), 3.86 (s, 3H), 2.64 (s, 3H), 2.54 (s, 3H), 2.47 (s, 3H), 2.43 (s, 3H), 2.31 (s, 3H). ^13^C NMR (126 MHz, CDCl_3_) *δ* 158.7, 153.5, 152.6, 141.46, 140.8, 137.2, 133.3, 132.3, 131.9, 131.5, 126.0, 121.2, 113.9, 55.3, 17.4, 16.8, 15.4, 14.5, 13.2. HRMS (ESI): *m*/*z* [M + H]^+^ calcd for [M + H]^+^ calcd for C_21_H_24_^11^BF_2_N_2_O^+^: 369.1944; found: 369.1941, *Δ* = 0.95 ppm.

#### 5,5-Difluoro-1,3,7,9,10-pentamethyl-2-(4-(trifluoromethyl)phenyl)-5*H*-5^λ^,6^λ^-dipyrrolo [1,2-*c*:2′,1′-*f*] [1,3,2] diazaborinine (3f)

An orange solid (yield: 29.5 mg, 36%, single substitute: double substitute = 96 : 4), ^1^H NMR (400 MHz, CDCl_3_) *δ* 7.69 (d, *J* = 8.0 Hz, 2H), 7.33 (d, *J* = 8.0 Hz, 2H), 6.12 (s, 1H), 2.66 (s, 3H), 2.55 (s, 3H), 2.47 (s, 3H), 2.45 (s, 3H), 2.32 (s, 3H). ^13^C NMR (126 MHz, CDCl_3_) *δ* 155.1, 150.8, 142.0, 141.9, 137.9, 136.5, 132.8, 131.6, 130.7, 129.1, 128.7, 125.3, 123.1, 122.0, 17.5, 16.8, 15.3, 14.5, 13.1. HRMS (ESI): *m*/*z* [M + H]^+^ calcd for C_21_H_21_^11^BF_5_N_2_^+^: 407.1712; found: 407.1720, *Δ* = −1.97 ppm.

#### Methyl4-(5,5-difluoro-1,3,7,9,10-pentamethyl-5*H*-5^λ^,6^λ^-dipyrrolo[1,2-*c*:2′,1′-*f*][1,3,2]diazaborinin-2-yl)benzoate (3g)

An orange solid (yield: 44.4 mg, 56%, single substitute: double substitute = 96 : 4), ^1^H NMR (400 MHz, CDCl_3_) *δ* 8.10 (d, *J* = 8.1 Hz, 2H), 7.29 (d, *J* = 8.1 Hz, 2H), 6.11 (s, 1H), 3.95 (s, 3H), 2.65 (s, 3H), 2.55 (s, 3H), 2.48 (s, 3H), 2.45 (s, 3H), 2.33 (s, 3H). ^13^C NMR (126 MHz, CDCl_3_) *δ* 167.0, 154.9, 151.0, 141.9, 139.0, 136.6, 132.8, 132.1, 131.8, 130.4, 129.6, 128.8, 121.9, 52.2, 17.5, 16.9, 15.3, 14.5, 13.2. HRMS (ESI): *m*/*z* [M + H]^+^ calcd for C_22_H_24_^11^BF_2_N_2_O_2_^+^: 397.1893; found: 397.1882, *Δ* = 2.91 ppm.

#### 5,5-Difluoro-2-(4-fluorophenyl)-1,3,7,9,10-pentamethyl-5*H*-5^λ^,6^λ^-dipyrrolo[1,2-*c*:2′,1′-*f*] [1,3,2] diazaborinine (3h)

An orange solid (yield: 29.3 mg, 41%, single substitute: double substitute = 95 : 5), ^1^H NMR (400 MHz, CDCl_3_) *δ* 7.19–7.09 (m, 4H), 6.09 (s, 1H), 2.64 (s, 3H), 2.54 (s, 3H), 2.45 (s, 3H), 2.44 (s, 3H), 2.30 (s, 3H). ^13^C NMR (126 MHz, CDCl_3_) *δ* 163.0, 161.1, 154.3, 151.7, 141.7, 141.4, 136.9, 132.5, 132.3, 132.0, 130.0, 131.7, 129.8, 121.6, 115.5, 17.5, 16.8, 15.3, 14.5, 13.1. HRMS (ESI): *m*/*z* [M + H]^+^ calcd for C_20_H_22_^11^BF_3_N_2_^+^: 357.1744; found: 357.1752, *Δ* = −2.01 ppm.

#### 2-(4-Chlorophenyl)-5,5-difluoro-1,3,7,9,10-pentamethyl-5*H*-5^λ^,6^λ^-dipyrrolo[1,2-*c*:2′,1′-*f*] [1,3,2] diazaborinine (3i)

An orange solid (yield: 32.3 mg, 43%, single substitute: double substitute = 94 : 6), ^1^H NMR (400 MHz, CDCl_3_) *δ* 7.40 (d, *J* = 8.3 Hz, 2H), 7.14 (d, *J* = 8.3 Hz, 2H), 6.10 (s, 1H), 2.64 (s, 3H), 2.54 (s, 3H), 2.45 (s, 3H), 2.44 (s, 3H), 2.30 (s, 3H). ^13^C NMR (126 MHz, CDCl_3_) *δ* 154.5, 151.4, 141.8, 141.6, 136.7, 133.1, 132.6, 132.4, 132.0, 131.7, 128.6, 121.7, 17.5, 16.8, 15.3, 14.5, 13.1. HRMS (ESI): *m*/*z* [M + H]^+^ calcd for C_20_H_21_^11^BClF_2_N_2_^+^: 373.1449; found: 373.1452, *Δ* = −0.33 ppm.

#### 2-(4-Bromophenyl)-5,5-difluoro-1,3,7,9,10-pentamethyl-5*H*-5^λ^,6^λ^-dipyrrolo[1,2-*c*:2′,1′-*f*] [1,3,2]diazaborinine (3j)

An orange solid (yield: 28.9 mg, 35%, single substitute: double substitute = 93 : 7), ^1^H NMR (400 MHz, CDCl_3_) *δ* 7.55 (d, *J* = 8.3 Hz, 2H), 7.07 (d, *J* = 8.3 Hz, 2H), 6.09 (s, 1H), 2.63 (s, 3H), 2.54 (s, 3H), 2.45 (s, 3H), 2.43 (s, 3H), 2.30 (s, 3H). ^13^C NMR (126 MHz, CDCl_3_) *δ* 154.8, 151.5, 142.0, 141.9, 136.9, 133.1, 132.9, 132.3, 131.9, 131.8, 122.0, 121.5, 17.7, 17.1, 15.5, 14.7, 13.3. HRMS (ESI): *m*/*z* [M + H]^+^ calcd for C_20_H_21_^11^BClF_2_N_2_^+^: 373.1449; found: 373.1452, *Δ* = −0.33 ppm.

#### 2-(3-Bromophenyl)-5,5-difluoro-1,3,7,9,10-pentamethyl-5*H*-5^λ^,6^λ^-dipyrrolo[1,2-c:2′,1′-f] [1,3,2] diazaborinine (3k)

An orange solid (yield: 29.3 mg, 35%, single substitute: double substitute = 90 : 10), ^1^H NMR (400 MHz, CDCl_3_) *δ* 7.48 (d, *J* = 7.9 Hz, 1H), 7.37 (s, 1H), 7.30 (t, *J* = 7.8 Hz, 1H), 7.14 (d, *J* = 7.5 Hz, 1H), 6.11 (s, 1H), 2.65 (s, 3H), 2.55 (s, 3H), 2.47 (s, 3H), 2.45 (s, 3H), 2.32 (s, 3H). ^13^C NMR (126 MHz, CDCl_3_) *δ* 154.8, 151.2, 141.9, 141.8, 136.7, 136.2, 133.3, 132.7, 131.8, 131.6, 130.2, 129.9, 129.1, 122.4, 121.8, 17.5, 16.9, 15.3, 14.6, 13.1. HRMS (ESI): *m*/*z* [M + H]^+^ calcd for C_20_H_21_^11^B^79^BrF_2_N_2_^+^: 417.0944; found: 417.943, *Δ* = −0.28 ppm.

#### 5,5-Difluoro-1,3,7,9,10-pentamethyl-2,8-diphenyl-5*H*-4^λ^,5^λ^-dipyrrolo[1,2-*c*:2′,1′-*f*][1,3,2]diazaborinine (4a)

An orange solid, (yield: 41.4 mg, 50%) ^1^H NMR (400 MHz, CDCl_3_) *δ* 7.47 (t, *J* = 7.5 Hz, 4H), 7.38 (t, *J* = 7.5 Hz, 2H), 7.26 (d, *J* = 7.0 Hz, 4H), 2.75 (s, 3H), 2.53 (s, 6H), 2.38 (s, 6H). ^13^C NMR (126 MHz, CDCl_3_) *δ* 152.4, 141.8, 137.1, 133.7, 132.2, 130.4, 128.4, 127.1, 17.3, 15.5, 13.3. HRMS (ESI): *m*/*z* [M + H]^+^ calcd for C_26_H_26_^11^BF_2_N_2_^+^: 415.2152; found: 415.2153, *Δ* = −0.4 ppm.

#### 5,5-Difluoro-1,3,7,9,10-pentamethyl-2,8-di-*p*-tolyl-5*H*-4^λ^,5^λ^-dipyrrolo[1,2-*c*:2′,1′-*f*][1,3,2]diazaborinine (4b)

An orange solid, (yield: 15.2 mg, 17%) ^1^H NMR (400 MHz, CDCl_3_) *δ* 7.25 (d, *J* = 7.9 Hz, 5H), 7.11 (d, *J* = 7.9 Hz, 4H), 2.70 (s, 3H), 2.49 (s, 6H), 2.41 (s, 6H), 2.34 (s, 6H). ^13^C NMR (126 MHz, CDCl_3_) *δ* 152.4, 141.5, 137.0, 136.8, 133.5, 132.2, 130.8, 130.3, 129.1, 77.3, 77.0, 76.8, 29.7, 21.3, 17.2, 15.5, 13.3. HRMS (ESI): *m*/*z* [M + H]^+^ calcd for C_28_H_30_^11^BF_2_N_2_^+^: 443.2465; found: 443.2461, *Δ* = 0.86 ppm.

#### 5,5-Difluoro-1,3,7,9,10-pentamethyl-2,8-di-*m*-tolyl-5*H*-4^λ^,5^λ^-dipyrrolo[1,2-*c*:2′,1′-*f*][1,3,2]diazaborinine (4c)

An orange solid, (yield: 20.5 mg, 23%) ^1^H NMR (400 MHz, CDCl_3_) *δ* 7.33 (t, *J* = 7.5 Hz, 2H), 7.17 (d, *J* = 7.5 Hz, 2H), 7.05–7.00 (m, 4H), 2.71 (s, 3H), 2.49 (s, 6H), 2.41 (s, 6H), 2.35 (s, 6H). ^13^C NMR (126 MHz, CDCl_3_) *δ* 152.3, 141.5, 137.9, 137.0, 133.7, 132.2, 131.0, 128.2, 127.8, 127.4, 21.5, 17.2, 15.5, 13.3. HRMS (ESI): *m*/*z* [M − F]^+^ calcd for C_28_H_29_^11^BFN_2_^+^: 423.2402; found: 423.2409, *Δ* = −1.64 ppm.

#### 5,5-Difluoro-2,8-bis(4-methoxyphenyl)-1,3,7,9,10-pentamethyl-5*H*-4^λ^,5^λ^-dipyrrolo[1,2-*c*:2′,1′-*f*] [1,3,2] diazaborinine (4e)

An orange solid, (yield: 17.7 mg, 19%) ^1^H NMR (400 MHz, CDCl_3_) *δ* 7.14 (d, *J* = 8.7 Hz, 4H), 6.98 (d, *J* = 8.7 Hz, 4H), 3.86 (s, 6H), 2.70 (s, 3H), 2.48 (s, 6H), 2.33 (s, 6H). ^13^C NMR (126 MHz, CDCl_3_) *δ* 158.7, 152.4, 141.4, 137.0, 133.2, 132.1, 131.5, 126.0, 113.9, 55.3, 17.2, 15.5, 13.3. HRMS (ESI): *m*/*z* [M + H]^+^ calcd for C_28_H_29_^11^BF_2_N_2_O_2_^+^: 455.2301; found: 455.2307, *Δ* = −1.46 ppm.

#### 5,5-Difluoro-1,3,7,9,10-pentamethyl-2,8-bis(4-(trifluoromethyl)phenyl)-5*H*-4^λ^,5^λ^-dipyrrolo[1,2-*c*:2′,1′-*f*] [1,3,2]diazaborinine (4f)

An orange solid, (yield: 21.1 mg, 19%) ^1^H NMR (400 MHz, CDCl_3_) *δ* 7.71 (d, *J* = 7.9 Hz, 4H), 7.36 (d, *J* = 7.9 Hz, 4H), 2.74 (s, 3H), 2.50 (s, 6H), 2.36 (s, 6H). ^13^C NMR (126 MHz, CDCl_3_) *δ* 152.4, 142.6, 137.6, 137.5, 132.4, 130.7, 129.5, 129.3, 125.4, 123.1, 17.4, 15.5, 13.3. HRMS (ESI): *m*/*z* [M − F]^+^ calcd for C_28_H_23_^11^BF_7_N_2_^+^: 531.1837; found: 531.1832, *Δ* = 0.98 ppm.

#### Dimethyl 4,4′-(5,5-difluoro-1,3,7,9,10-pentamethyl-5*H*-4^λ^, 5^λ^-dipyrrolo[1,2-*c*:2′,1′-*f*][1,3,2] diazaborinine-2,8-diyl) dibenzoate (4g)

An orange solid, (yield: 29.8 mg, 28%) ^1^H NMR (400 MHz, CDCl_3_) *δ* 8.12 (d, *J* = 7.9 Hz, 4H), 7.32 (d, *J* = 7.9 Hz, 4H), 3.96 (s, 6H), 2.74 (s, 3H), 2.51 (s, 6H), 2.37 (s, 6H). ^13^C NMR (126 MHz, CDCl_3_) *δ* 166.9, 152.4, 142.4, 138.7, 137.4, 132.8, 132.5, 130.4, 129.7, 128.9, 52.2, 17.4, 15.6, 13.3. HRMS (ESI): *m*/*z* [M − F]^+^ calcd for C_30_H_29_^11^BFN_2_O_4_^+^: 511.2199; found: 511.2196, *Δ* = 0.55 ppm.

#### 5,5-Difluoro-2,8-bis(4-fluorophenyl)-1,3,7,9,10-pentamethyl-5*H*-4^λ^, 5^λ^-dipyrrolo [1,2-*c*:2′,1′-*f*] [1,3,2] diazaborinine (4h)

An orange solid, (yield: 18.2 mg, 20%) ^1^H NMR (400 MHz, CDCl_3_) *δ* 7.16 (m, 8H), 2.71 (s, 3H), 2.47 (s, 6H), 2.32 (s, 6H). ^13^C NMR (126 MHz, CDCl_3_) *δ* 163.1, 161.1, 152.4, 142.0, 137.3, 132.7, 132.1, 129.7, 115.5, 115.4, 17.3, 15.5, 13.2. HRMS (ESI): *m*/*z* [M − F]^+^ calcd for C_26_H_24_^11^BF_3_N_2_^+^: 431.2972; found: 431.2970, *Δ* = 0.46 ppm.

#### 2,8-Bis(4-chlorophenyl)-5,5-difluoro-1,3,7,9,10-pentamethyl-5*H*-4^λ^, 5^λ^-dipyrrolo [1,2-*c*:2′,1′-*f*] [1,3,2] diazaborinine (4i)

An orange solid, (yield: 49.3 mg, 51%) ^1^H NMR (400 MHz, CDCl_3_) *δ* 7.42 (d, *J* = 8.4 Hz, 4H), 7.16 (d, *J* = 8.4 Hz, 4H), 2.71 (s, 3H), 2.48 (s, 6H), 2.33 (s, 6H). ^13^C NMR (126 MHz, CDCl_3_) *δ* 152.3, 142.1, 137.3, 133.3, 132.5, 132.2, 131.7, 128.7, 17.3, 15.5, 13.2. HRMS (ESI): *m*/*z* [M − F]^+^ calcd for C_26_H_23_^11^BCl_2_FN_2_^+^: 463.1310; found: 463.1300, *Δ* = −2.09 ppm.

#### 2,8-Bis(4-bromophenyl)-5,5-difluoro-1,3,7,9,10-pentamethyl-5*H*-4^λ^, 5^λ^-dipyrrolo [1,2-*c*:2′,1′-*f*] [1,3,2] diazaborinine (4j)

An orange solid, (yield: 27.2 mg, 24%) ^1^H NMR (400 MHz, CDCl_3_) *δ* 7.57 (d, *J* = 8.4 Hz, 4H), 7.10 (d, *J* = 8.4 Hz, 4H), 2.71 (s, 3H), 2.48 (s, 6H), 2.33 (s, 6H). ^13^C NMR (126 MHz, CDCl_3_) *δ* 152.3, 142.1, 137.2, 132.7, 132.5, 132.3, 132.0, 131.7, 121.4, 17.3, 15.5, 13.2. HRMS (ESI): *m*/*z* [M + H]^+^ calcd for C_26_H_23_^11^B^79^Br_2_F_2_N_2_^+^: 570.0284; found: 570.0285, *Δ* = 0.26 ppm.

#### 2,8-Bis(3-bromophenyl)-5,5-difluoro-1,3,7,9,10-pentamethyl-5*H*-4^λ^, 5^λ^-dipyrrolo [1,2-*c*:2′,1′-*f*] [1,3,2] diazaborinine (4k)

An orange solid, (yield: 22.3 mg, 20%) ^1^H NMR (400 MHz, CDCl_3_) *δ* 7.54–7.50 (m, *J* = 7.8 Hz, 2H), 7.41 (t, 2H), 7.34 (t, *J* = 7.8 Hz, 2H), 7.19 (d, *J* = 7.8 Hz, 2H), 2.74 (s, 3H), 2.51 (s, 6H), 2.37 (s, 6H). ^13^C NMR (126 MHz, CDCl_3_) *δ* 152.4, 142.3, 137.4, 136.0, 133.2, 132.3, 130.3, 130.0, 129.1, 122.5, 17.3, 15.5, 13.2. HRMS (ESI): *m*/*z* [M − F]^+^ calcd for C_26_H_23_^11^B^79^Br_2_FN_2_^+^: 551.0300; found: 551.0293, *Δ* = −1.20 ppm.

#### Methyl4-(5,5-difluoro-8-(4-methoxyphenyl)-1,3,7,9,10-pentamethyl-5*H*-5^λ^,6^λ^-dipyrrolo[1,2-*c*:2′,1′-*f*] [1,3,2] diazaborinin-2-yl)benzoate (5)

An orange solid (second step's yield: 52%). ^1^H NMR (400 MHz, CDCl_3_) *δ* 8.11 (d, *J* = 7.3 Hz, 2H), 7.32 (d, *J* = 7.4 Hz, 2H), 7.14 (d, *J* = 7.6 Hz, 2H), 6.99 (d, *J* = 7.7 Hz, 2H), 3.95 (s, 3H), 3.86 (s, 3H), 2.72 (s, 3H), 2.50 (s, 6H), 2.35 (s, 6H). ^13^C NMR (126 MHz, CDCl_3_) *δ* 166.6, 158.4, 153.6, 150.5, 141.4, 138.6, 137.5, 135.9, 133.5, 132.2, 131.7, 131.6, 131.0, 130.0, 129.2, 128.3, 125.3, 113.5, 54.9, 51.8, 16.9, 15.2, 15.0, 13.0, 12.8. HRMS (ESI): *m*/*z* [M − F]^+^ calcd for C_29_H_29_^11^BFN_2_O_3_^+^: 483.2250; found: 483.2258, *Δ* = 1.66 ppm.

#### 5,5-Difluoro-1,3,7,9-tetramethyl-2,10-diphenyl-5*H*-5^λ^,6^λ^-dipyrrolo[1,2-*c*:2′,1′-*f*][1,3,2]diazaborinine (7)

An orange solid (yield: 37.0 mg, 46%), ^1^H NMR (400 MHz, CDCl_3_) *δ* 7.55–7.48 (m, 3H), 7.40 (t, *J* = 7.4 Hz, 2H), 7.37–7.29 (m, 3H), 7.19–7.15 (m, 2H), 6.03 (s, 1H), 2.61 (s, 3H), 2.55 (s, 3H), 1.41 (s, 3H), 1.32 (s, 3H). ^13^C NMR (126 MHz, CDCl_3_) *δ* 155.6, 154.0, 143.2, 142.0, 139.2, 135.2, 133.7, 133.7, 131.7, 131.0, 130.2, 129.2, 129.0, 128.3, 128.0, 127.0, 121.4, 14.6, 14.4, 13.3, 12.7. HRMS (ESI): *m*/*z* [M + H]^+^ calcd for C_25_H_24_^11^BF_2_N_2_^+^: 401.2001; found: 401.2008, *Δ* = 1.20 ppm.

#### (*E*)-5,5-Difluoro-1,3,7,9,10-pentamethyl-2-styryl-5*H*-5^λ^,6^λ^-dipyrrolo[1,2-*c*:2′,1′-*f*][1,3,2]diazaborinine (9a)

A brownish red solid (yield: 27.7 mg, 38%, single substitute: double substitute = 98 : 2). ^1^H NMR (400 MHz, CDCl_3_) *δ* 7.48 (d, *J* = 7.3 Hz, 2H), 7.36 (t, *J* = 7.3 Hz, 2H), 7.26 (t, 1H), 6.95 (d, *J* = 16.5 Hz, 1H), 6.68 (d, *J* = 16.5 Hz, 1H), 6.08 (s, 1H), 2.67 (s, 3H), 2.64 (s, 3H), 2.53 (s, 3H), 2.50 (s, 3H), 2.43 (s, 3H). ^13^C NMR (126 MHz, CDCl_3_) *δ* 154.0, 152.9, 141.3, 141.2, 137.8, 136.6, 132.5, 132.0, 131.9, 128.7, 128.5, 127.5, 126.1, 121.5, 119.9, 77.3, 77.0, 76.8, 17.5, 17.0, 15.3, 14.5, 13.8. HRMS (ESI): *m*/*z* [M − F]^+^ calcd for C_22_H_23_^11^BFN_2_^+^: 345.1933; found: 345.1935,*Δ* = −0.73 ppm.

#### (*E*)-5,5-Difluoro-1,3,7,9,10-pentamethyl-2-(4-methylstyryl)-5*H*-5^λ^,6^λ^-dipyrrolo[1,2-*c*:2′,1′-*f*][1,3,2]diazaborinine (9b)

A brownish red solid (yield: 18.6 mg, 25%, single substitute: double substitute = 95 : 5). ^1^H NMR (400 MHz, CDCl_3_) *δ* 7.38 (d, *J* = 8.0 Hz, 2H), 7.17 (d, *J* = 8.0 Hz, 2H), 6.90 (d, *J* = 16.5 Hz, 1H), 6.65 (d, *J* = 16.5 Hz, 1H), 6.07 (s, 1H), 2.66 (s, 3H), 2.64 (s, 3H), 2.53 (s, 3H), 2.49 (s, 3H), 2.43 (s, 3H), 2.36 (s, 3H). ^13^C NMR (126 MHz, CDCl_3_) *δ* 153.8, 153.1, 141.2, 141.0, 137.4, 136.5, 135.0, 132.5, 132.1, 129.4, 128.8, 126.1, 121.4, 118.9, 110.0, 21.2, 17.5, 16.9, 15.3, 14.5, 13.8. HRMS (ESI): *m*/*z* [M − F]^+^ calcd for C_23_H_25_^11^BFN_2_^+^: 359.2089; found: 359.2098, *Δ* = −2.39 ppm.

#### (*E*)-5,5-Difluoro-2-(4-fluorostyryl)-1,3,7,9,10-pentamethyl-5*H*-5^λ^,6^λ^-dipyrrolo[1,2-*c*:2′,1′-*f*][1,3,2]diazaborinine (9c)

A brownish red solid (yield: 16.6 mg, 22%, single substitute: double substitute = 96 : 4). ^1^H NMR (400 MHz, CDCl_3_) *δ* 7.47 (dd, *J* = 8.6 Hz, 2H), 7.07 (t, *J* = 8.6 Hz, 2H), 6.89 (d, *J* = 16.5 Hz, 1H), 6.66 (d, *J* = 16.5 Hz, 1H), 6.11 (s, 1H), 2.68 (s, 3H), 2.67 (s, 3H), 2.56 (s, 3H), 2.52 (s, 3H), 2.46 (s, 3H). ^13^C NMR (126 MHz, CDCl_3_) *δ* 163.3, 161.3, 154.2, 152.7, 141.3, 136.5, 133.9, 132.6, 133.0, 130.6, 128.3, 127.6, 121.6, 119.7, 115.7, 115.5, 17.5, 17.0, 15.3, 14.5, 13.8. HRMS (ESI): *m*/*z* [M − F]^+^ calcd for C_22_H_22_^11^BF_2_N_2_^+^: 363.1839; found: 363.1834, *Δ* = 1.15 ppm.

#### (*E*)-2-(4-Chlorostyryl)-5,5-difluoro-1,3,7,9,10-pentamethyl-5*H*-5^λ^,6^λ^-dipyrrolo[1,2-*c*:2′,1′-*f*] [1,3,2]diazaborinine (9d)

A brownish red solid (yield: 53.4 mg, 67%, single substitute: double substitute = 96 : 4). ^1^H NMR (400 MHz, CDCl_3_) *δ* 7.39 (d, *J* = 8.5 Hz, 2H), 7.31 (d, *J* = 8.5 Hz, 2H), 6.92 (d, *J* = 16.5 Hz, 1H), 6.62 (d, *J* = 16.5 Hz, 1H), 6.08 (s, 1H), 2.66 (s, 3H), 2.63 (s, 3H), 2.53 (s, 3H), 2.48 (s, 3H), 2.42 (s, 3H). ^13^C NMR (126 MHz, CDCl_3_) *δ* 154.4, 152.6, 141.5, 141.4, 136.5, 136.3, 133.0, 132.7, 131.9, 130.3, 128.8, 128.1, 127.3, 121.7, 120.6, 17.5, 17.0, 15.2, 14.5, 13.8. HRMS (ESI): *m*/*z* [M + H]^+^ calcd for C_22_H_22_^11^BCl_2_F_2_N_2_^+^: 398.1527; found: 398.1522, *Δ* = −1.42 ppm.

#### (*E*)-2-(4-Bromostyryl)-5,5-difluoro-1,3,7,9,10-pentamethyl-5*H*-5^λ^,6^λ^-dipyrrolo [1,2-*c*:2′,1′-*f*] [1,3,2]diazaborinine (9e)

A brownish red solid (yield: 24.7 mg, 28%, single substitute: double substitute = 94 : 6). ^1^H NMR (400 MHz, CDCl_3_) *δ* 7.49 (d, *J* = 8.4 Hz, 2H), 7.36 (d, *J* = 8.5 Hz, 2H), 6.97 (d, *J* = 16.5 Hz, 1H), 6.62 (d, *J* = 16.5 Hz, 1H), 6.11 (s, 1H), 2.68 (s, 3H), 2.66 (s, 3H), 2.56 (s, 3H), 2.51 (s, 3H), 2.45 (s, 3H). ^13^C NMR (126 MHz, CDCl_3_) *δ* 154.4, 152.5, 141.5, 141.4, 136.7, 136.5, 132.7, 131.9, 131.8, 130.3, 128.0, 127.6, 121.7, 121.1, 120.7, 17.5, 17.0, 15.3, 14.5, 13.8. HRMS (ESI): *m*/*z* [M − F]^+^ calcd for C_22_H_22_^11^BBrFN_2_^+^: 423.1038; found: 423.1035, *Δ* = −0.63 ppm.

#### (*E*)-5,5-Difluoro-1,3,7,9,10-pentamethyl-2-(pent-1-en-1-yl)-5*H*-5^λ^,6^λ^-dipyrrolo[1,2-*c*:2′,1′-*f*] [1,3,2] diazaborinine (9f)

A brownish red solid (yield: 33.6 mg, 51%, single substitute: double substitute = 89 : 11). ^1^H NMR (400 MHz, CDCl_3_) *δ* 6.16 (d, *J* = 16.1 Hz, 1H), 6.03 (s, 1H), 5.75 (dt, *J* = 16.1, 6.9 Hz, 1H), 2.59 (s, 3H), 2.56 (s, 3H), 2.51 (s, 3H), 2.40 (s, 3H), 2.38 (s, 3H), 2.21 (qd, *J* = 6.9, 2H), 1.50 (m, *J* = 7.4 Hz, 2H), 0.97 (t, *J* = 7.4 Hz, 3H). ^13^C NMR (126 MHz, CDCl_3_) *δ* 153.2, 152.9, 141.0, 140.3, 136.5, 135.3, 132.1, 132.0, 129.5, 121.0, 120.6, 35.9, 22.7, 17.4, 16.8, 15.1, 14.4, 13.7, 13.6. HRMS (ESI): *m*/*z* [M + H]^+^ calcd for C_19_H_26_^11^BF_2_N_2_^+^: 331.2152; found: 331.2159, *Δ* = −0.73 ppm.

## Conflicts of interest

There are no conflicts to declare.

## Supplementary Material

RA-008-C7RA13070H-s001
