# Supplementary material for: Direct C–H functionalization of difluoroboron dipyrromethenes (BODIPYs) at β-position by iodonium salts
Source: RSC Adv. 2018 Feb 1;8(10):5542–9. doi: 10.1039/c7ra13070h (PMC9078099; doi:10.1039/c7ra13070h)
Supplement: RA-008-C7RA13070H-s001 [file RA-008-C7RA13070H-s001.pdf]

Electronic Supplementary Material (ESI) for RSC Advances.
This journal is © The Royal Society of Chemistry 2018

Electronic Supplementary Information (ESI)

for

Direct C–H Functionalization of Difluoroboron Dipyrromethenes (BODIPYs) at β-position by Iodonium Salts and Its Application

Wenming Ren,‡[a] Huaijiang Xiang,‡[b] Chengyuan Peng,[a] Zulipali Musha,[ac] Jingjing Chen,[ac] Xin Li,[b] Ruimin Huang,*[ac] and Youhong Hu*[ac]

[a]*State Key Laboratory of Drug Research, Shanghai Institute of Materia Medica, Chinese Academy of Sciences, 555 Zuchongzhi Road, Shanghai 201203, China.*

[b]*College of Pharmaceutical Sciences, Zhejiang University, 866 Yuhangtang Road, Hangzhou 310058, China*

[c]*University of Chinese Academy of Sciences, 19 Yuquan Road, Beijing 110039, China*

E-mail: yhhu@simm.ac.cn, rmhuang@simm.ac.cn

Table of contents

1. Experimental Details and Compound Characterization..3

1.1. Materials and Instrumentation..3

1.2. Experimental procedures for the synthesis of SA1-17................... … … … … 3

2. Optical properties of BODIPY dyes………………………………………………….7

3. Cell viability assay for photocytotoxic effect……………………………………..…..13

4. ^{1}H and ^{13}C NMR Spectra……………………………………………………..……13

1. Experimental Details and Compound Characterization

1.1. Materials and Instrumentation

All commercially available reagents were used as received. Unless otherwise specified, all reactions were carried out under an atmosphere of argon in oven-dried glassware with magnetic stirring. ^{1}H NMR spectra and proton-decoupled ^{13}C NMR spectra were obtained on a 400 MHz or 500 MHz Bruker NMR spectrometer. ^{1}H Chemical shifts (δ) are reported in parts per million (ppm) relative to TMS (s, δ 0). Multiplicities are given as: s (singlet), d (doublet), t (triplet) and m (multiplet). Complex splitting will be described by a combination of these abbreviations, i.e. dd (doublet of doublets). ^{13}C NMR chemical shifts are reported relative to $CDCl_3$ (t, δ 77.4). High-resolution mass spectra were recorded on positive ESI mode. Chromatographic purifications were performed by flash chromatography with silica gel (40–63 μm) packed in glass columns. The eluting solvent for the purification of each compound was determined by thin-layer chromatography (TLC) on glass plates coated with silica gel 60 F254 and visualized by ultraviolet light. High-resolution mass data were obtained on an Agilent 6224 Accurate-Mass TOF LC/MS. Absorption spectra were acquired using a a Varian Cary 300 spectrophotometer. Fluorescence measurements were carried out on a Horiba FluoroMax 4 spectrometer. Quantum yields were determined in reference to either Fluorescein or Rhodamine 6G and corrected for solvent refractive index. The extinction coefficients were determined through Beer's Law plots. All data were measured at room temperature. Human breast cancer cell line MDA-MB-231 and human epidermoid carcinoma cell line A-431 were obtained from the American Type Culture Collection (Manassas, VA, USA) and were cultured in DMEM (high glucose) and RPMI 1640 medium respectively, supplemented with 10% fetal bovine serum (Hyclone, Logan, UT). Cells were incubated at 37℃ in 5% CO_2 in air.

1.2. Experimental procedures for the synthesis of 3a-k,4a-k and 6a-f

Diaryliodonium salts **SA1-11** were prepared according to the literature procedures.[1-2]

mesityl(phenyl)- 3$^{\lambda}$-iodanyl trifluoromethanesulfonate (SA1)

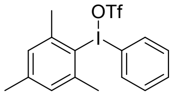

A white powder. ^{1}H NMR (400 MHz, $CDCl_3$) δ 7.72 – 7.67 (m, 2 H), 7.56 – 7.49 (m, 1 H), 7.44 – 7.38 (m, 2 H), 7.11 (s, 2 H), 2.63 (s, 6 H), 2.36 (s, 3 H).

mesityl(p-tolyl)- 3$^{\lambda}$ -iodanyl trifluoromethanesulfonate (SA2)

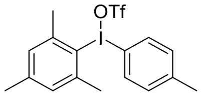

A white powder. ^{1}H NMR (400 MHz, $CDCl_3$) δ 7.57 (d, *J* = 8.5 Hz, 2 H), 7.22 (d, *J* = 8.5 Hz, 2 H), 7.10 (s, 2 H), 2.63 (s, 6 H), 2.38 (s, 3 H), 2.36 (s, 3 H).

mesityl(m-tolyl)- 3$^{\lambda}$-iodanyl trifluoromethanesulfonate (SA3)

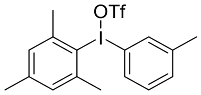

A white powder. ^{1}H NMR (400 MHz, $CDCl_3$) δ 7.59 (s, 1 H), 7.40 (d, *J* = 8.1 Hz, 1 H), 7.33 (d, *J* = 7.5

Hz, 1 H), 7.27 (t, J = 8.1 Hz &7.5 Hz, 1 H), 7.11 (s, 2 H), 2.63 (s, 6 H), 2.36 (s, 6 H).

mesityl(o-tolyl)- 3^{λ}-iodanyl trifluoromethanesulfonate (SA4)

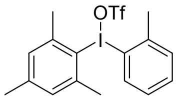

A white powder. ^{1}H NMR (400 MHz, $CDCl_3$) δ 7.50 – 7.41 (m, 3 H), 7.20 – 7.15 (m, 1 H), 7.12 (s, 2 H), 2.60 (s, 9 H), 2.36 (s, 3 H).

methyl 4-(mesityl(((trifluoromethyl)sulfonyl)oxy)- 3^{λ}-iodanyl)benzoate (SA5)

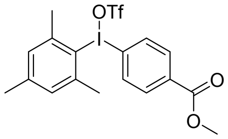

A white powder. ^{1}H NMR (400 MHz, $CDCl_3$) δ 8.01 (d, J = 8.7 Hz, 2 H), 7.78 (d, J = 8.7 Hz, 2 H), 7.11 (s, 2 H), 3.91 (s, 3 H), 2.62 (s, 6 H), 2.36 (s, 3 H).

(4-fluorophenyl)(mesityl)- 3^{λ}-iodanyl trifluoromethanesulfonate (SA6)

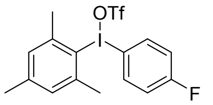

A white powder. ^{1}H NMR (400 MHz, $CDCl_3$) δ 7.74 (m, 2 H), 7.10 (m, 4 H), 2.63 (s, 6 H), 2.35 (s, 3 H).

(4-chlorophenyl)(mesityl)- 3^{λ}-iodanyl trifluoromethanesulfonate (SA7)

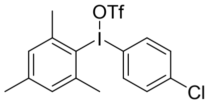

A white powder. ^{1}H NMR (400 MHz, $CDCl_3$) δ 7.65 (d, J = 8.8 Hz, 2 H), 7.36 (d, J = 8.8 Hz, 2 H), 7.10 (s, 2 H), 2.62 (s, 6 H), 2.35 (s, 3 H).

(4-bromophenyl)(mesityl)- 3^{λ}-iodanyl trifluoromethanesulfonate (SA8)

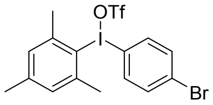

A white powder. ^{1}H NMR (400 MHz, $CDCl_3$) δ 7.57 (d, J = 8.8 Hz, 2 H), 7.50 (d, J = 8.8 Hz, 2 H), 7.09 (s, 2 H), 2.62 (s, 6 H), 2.35 (s, 3 H).

(3-bromophenyl)(mesityl)- 3^{λ}-iodanyl trifluoromethanesulfonate (SA9)

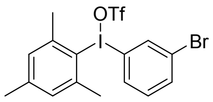

A white powder. ^{1}H NMR (400 MHz, $CDCl_3$) δ 7.78 (t, 1 H), 7.69 (d, J = 8.2 Hz, 1 H), 7.64 (d, J = 8.7 Hz, 1 H), 7.29 – 7.25 (t, J = 8.2 Hz & 8.7 Hz, 1 H), 7.11 (s, 2 H), 2.63 (s, 6 H), 2.36 (s, 3 H).

mesityl(4-(trifluoromethyl)phenyl)- 3^{λ}-iodanyl trifluoromethanesulfonate (SA10)

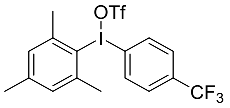

A white powder. ^{1}H NMR (400 MHz, $CDCl_3$) δ 7.81 (d, J = 8.3 Hz, 2 H), 7.66 (d, J = 8.3 Hz, 2 H), 7.15 (s, 2 H), 2.63 (s, 6 H), 2.39 (s, 3 H).

mesityl(4-methoxyphenyl)- 3$^{\lambda}$-iodanyl trifluoromethanesulfonate (SA11)

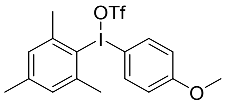

A white powder. ^{1}H NMR (400 MHz, $CDCl_3$) δ 7.67 (d, J = 9.1 Hz, 2 H), 7.07 (s, 2 H), 6.92 (d, J = 9.1 Hz, 2 H), 3.80 (s, 3 H), 2.64 (s, 6 H), 2.33 (s, 3 H).

The general synthetic method for SA12-17

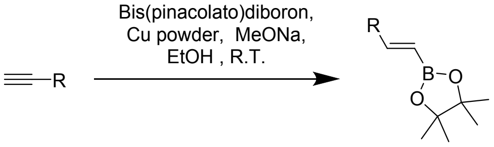

Micro copper powder (0.5 mmol) and sodium methanolate (0.5 mmol) were added to a stirred solution of phenylacetylene or alkyne (5.0 mmol) and bis(pinacolato)diboron (7.5 mmol) in 100 mL anhydrous ethanol at room temperature. After the phenylacetylene or alkyne was completely consumed according to TLC, the product was extracted with brine and acetic ether. The organic layer was dried with Na_2SO_4 and concentrated. The residue was purified by silica gel column chromatography (hexane: acetic ether = 50 : 1 to 20:1) to afford pure product.

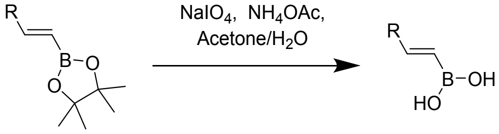

To a solution of first step product (1 mmol) in acetone (30 mL) and water (15 mL) were added NH_4OAc (6 mmol) and $NaIO_4$ (6 mmol). The resulting reaction mixture was stirred at room temperature overnight until the material was consumed. The reaction mixture was diluted with Et_2O, and filtered through a pad of cellite. The filtrate was concentrated to give aryl boronic as a solid.

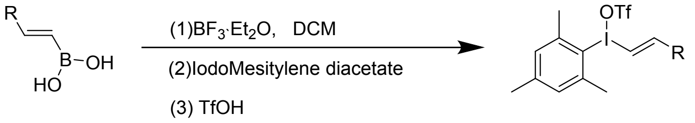

The corresponding alkenyl boronic acid (1.00 mmol) was suspended in dry dichloromethane (30 mL) at 0 °C. Boron trifluoride diethyl etherate (1.2 mmol) was added dropwise via syringe and stirred at 0 °C for 15 min or until all the boronic acid had dissolved. Iodo-mesitylene diacetate (1.2 mmol) was added as a solution in dichloromethane (15 mL) via syringe. The reaction mixture was stirred for 1 hour or until complete consumption of the iodoarene diacetate, at which point trifluoromethanesulfonic acid (1.2 mmol) was added. After stirring 15 min, H_2O (50 mL) was added. The aqueous phase was extracted with dichloromethane (3 * 50 mL) and the combined organic phases were dried by Na_2SO_4 and concentrated. The crude residue was then recrystallized from dichloromethane/Et_2O or triturated with Et_2O to obtain the desired compound as a white powder.

SA12 (E)-mesityl(styryl)- 3$^{\lambda}$-iodanyl trifluoromethanesulfonate.

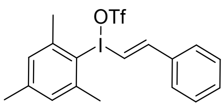

A white powder.(219mg, 44%) ^{1}H NMR (400 MHz, $CDCl_3$) δ 7.38 – 7.29 (m, 6 H), 7.14 (s, 2 H), 6.97 (d, J = 14.4 Hz, 1 H), 2.64 (s, 6 H), 2.38 (s, 3 H). ^{13}C NMR (126 MHz, $CDCl_3$) δ 144.6, 143.9, 143.0, 134.4, 130.6, 130.3, 129.1, 127.6, 121.6, 119.0, 116.8, 97.1, 27.0, 21.2.

SA13 (E)-(4-fluorostyryl)(mesityl)- 3$^\lambda$-iodanyl trifluoromethanesulfonate.

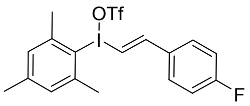

A white powder(332mg, 64%). ^{1}H NMR (400 MHz, $CDCl_3$) δ 7.34 (m, 2 H), 7.28 – 7.24 (m, 1 H), 7.14 (s, 2 H), 7.08 – 6.96 (m, 3 H), 2.64 (s, 6 H), 2.38 (s, 3 H). ^{13}C NMR (126 MHz, $CDCl_3$) δ 165.0, 163.0, 144.7, 143.2, 143.0, 130.4, 129.7, 129.6, 116.3, 116.1, 96.1, 27.0, 21.2.

SA14 (E)-(4-chlorostyryl)(mesityl)- 3$^\lambda$-iodanyl trifluoromethanesulfonate

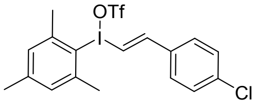

A white powder(420mg，79%). ^{1}H NMR (400 MHz, $CDCl_3$) δ 7.36 – 7.26 (m, 5 H), 7.15 (s, 2 H), 6.96 (d, 1 H), 2.64 (s, 6 H), 2.39 (s, 3 H). ^{13}C NMR (126 MHz, $CDCl_3$) δ 144.8, 143.1, 136.8, 132.8, 130.4, 129.3, 128.9, 116.5, 97.2, 27.0, 21.2.

SA15 (E)-(4-bromostyryl)(mesityl)- 3$^\lambda$-iodanyl trifluoromethanesulfonate.

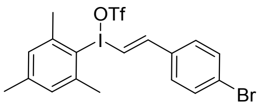

A white powder（340mg，59%）. ^{1}H NMR (400 MHz, $CDCl_3$) δ 7.50 – 7.45 (m, J = 8.5 Hz, 2 H), 7.35 (d, J = 14.4 Hz, 1 H), 7.20 (m, J = 8.5 Hz, 2 H), 7.15 (s, 2 H), 6.92 (d, J = 14.4 Hz, 1 H), 2.64 (s, 6 H), 2.39 (s, 3 H). ^{13}C NMR (126 MHz, $CDCl_3$) δ 144.7, 143.0, 142.7, 133.2, 132.3, 130.4, 129.0, 125.1, 116.6, 97.8, 27.0, 21.2.

SA16 (E)-mesityl(4-methylstyryl)- 3$^\lambda$-iodanyl trifluoromethanesulfonate.

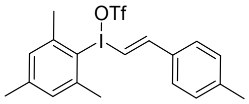

A white powder.(130mg, 25%). 1H NMR (400 MHz, CDCl3) δ 7.25 (d, J = 8.6 Hz, 1 H), 7.21 (s, 2 H), 7.14 (d, J = 8.6 Hz, 4 H), 6.97 (d, J = 14.3 Hz, 1 H), 2.64 (s, 6 H), 2.38 (s, 3 H), 2.33 (s, 3 H).^{13}C NMR (126 MHz, $CDCl_3$) δ 144.5, 144.4, 143.0, 141.3, 131.6, 130.3, 129.7, 127.6, 116.8, 95.5, 27.0, 21.4, 21.2.

SA17 (E)-mesityl(pent-1-en-1-yl)- 3$^\lambda$-iodanyl trifluoromethanesulfonate.

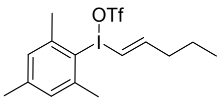

A white powder(160mg, 34 %). ^{1}H NMR (400 MHz, $CDCl_3$) δ 7.12 (s, 2 H), 6.59 (d, J = 13.8 Hz, 1 H), 6.27 – 6.18 (m, J = 13.8 Hz, 1 H), 2.59 (s, 6 H), 2.38 (s, 3 H), 2.27 – 2.18 (dd, 2 H), 1.42 (m, J =7.4 Hz, 2 H), 0.86 (t, J = 7.4 Hz, 3 H). ^{13}C NMR (126 MHz, $CDCl_3$) δ 147.0, 144.4, 142.8, 130.2, 121.6,

119.1, 116.5, 97.1, 36.7, 26.9, 21.1, 13.3.

Synthetic method of BODIPY 1 and 6

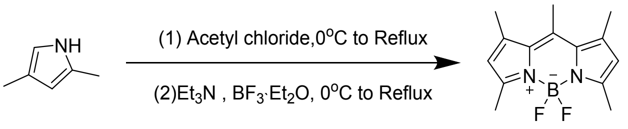

1

Acetyl chloride (3.4 mL, 47.77 mmol) was dropwisely added via syringe to a stirred solution of 2,4-dimethylpyrrole (10.8 mL, 105.1 mmol, 2.2 eq) in dry dichloromethane (200 mL) at 0 °C under an argon atmosphere. After acetyl chloride was completely added, the resulting solution was taken into 50-60 °C oil bath pan and stirred for additional 1-2 hours. After removed the most solvent in vacuo, the residue was dissolved in 500 mL dry dichloromethane/toluene (5/95, v/v). Trimethylamine (39.8 mL, 286.64 mmol) and $BF_3{\cdot}Et_2O$ (47.2 mL, 382.19 mmol) were sequentially added to the solution at 0 °C. Then the reaction was stirred at 50-60 °C for 1-2 h until the intermediate was consumed according to TLC. Then pour the reaction solution into ice-cold water and extract with dichloromethane. The organic layer was washed by water (3*500 mL), brine (3*500 mL), dried with Na_2SO_4 and concentrated. The residue was purified by silica gel column chromatography (hexane/acetic ether = 50/1 to 10/1) to afford pure product 1 (5.2 g, 42%) as a brownish red solid. ^1H NMR (400 MHz, $CDCl_3$) δ 6.08 (s, 2H), 2.60 (s, 3H), 2.54 (s, 6H), 2.44 (s, 6H).

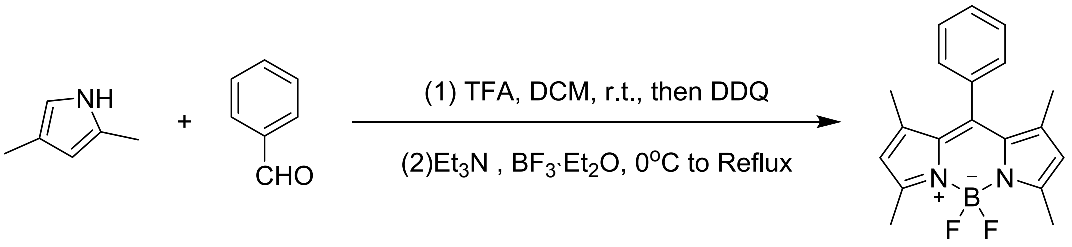

6

2,4-Dimethylpyrrole (2.15 g, 22 mmol) and benzaldehyde (1.06 g, 11 mmol) were dissolved in 150 mL CH_2Cl_2 with a catalytic amount of TFA (0.5 mL). The mixture was stirred for 16 h at r.t.. Then a solution of 2,3-dichloro-5,6-dicyanobenzoquinone (DDQ) (2.27 g, 10 mmol) was added, and the mixture was stirred for 15 min. Finally, $BF_3{\cdot}Et_2O$ (20 mL, excess) and triethylamine (20 mL, excess) were added, and the mixture was stirred for 3 h at r.t. The crude mixture was diluted with CH_2Cl_2 and washed with H_2O. The organic extracts were dried over $MgSO_4$, filtered and evaporated under reduced pressure. Flash chromatography (hexane:CH_2Cl_2 1:1). 2.00 g of compound 6 as an orange solid (61% yield). ^1H NMR (400 MHz, $CDCl_3$) δ 7.54 – 7.47 (m, 3H), 7.33 – 7.29 (m, 2H), 6.00 (s, 2H), 2.58 (s, 6H), 1.39 (s, 6H).

2. Optical properties of BODIPY dyes

Table S1 Photophysical properties of BODIPY dyes 1, 3a-k in CH_2Cl_2 at room temperature

dyes	λabs (nm)[a]	ε ($M^{-1}.cm^{-1}$)	λex (nm)[b]	λem (nm)[b]	ϕ_F[a,c]	Stokes shift (nm)
1	497	104500	496	509	0.79	13
3a	509	95000	507	532	0.61	25

3b	509	101000	508	538	0.47	30
3c	509	43500	508	534	0.54	26
3e	509	87600	508	552	0.32	44
3f	507	93500	507	527	0.59	20
3g	509	102100	507	530	0.59	23
3h	507	90000	507	531	0.57	24
3i	507	76500	508	532	0.50	24
3j	508	93500	508	533	0.55	25
3k	507	99500	507	528	0.55	21

[a]Data were measured in a concentration of 3.0 × 10^{-6} M. [b]Data were measured in a concentration of 1.0 × 10^{-6} M. [c]The fluorescence quantum yields (Φ) were calculated using Fluorescein in 0.1N NaOH solution (Φ = 0.91, excitation = 488 nm).

Table S2 Photophysical properties of BODIPY dyes 1, 3a-k in EtOH at room temperature

dyes	λabs (nm)[a]	ε ($M^{-1}.cm^{-1}$)	λex (nm)[b]	λem (nm)[b]	$\phi_F^{a,c}$	Stokes shift (nm)
1	495	72400	493	505	1.04	12
3a	505	85800	505	531	0.76	26
3b	507	79000	506	539	0.64	33
3c	507	36100	505	531	0.64	26
3e	507	76500	506	551	0.27	45
3f	503	72000	504	524	0.74	20
3g	505	85700	505	527	0.71	22
3h	505	75100	504	529	0.66	25
3i	505	69900	503	530	0.55	27
3j	505	79400	505	529	0.65	24
3k	505	88600	503	525	0.64	22

[a]Data were measured in a concentration of 3.0 × 10^{-6} M. [b]Data were measured in a concentration of 1.0 × 10^{-6} M. [c]The fluorescence quantum yields (Φ) were calculated using Fluorescein in 0.1N NaOH solution (Φ = 0.91, excitation = 488 nm).

Table S3 Photophysical properties of BODIPY dyes 4a-k, 5 and 7 in CH_2Cl_2 at room temperature

dyes	λabs (nm)[a]	ε ($M^{-1}.cm^{-1}$)	λex (nm)[b]	λem (nm)[b]	$\phi_F^{a,c}$	Stokes shift (nm)
4a	521	98800	521	552	0.56	31
4b	523	60800	523	561	0.41	38
4c	523	89900	523	554	0.50	31
4e	527	57500	526	574	0.37	48
4f	518	108200	517	546	0.43	29
4g	522	101400	522	552	0.51	30

4h	520	84400	520	552	0.54	32
4i	520	69900	520	554	0.57	34
4j	522	118400	521	555	0.48	34
4k	520	87400	519	550	0.42	31
5	523	74700	522	575	0.61	53
7	514	66200	515	537	0.74	22

[a]Data were measured in a concentration of 3.0 × 10^{-6} M. [b]Data were measured in a concentration of 1.0 × 10^{-6} M. [c]The fluorescence quantum yields (Φ) were calculated using and Rhodamine 6G in anhydrous ethanol (Φ = 0.95, excitation = 530nm).

Table S4 Photophysical properties of BODIPY dyes 4a-k in CH_3CN at room temperature

dyes	λabs (nm)[a]	ε ($M^{-1}.cm^{-1}$)	λex (nm)[b]	λem (nm)[b]	ϕ_F[a,c]	Stokes shift (nm)
4a	517	87500	515	550	0.53	35
4b	519	51600	518	557	0.34	39
4c	518	78900	517	552	0.52	35
4e	520	49100	519	570	0.31	51
4f	513	90600	512	541	0.25	29
4e	517	88200	516	549	0.49	33
4h	515	68600	513	548	0.55	35
4i	515	63000	515	550	0.56	35
4j	515	58800	515	549	0.86	34
4k	514	72100	513	546	0.52	33

[a]Data were measured in a concentration of 3.0 × 10^{-6} M. [b]Data were measured in a concentration of 1.0 × 10^{-6} M. [c]The fluorescence quantum yields (Φ) were calculated using Rhodamine 6G in anhydrous ethanol (Φ = 0.95, excitation = 530 nm).

Table S5 Photophysical properties of BODIPY dyes 4a-k, 5 and 7 in EtOH at room temperature

dyes	λabs (nm)[a]	ε ($M^{-1}.cm^{-1}$)	λex (nm)[b]	λem (nm)[b]	ϕ_F[a,c]	Stokes shift (nm)
4a	518	89300	518	552	0.54	34
4b	520	51400	520	557	0.36	37
4c	520	84600	520	553	0.49	33
4e	523	54300	522	574	0.33	52
4f	515	38300	515	541	0.42	26
4e	518	58500	517	550	0.47	33
4h	517	69300	516	549	0.56	33
4i	518	62400	517	551	0.57	34

4j	518	98500	519	552	0.53	33
4k	517	72600	515	547	0.49	32
5	520	54700	519	573	0.53	54
7	511	66400	511	533	0.73	22

[a]Data were measured in a concentration of 3.0 × 10^{-6} M. [b]Data were measured in a concentration of 1.0 × 10^{-6} M. [c]The fluorescence quantum yields (Φ) were calculated using Rhodamine 6G in anhydrous ethanol (Φ = 0.95, excitation = 530 nm).

Table S6 Photophysical properties of BODIPY dyes 9a-f in CH_2Cl_2 at room temperature

dyes	λabs (nm)[a]	ε ($M^{-1}.cm^{-1}$)	λex (nm)[b]	λem (nm)[b]	ϕ_F[a,c]	Stokes shift (nm)
9a	527	60200	524	606	0.017	82
9b	528	44300	527	615	0.016	88
9c	523	57600	516	599	0.022	83
9d	527	83200	526	601	0.021	75
9e	528	55300	527	602	0.027	75
9f	515	48900	539	598	0.11	59

[a]Data were measured in a concentration of 3.0 × 10^{-6} M. [b]Data were measured in a concentration of 1.0 × 10^{-6} M. [c]The fluorescence quantum yields (Φ) were calculated using Rhodamine 6G in anhydrous ethanol (Φ = 0.95, excitation = 530 nm).

Table S7 Photophysical properties of BODIPY dyes 9a-f in EtOH at room temperature

dyes	λabs (nm)[a]	ε ($M^{-1}.cm^{-1}$)	λex (nm)[b]	λem (nm)[b]	ϕ_F[a,c]	Stokes shift (nm)
9a	523	50300	521	606	0.011	85
9b	525	38200	523	618	0.010	95
9c	523	52200	512	598	0.013	86
9d	523	70300	522	603	0.014	81
9e	525	47100	522	603	0.016	81
9f	513	45200	539	596	0.096	57

[a]Data were measured in a concentration of 3.0 × 10^{-6} M. [b]Data were measured in a concentration of 1.0 × 10^{-6} M. [c]The fluorescence quantum yields (Φ) were calculated using Rhodamine 6G in anhydrous ethanol (Φ = 0.95, excitation = 530 nm).

Figure S1: Fluorescent emission spectra of dye 3a in different solvents (CH_2Cl_2, CH_3CN and EtOH, 5.0 × 10^{-6} M, excited at λ_{max}, respectively)

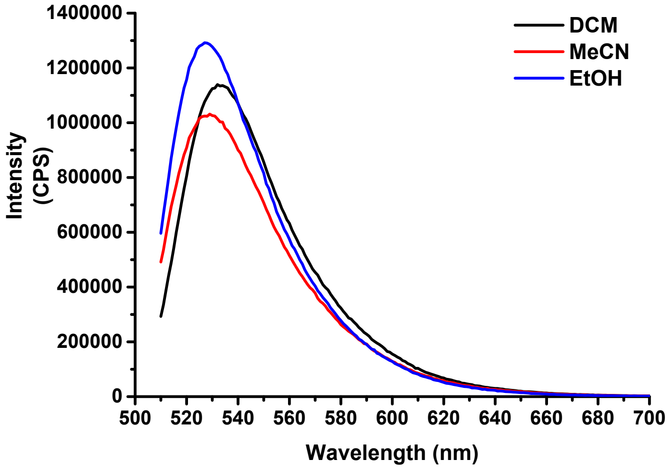

Figure S2: Absorbance spectra of dye 3a in different solvents (CH_2Cl_2, CH_3CN and EtOH, 1.0 × 10^{-6} M)

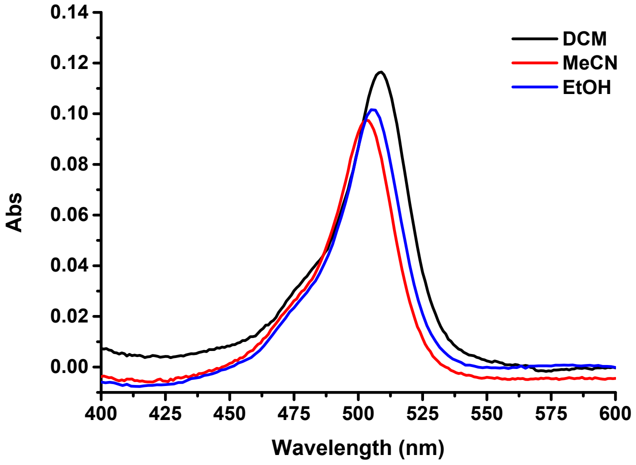

Figure S3: Fluorescent Emission spectra of 3a, 3e, 3g, and 4l in CH_2Cl_2 (5.0 × 10^{-6} M, excited at their λ_{max}, respectively)

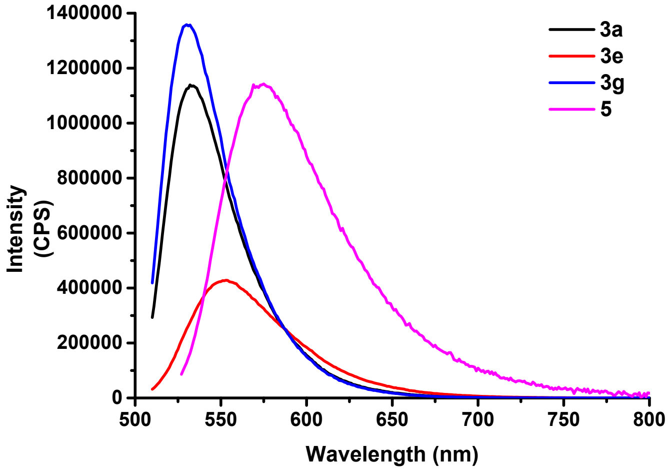

Figure S4: Absorbance spectra of 3a, 3e, 3g and 4l in CH_2Cl_2 (1.0 × 10^{-6} M)

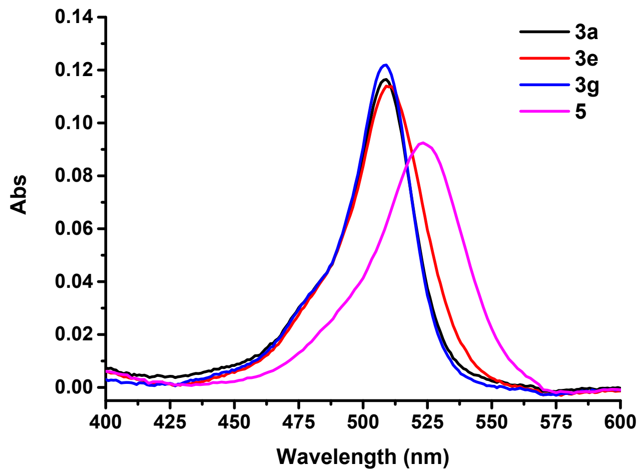

Figure S5: Fluorescent Emission spectra of 6a-e in EtOH (5.0×10^{-6} M, excited at their λ_{max}, respectively)

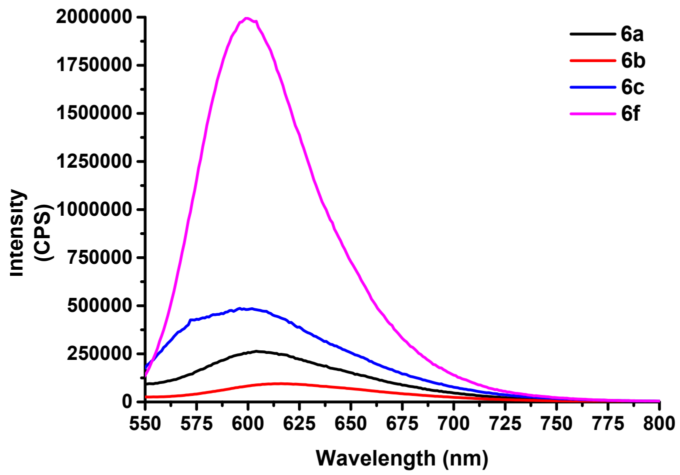

Figure S6: Absorbance spectra of 6a-e in EtOH (1.0×10^{-6} M)

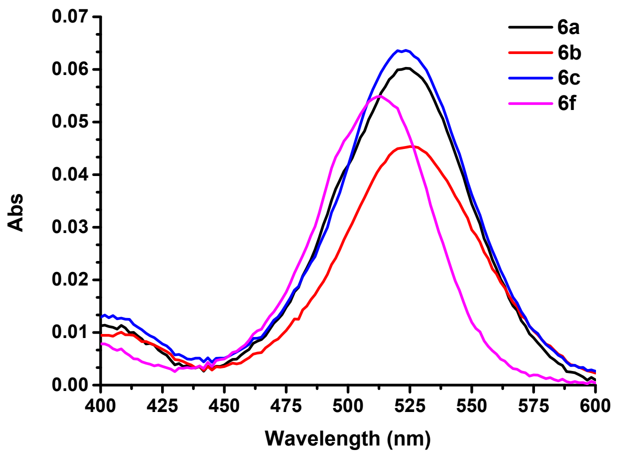

Figure S7. Morphological changes of MDA-MB-231 cells after laser irradiation in presence of solvent control (Column 1), compound 6a (Column 2 and 3), 6b (Column 4 and 5) and 6f (Column 6 and 7), respectively. Three compounds (**6a**, **6b** and **6f**) were added to MDA-MB-231 cells with two different concentrations (2.5 μM and 5.0 μM) respectively. During the period of laser irradiation for 10 min, fluorescent images (showing the presence of the compound in cells) and brightfield images were acquired at 0min (Row 1 and 2), 5min (Row 3 and 4) and 10 min (Row 5 and 6). Blue boxes, laser irradiated regions; Scale bar: 50 μm.

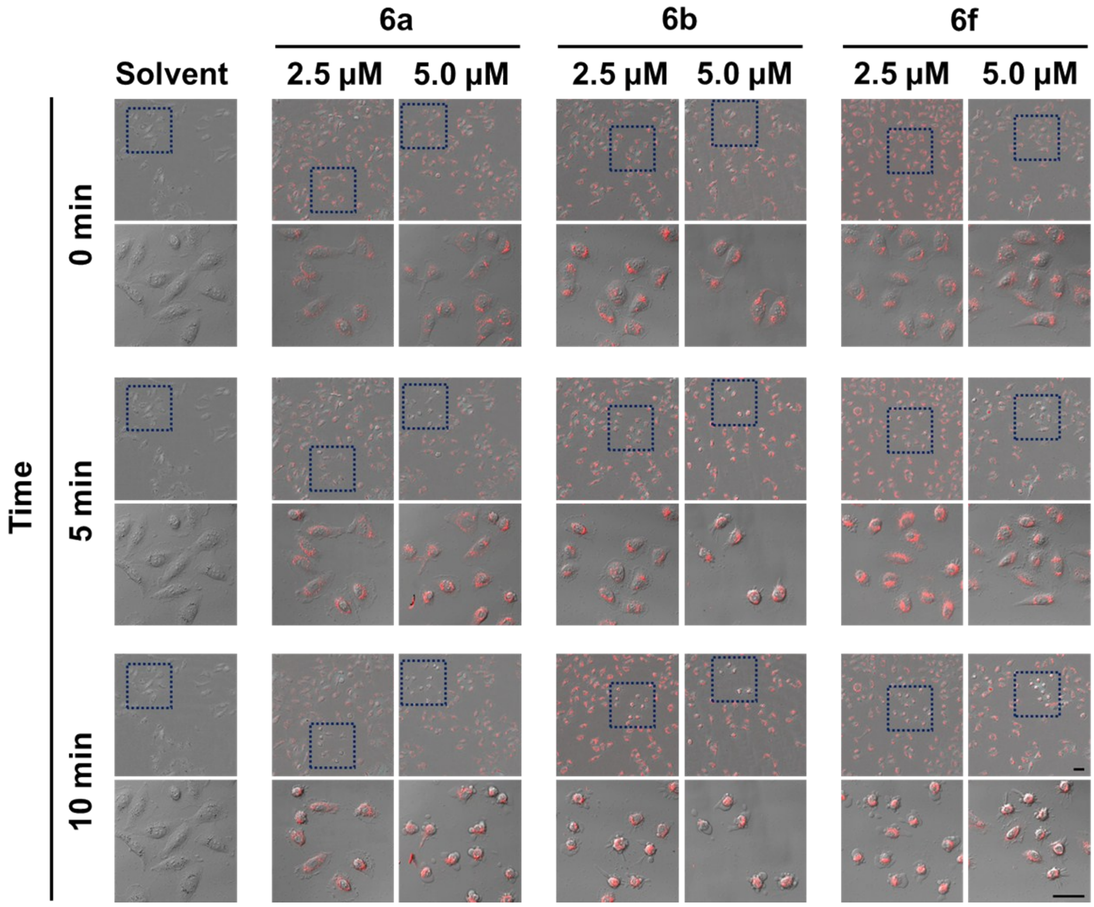

Reference:

1. Toh, Q. Y.; McNally, A.; Vera, S.; Erdmann, N.; Gaunt, M. J., Organocatalytic C–H Bond Arylation of Aldehydes to Bis-heteroaryl Ketones. *Journal of the American Chemical Society* **2013,** *135* (10), 3772-3775.

2. Bigot, A.; Williamson, A. E.; Gaunt, M. J., Enantioselective α-Arylation of N-Acyloxazolidinones with Copper(II)-bisoxazoline Catalysts and Diaryliodonium Salts. *Journal of the American Chemical Society* **2011,** *133* (35), 13778-13781.

4. ^{1}H and ^{13}C NMR Spectra

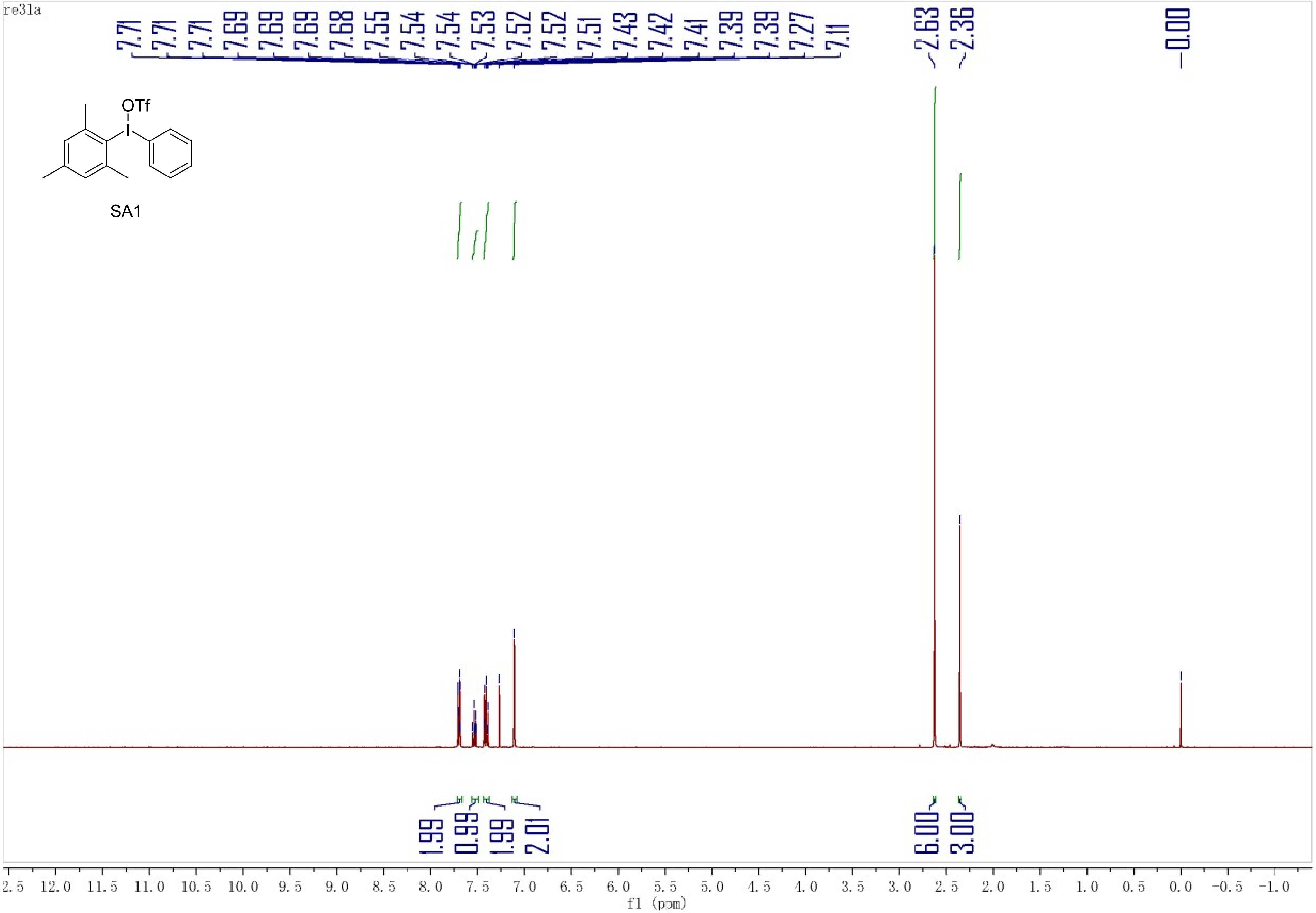
re31a
7.71
7.71
7.71
7.69
7.69
7.69
7.68
7.55
7.54
7.54
7.53
7.52
7.52
7.51
7.43
7.42
7.41
7.39
7.39
7.27
7.11
2.63
2.36
0.00
OTf
I
SA1
1.99
0.99
1.99
2.01
6.00
3.00
f1 (ppm)

SA2

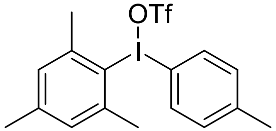

SA2

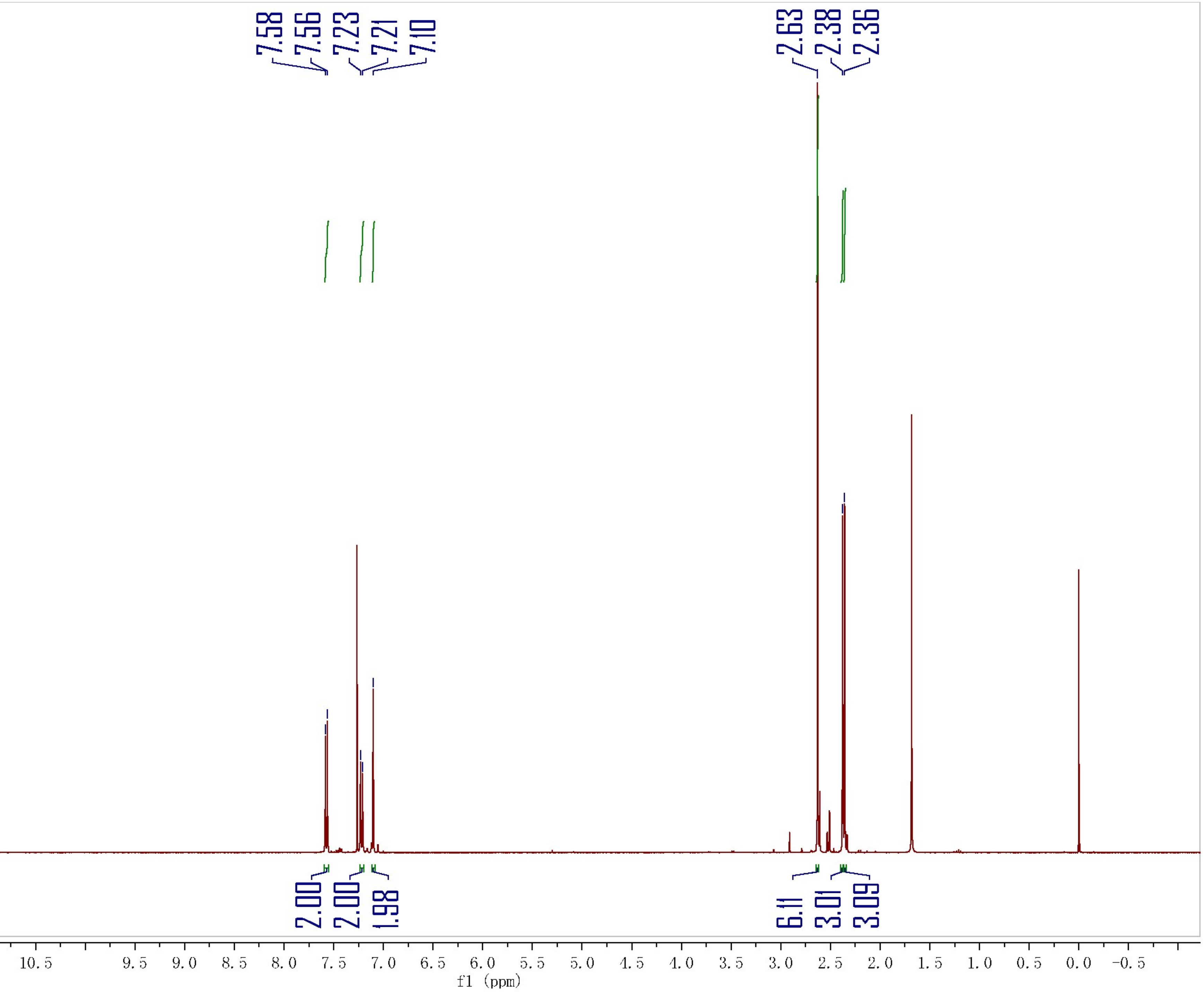
7.58
7.56
7.23
7.21
7.10
2.63
2.38
2.36
2.00
2.00
1.98
6.11
3.01
3.09
12.5
11.5
10.5
9.5
9.0
8.5
8.0
7.5
7.0
6.5
6.0
5.5
5.0
4.5
4.0
3.5
3.0
2.5
2.0
1.5
1.0
0.5
0.0
-0.5
f1 (ppm)

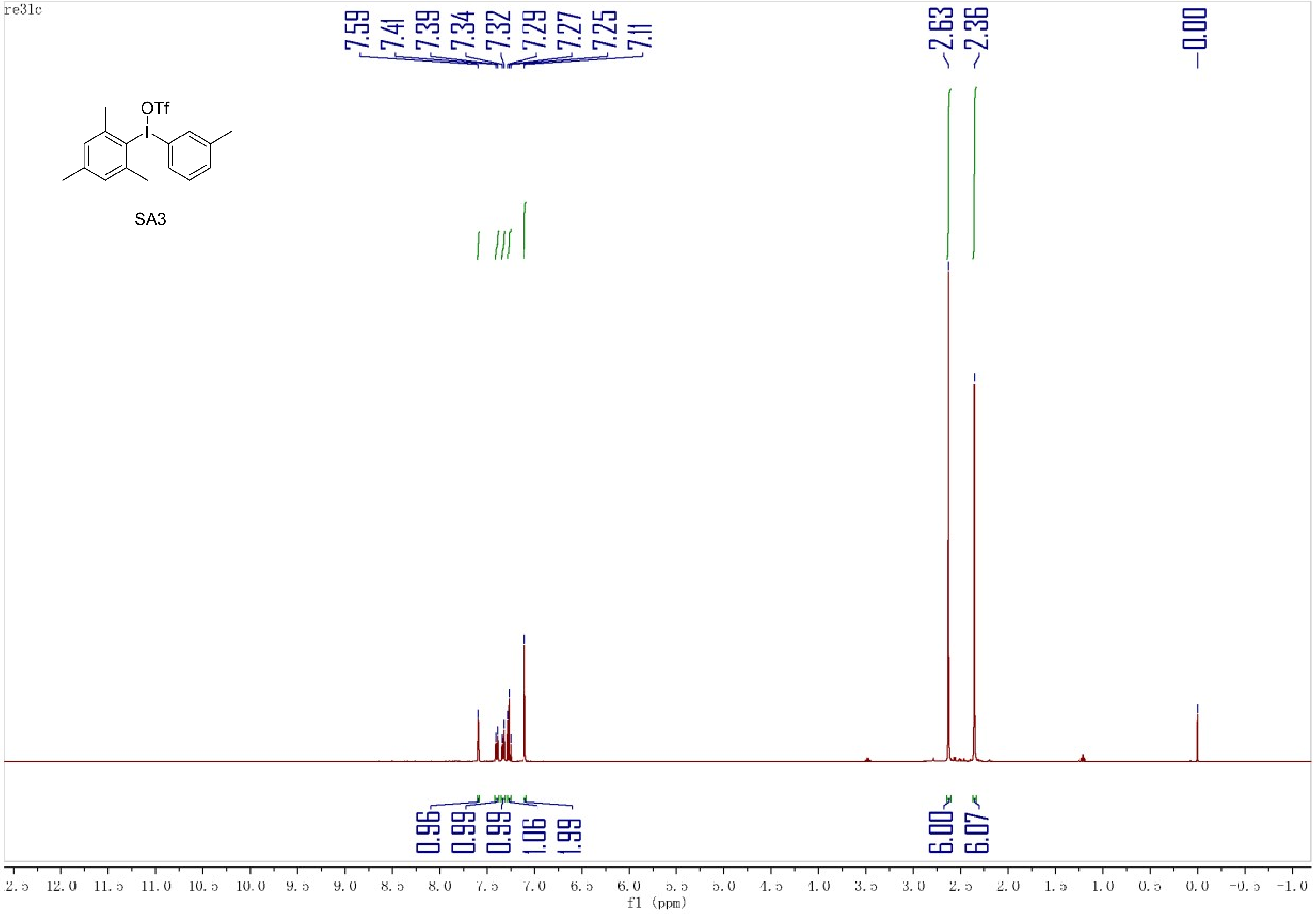
re31c
OTf
I
SA3
7.59
7.41
7.39
7.34
7.32
7.29
7.27
7.25
7.11
2.63
2.36
0.00
0.96
0.99
0.99
1.06
1.99
6.00
6.07
12.5 12.0 11.5 11.0 10.5 10.0 9.5 9.0 8.5 8.0 7.5 7.0 6.5 6.0 5.5 5.0 4.5 4.0 3.5 3.0 2.5 2.0 1.5 1.0 0.5 0.0 -0.5 -1.0
f1 (ppm)

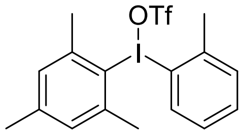
OTf
I

SA4

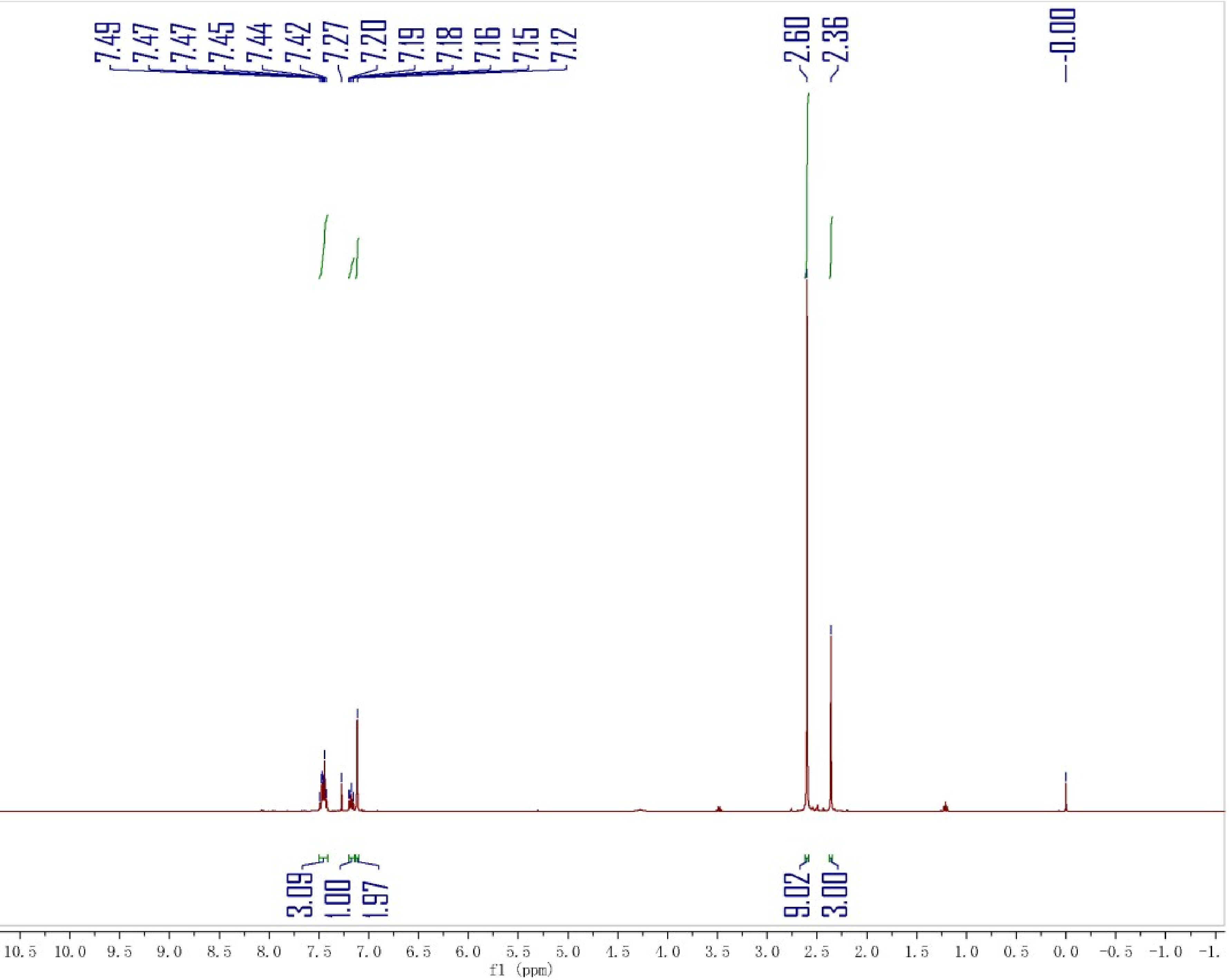
re31d
7.49
7.47
7.47
7.45
7.44
7.42
7.27
7.20
7.19
7.18
7.16
7.15
7.12
2.60
2.36
0.00
3.09
1.00
1.97
9.02
3.00
12.0
11.5
11.0
10.5
10.0
9.5
9.0
8.5
8.0
7.5
7.0
6.5
6.0
5.5
5.0
4.5
4.0
3.5
3.0
2.5
2.0
1.5
1.0
0.5
0.0
-0.5
-1.0
f1 (ppm)

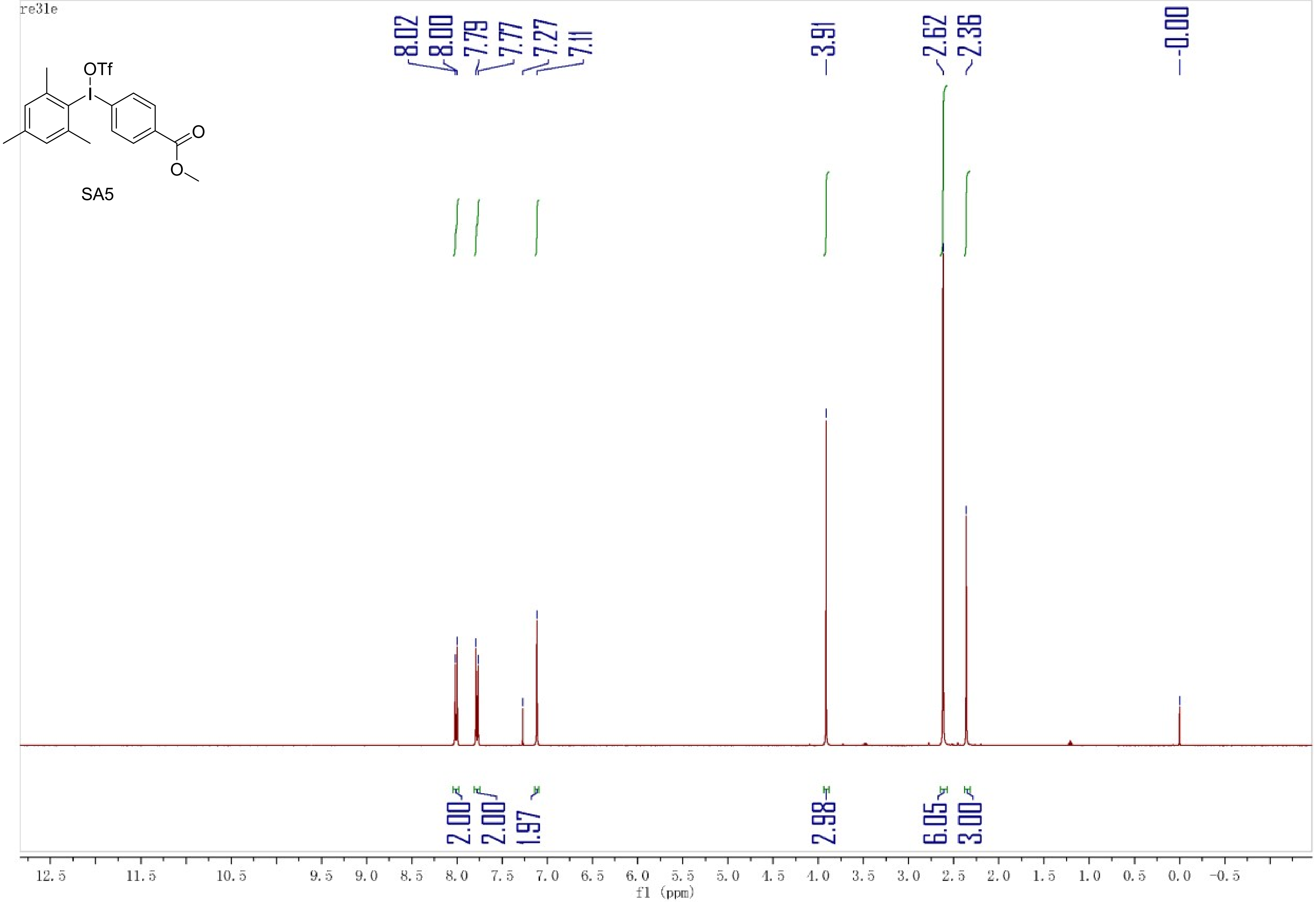

re31e
OTf
I
O
O
SA5
8.02
8.00
7.79
7.77
7.27
7.11
3.91
2.62
2.36
-0.00
2.00
2.00
1.97
2.98
6.05
3.00
12.5 11.5 10.5 9.5 9.0 8.5 8.0 7.5 7.0 6.5 6.0 5.5 5.0 4.5 4.0 3.5 3.0 2.5 2.0 1.5 1.0 0.5 0.0 -0.5
f1 (ppm)

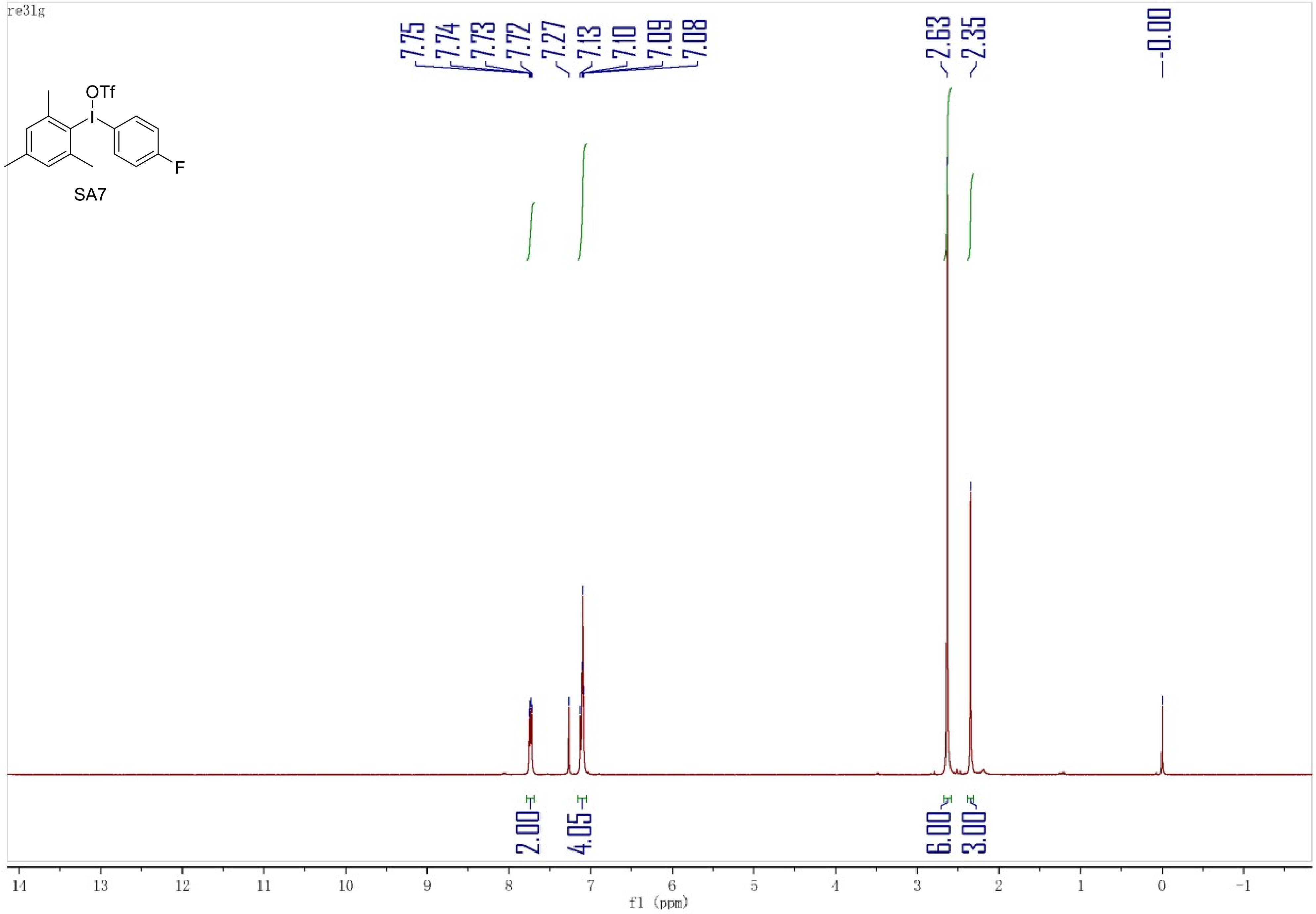

re31g
OTf
I
F
SA7
7.75
7.74
7.73
7.72
7.27
7.13
7.10
7.09
7.08
2.63
2.35
0.00
2.00
4.05
6.00
3.00
14
13
12
11
10
9
8
7
6
5
4
3
2
1
0
-1
f1 (ppm)

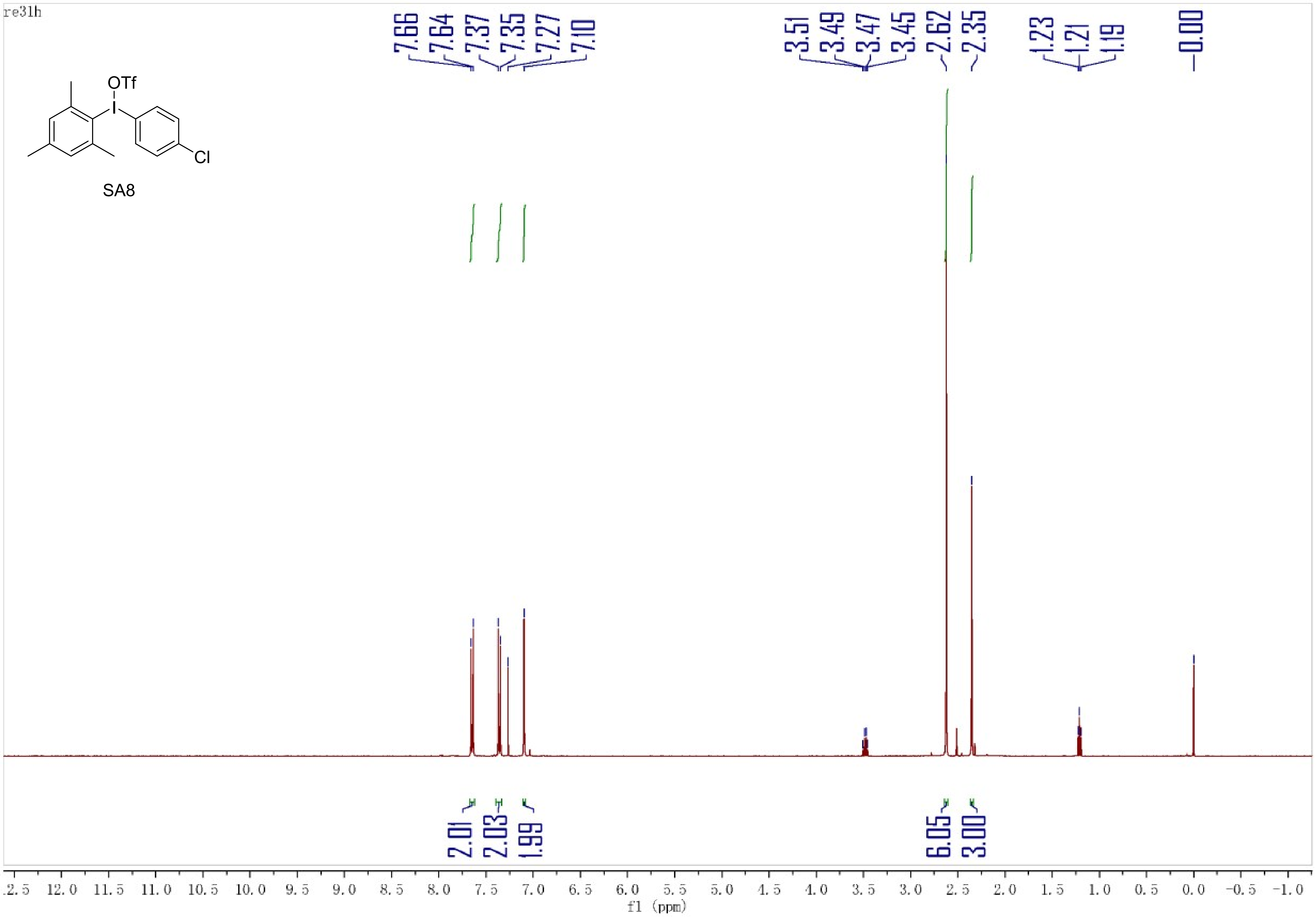
re31h
OTf
I
Cl
7.66
7.64
7.37
7.35
7.27
7.10
3.51
3.49
3.47
3.45
2.62
2.35
1.23
1.21
1.19
0.00
2.01
2.03
1.99
6.05
3.00
f1 (ppm)

SA8

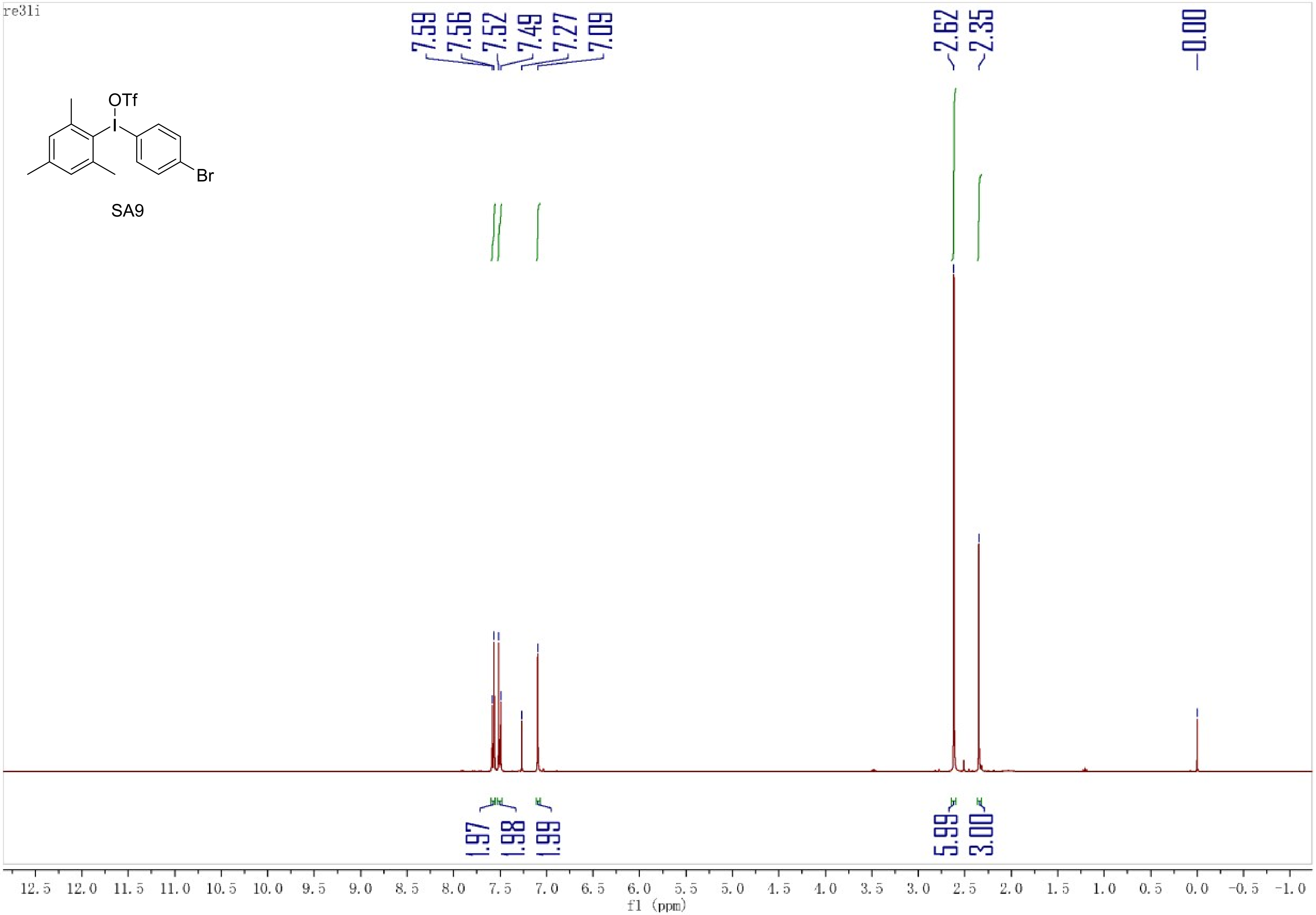
re31i
7.59
7.56
7.52
7.49
7.27
7.09
2.62
2.35
0.00
OTf
I
Br
SA9
1.97
1.98
1.99
5.99
3.00
12.5 12.0 11.5 11.0 10.5 10.0 9.5 9.0 8.5 8.0 7.5 7.0 6.5 6.0 5.5 5.0 4.5 4.0 3.5 3.0 2.5 2.0 1.5 1.0 0.5 0.0 -0.5 -1.0
f1 (ppm)

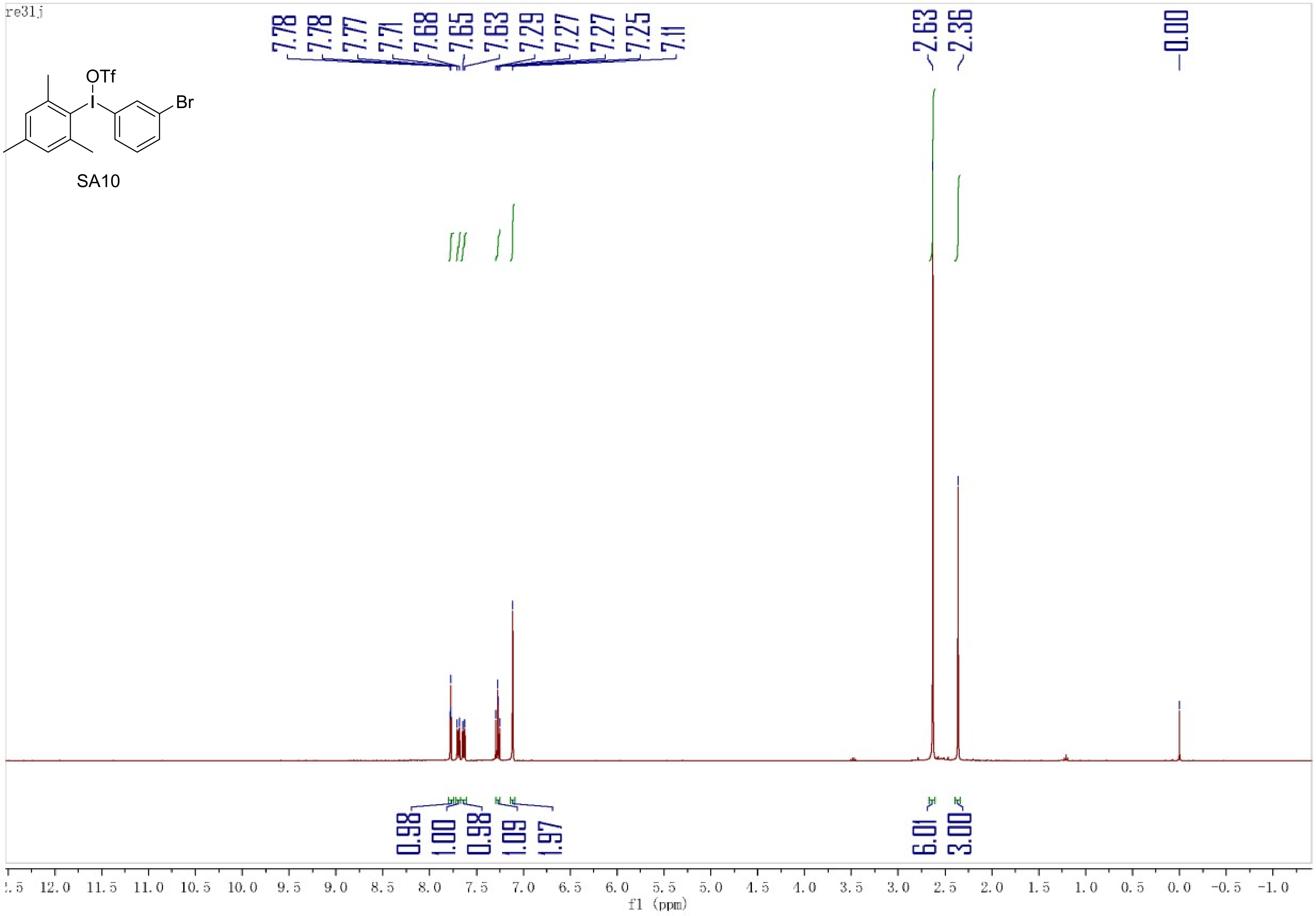
re31j
OTf
I
Br
SA10
7.78 7.78 7.77 7.71 7.68 7.65 7.63 7.29 7.27 7.27 7.25 7.11
2.63 2.36
0.00
0.98 1.00 0.98 1.09 1.97
6.01 3.00
f1 (ppm)

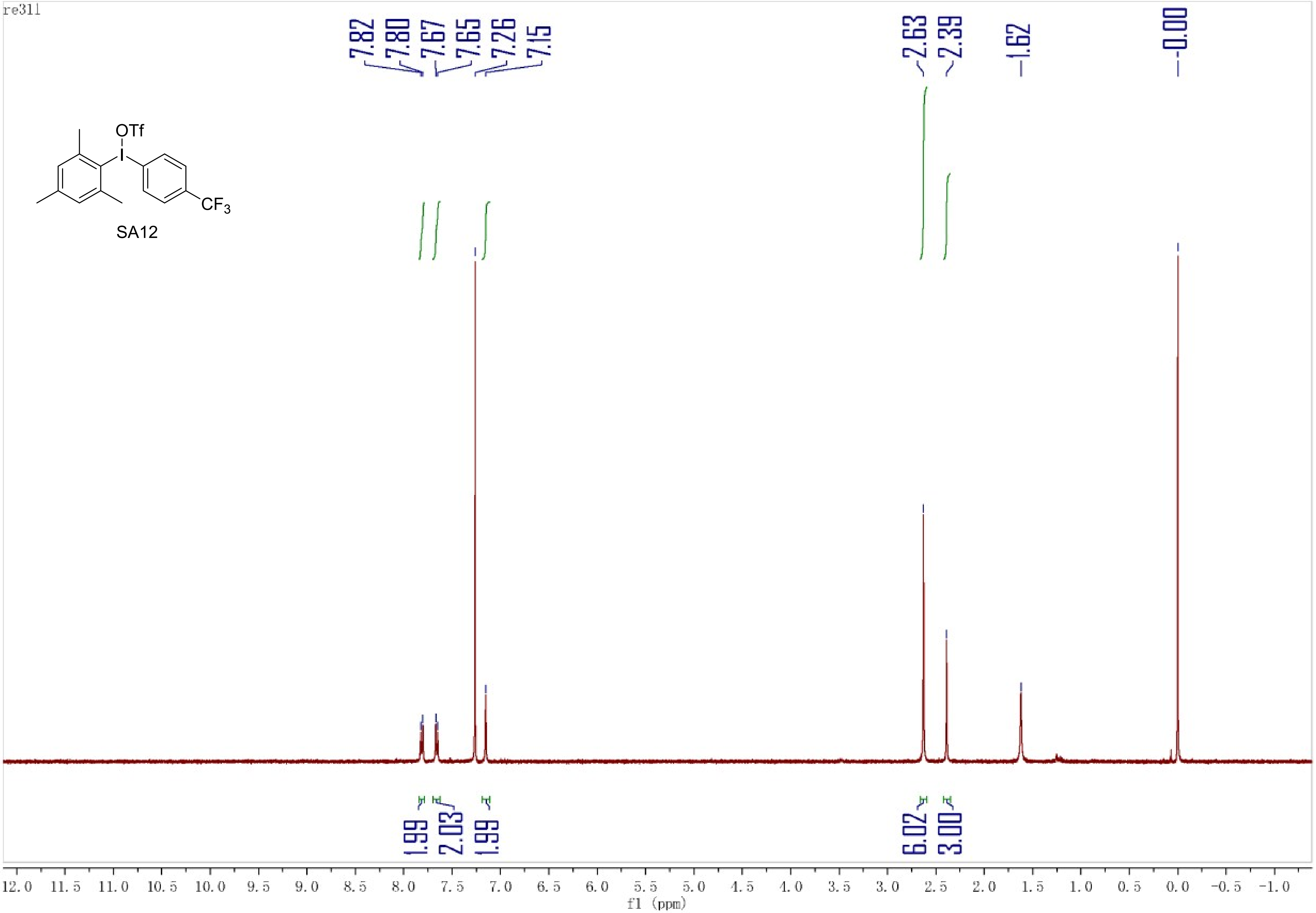

re311
7.82
7.80
7.67
7.65
7.26
7.15
2.63
2.39
1.62
0.00
OTf
I
CF3
SA12
1.99
2.03
1.99
6.02
3.00
12.0
11.5
11.0
10.5
10.0
9.5
9.0
8.5
8.0
7.5
7.0
6.5
6.0
5.5
5.0
4.5
4.0
3.5
3.0
2.5
2.0
1.5
1.0
0.5
0.0
-0.5
-1.0
f1 (ppm)

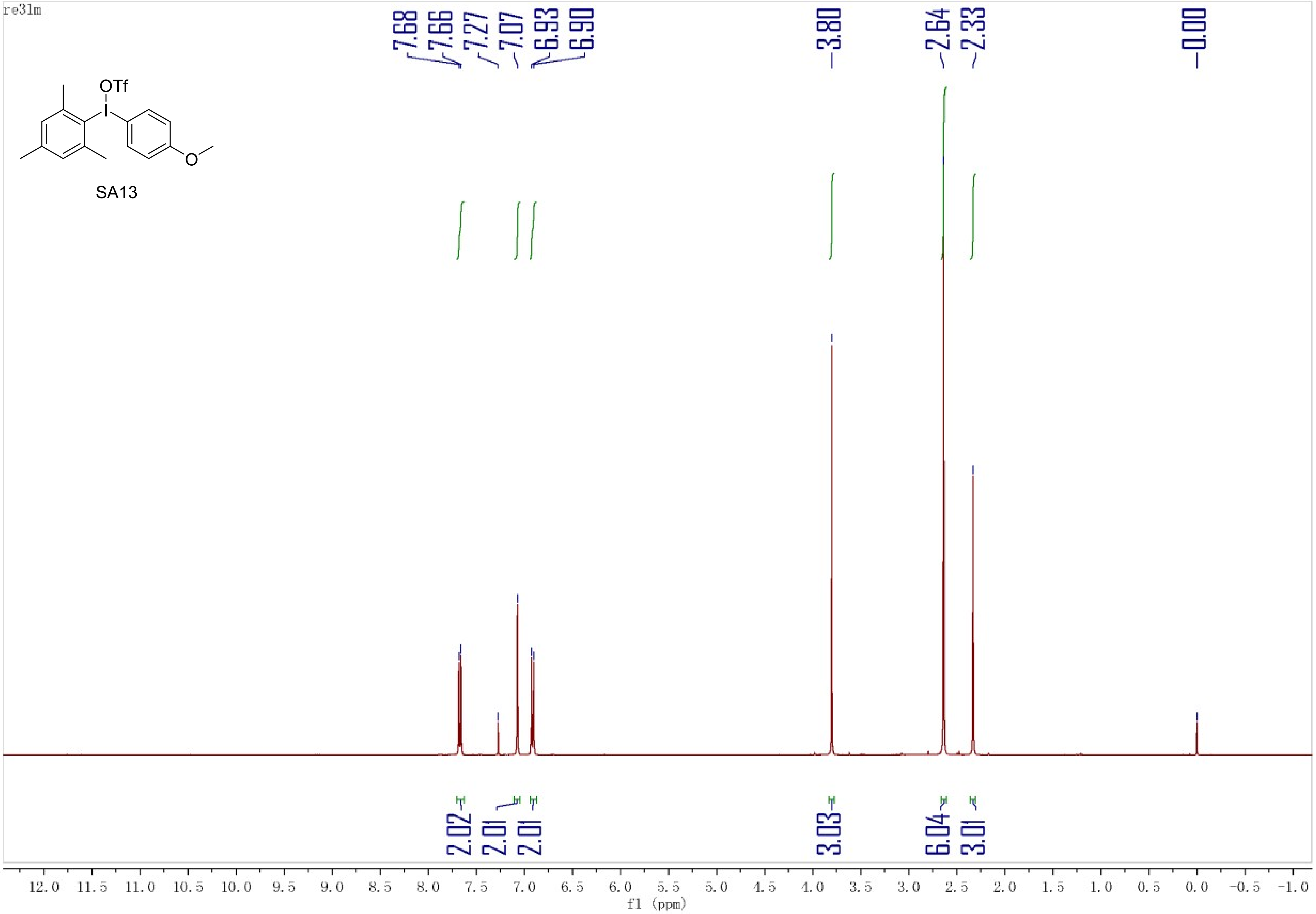
re3lm
7.68
7.66
7.27
7.07
6.93
6.90
3.80
2.64
2.33
0.00
OTf
I
O
SA13
2.02
2.01
2.01
3.03
6.04
3.01
12.0 11.5 11.0 10.5 10.0 9.5 9.0 8.5 8.0 7.5 7.0 6.5 6.0 5.5 5.0 4.5 4.0 3.5 3.0 2.5 2.0 1.5 1.0 0.5 0.0 -0.5 -1.0
f1 (ppm)

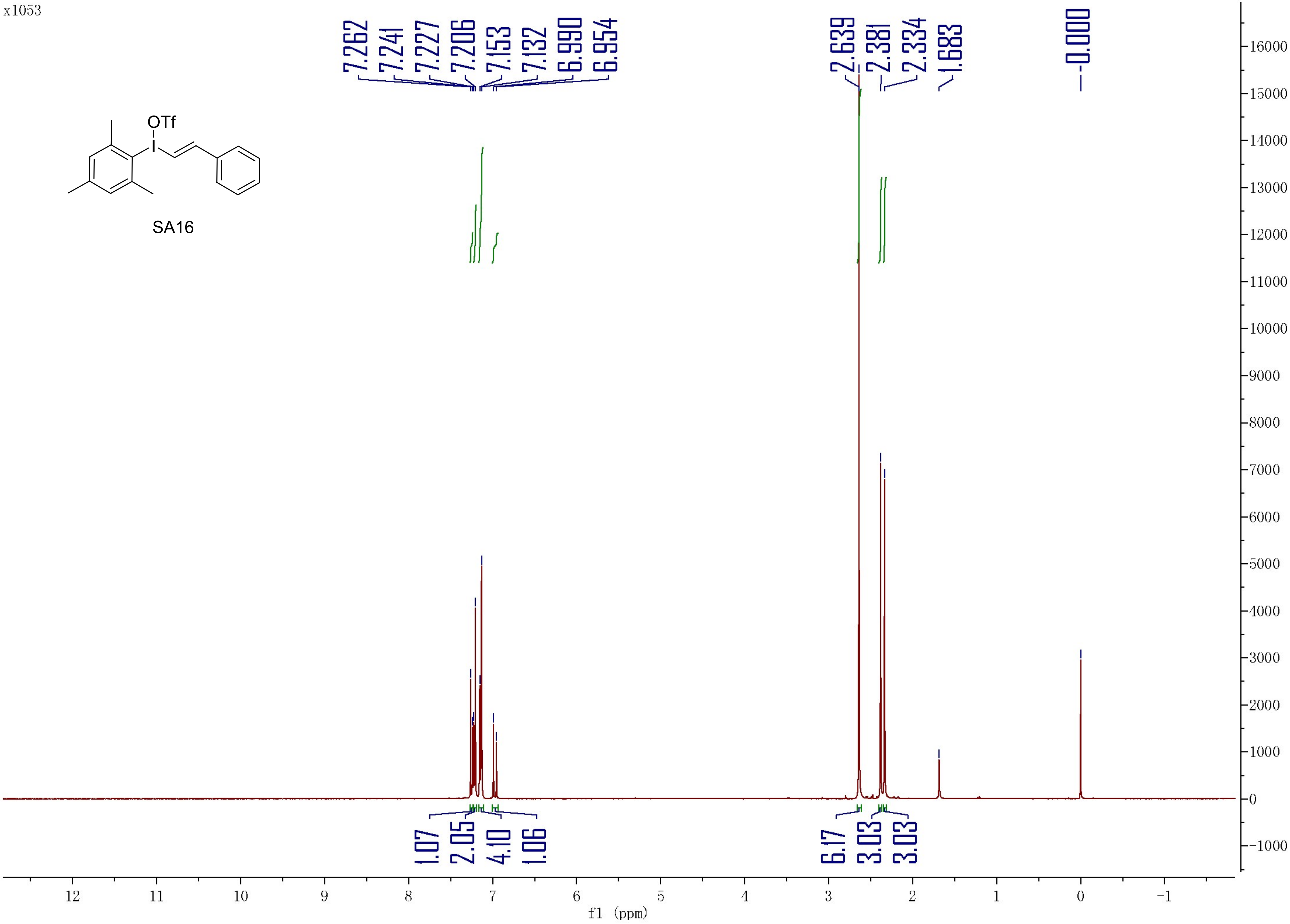

OTf
SA16
x1053
7.262
7.241
7.227
7.206
7.153
7.132
6.990
6.954
2.639
2.381
2.334
1.683
0.000
1.07
2.05
4.10
1.06
6.17
3.03
3.03
f1 (ppm)

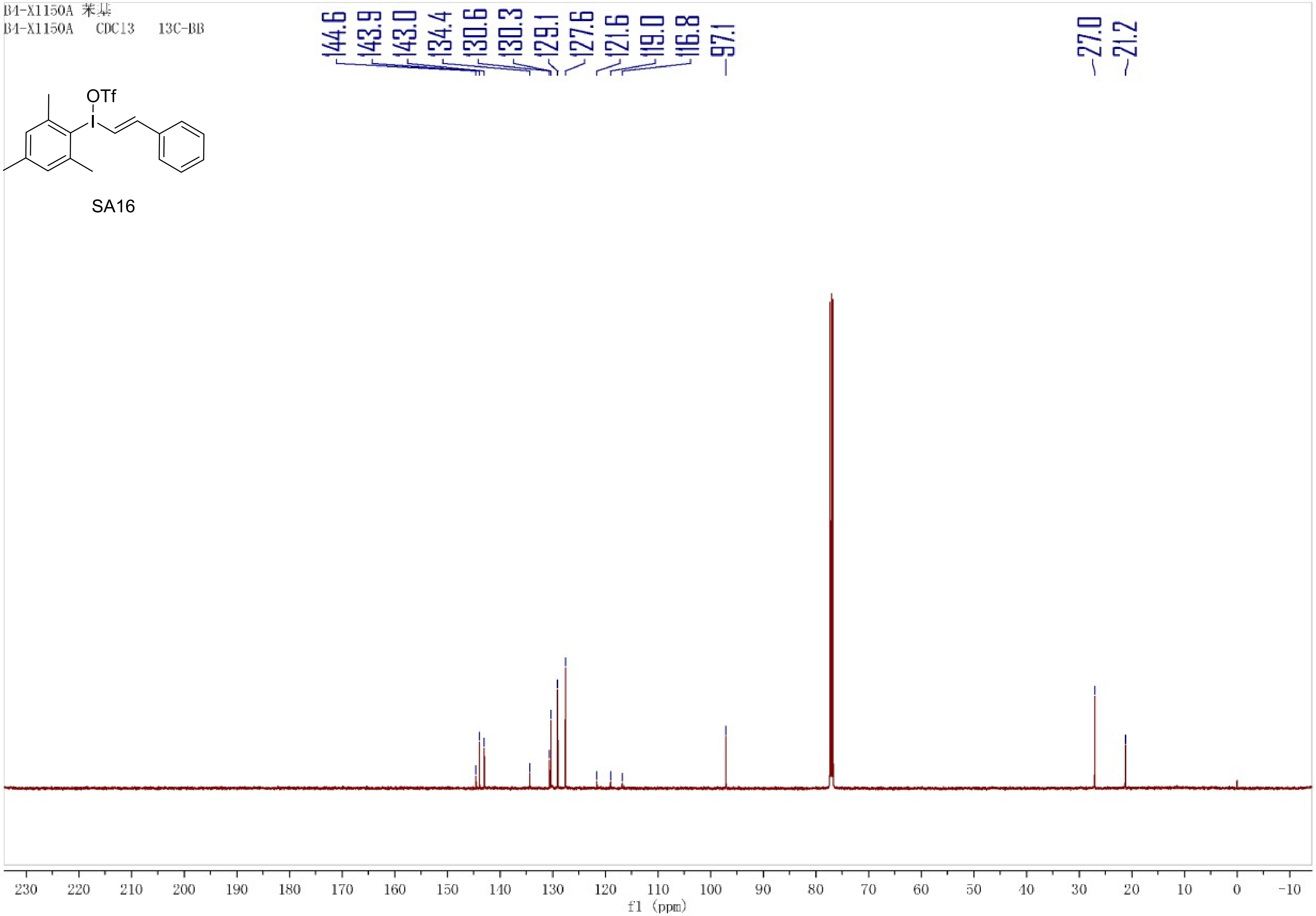
B4-X1150A 苯基
B4-X1150A CDCl3 13C-BB
144.6
143.9
143.0
134.4
130.6
130.3
129.1
127.6
121.6
119.0
116.8
97.1
27.0
21.2
OTf
I
SA16
230 220 210 200 190 180 170 160 150 140 130 120 110 100 90 80 70 60 50 40 30 20 10 0 -10
f1 (ppm)

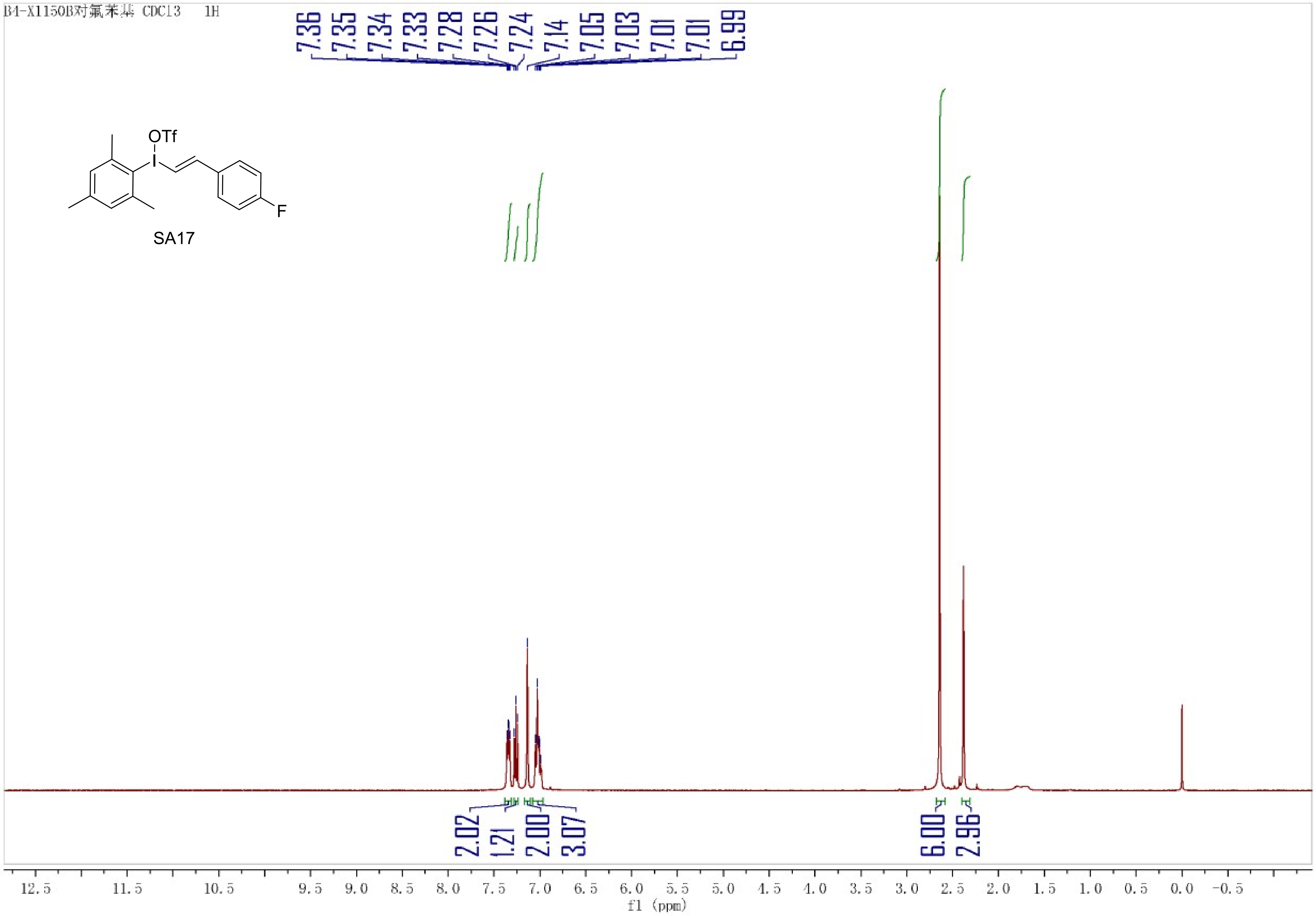
B4-X1150B对氟苯基 CDCl3 1H
7.36 7.35 7.34 7.33 7.28 7.26 7.24 7.14 7.05 7.03 7.01 7.01 6.99
OTf
I
F
SA17
2.02 1.21 2.00 3.07
6.00 2.96
12.5 11.5 10.5 9.5 9.0 8.5 8.0 7.5 7.0 6.5 6.0 5.5 5.0 4.5 4.0 3.5 3.0 2.5 2.0 1.5 1.0 0.5 0.0 -0.5
f1 (ppm)

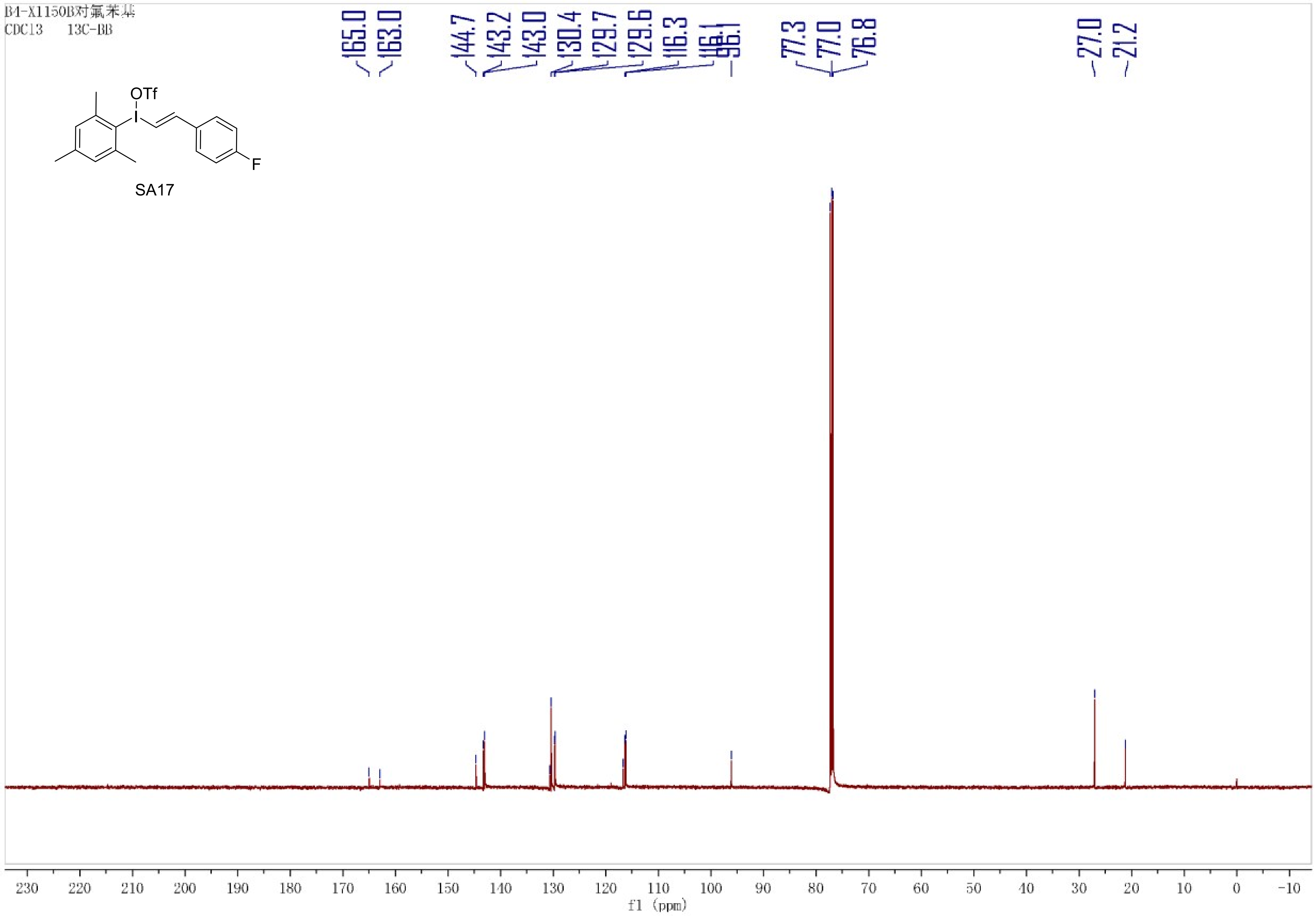

B4-X1150B对氟苯基
CDCl3 13C-BB
OTf
I
F
SA17
165.0
163.0
144.7
143.2
143.0
130.4
129.7
129.6
116.3
116.1
96.1
77.3
77.0
76.8
27.0
21.2
230 220 210 200 190 180 170 160 150 140 130 120 110 100 90 80 70 60 50 40 30 20 10 0 -10
f1 (ppm)

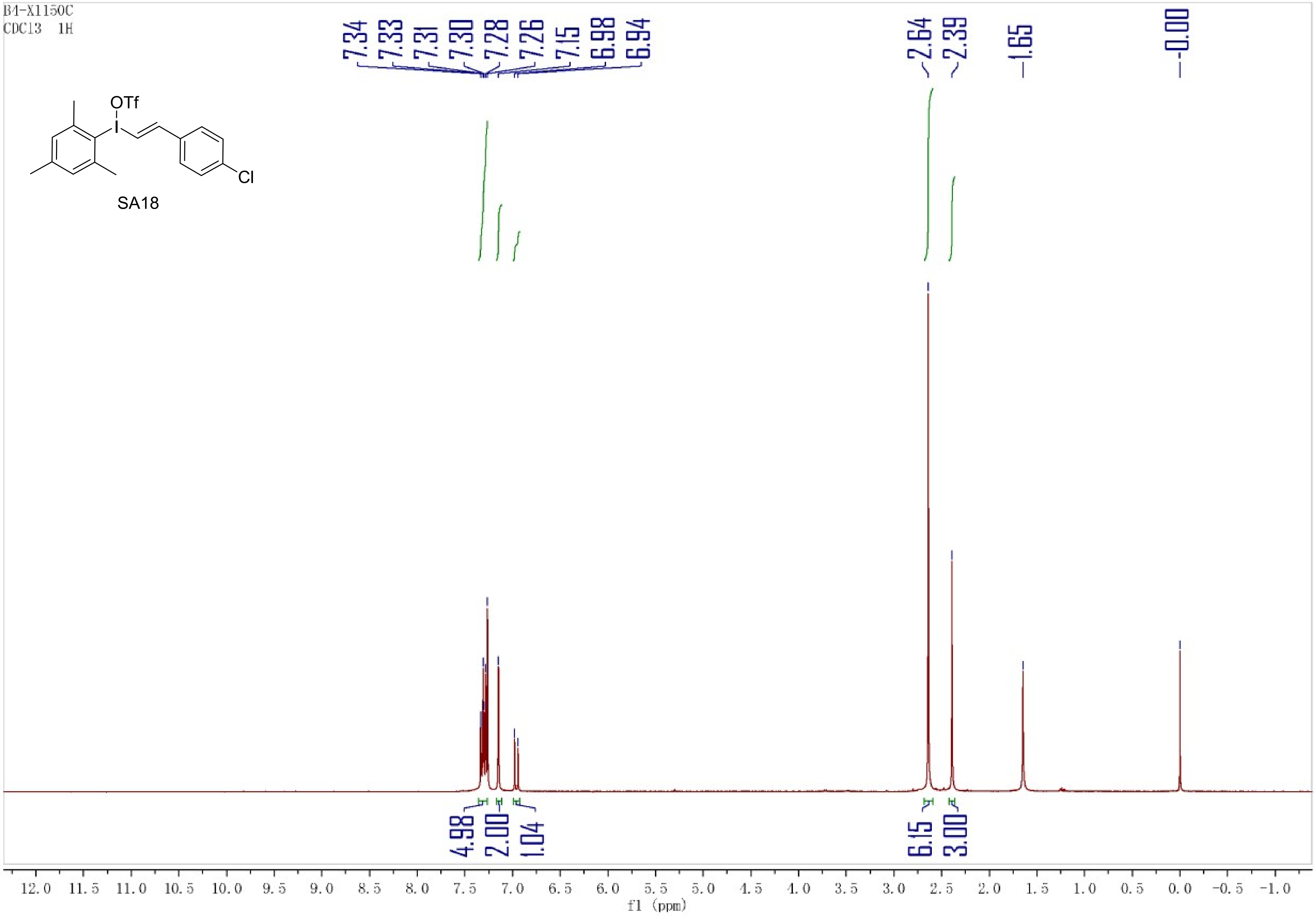
B4-X1150C
CDCl3 1H
OTf
I
Cl
SA18
7.34
7.33
7.31
7.30
7.28
7.26
7.15
6.98
6.94
2.64
2.39
1.65
0.00
4.98
2.00
1.04
6.15
3.00
12.0 11.5 11.0 10.5 10.0 9.5 9.0 8.5 8.0 7.5 7.0 6.5 6.0 5.5 5.0 4.5 4.0 3.5 3.0 2.5 2.0 1.5 1.0 0.5 0.0 -0.5 -1.0
f1 (ppm)

B4-X1150C
CDCl3 13C-BB

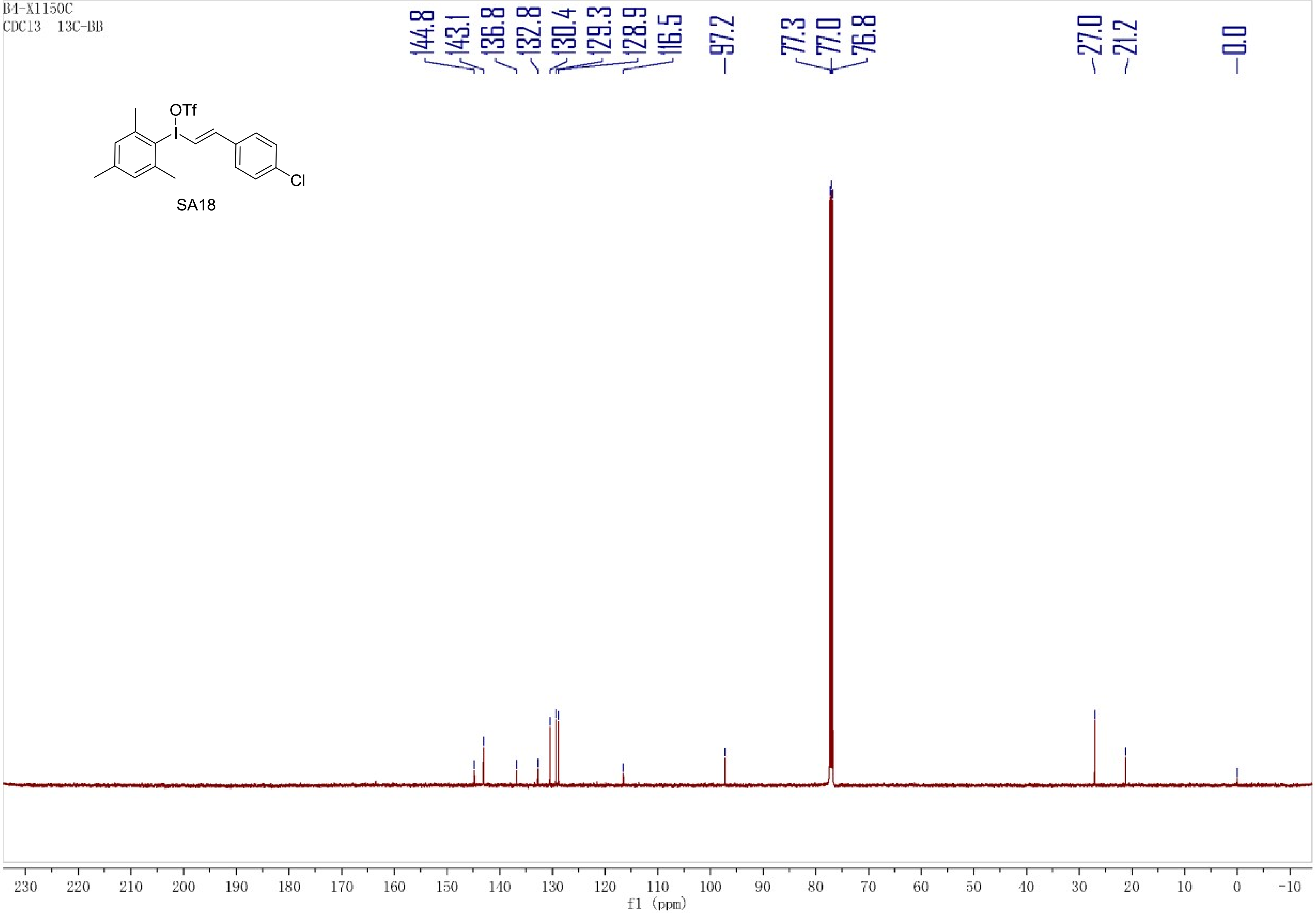

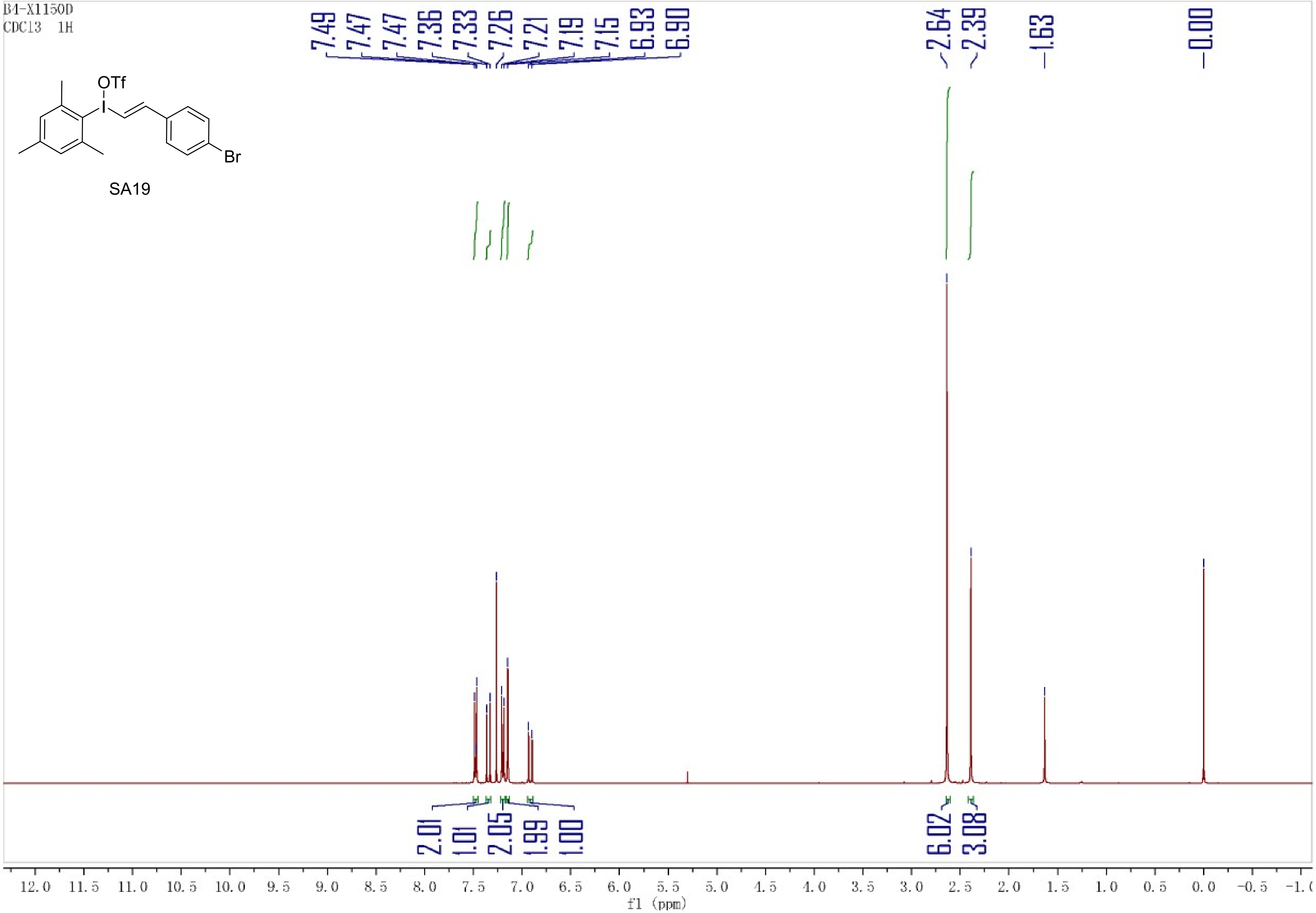
B4-X1150D
CDCl3 1H
OTf
I
Br
SA19
7.49 7.47 7.47 7.36 7.33 7.26 7.21 7.19 7.15 6.93 6.90
2.64 2.39
1.63
0.00
2.01 1.01 2.05 1.99 1.00
6.02 3.08
12.0 11.5 11.0 10.5 10.0 9.5 9.0 8.5 8.0 7.5 7.0 6.5 6.0 5.5 5.0 4.5 4.0 3.5 3.0 2.5 2.0 1.5 1.0 0.5 0.0 -0.5
f1 (ppm)

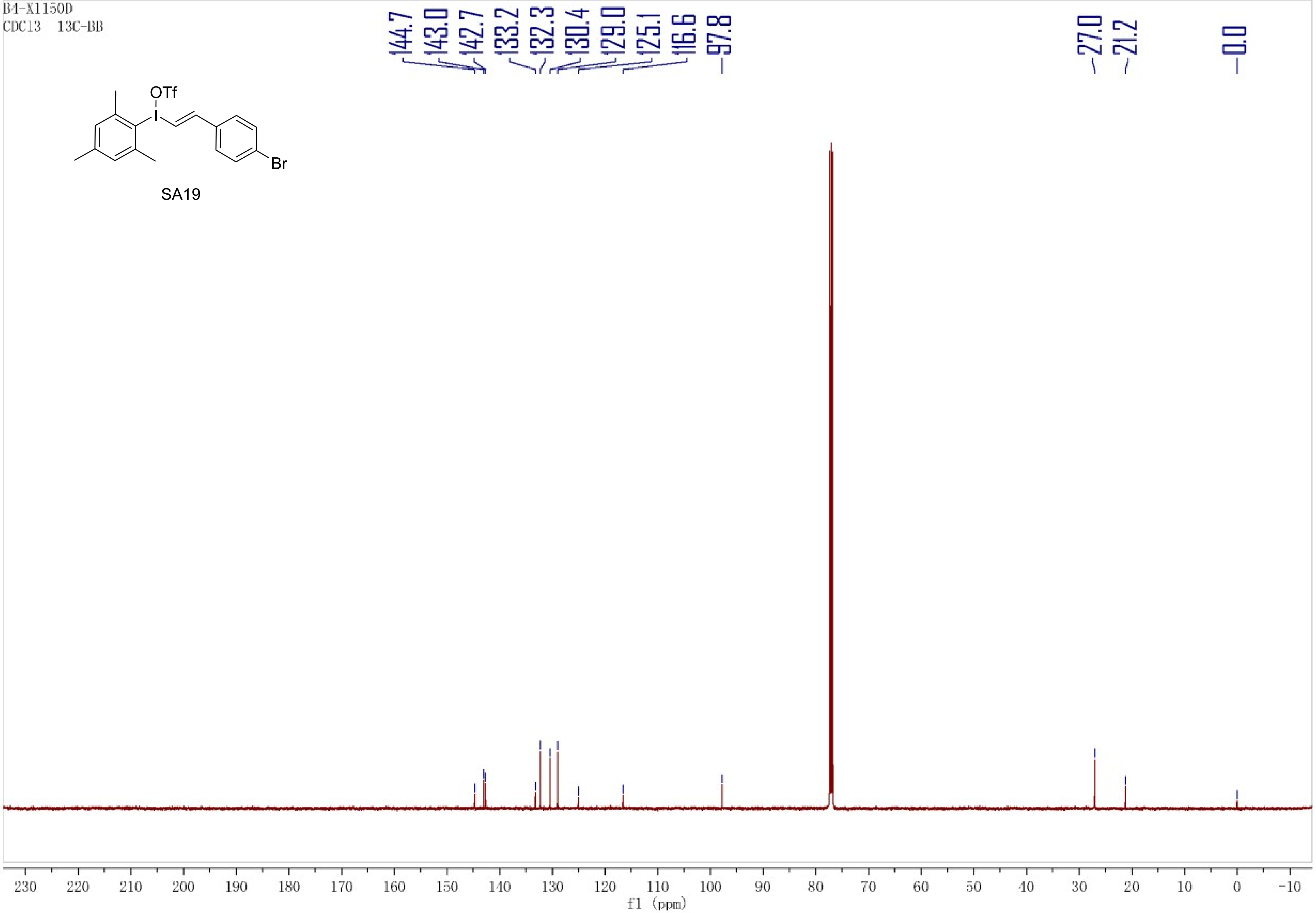
B4-X1150D
CDCl3 13C-BB
144.7
143.0
142.7
133.2
132.3
130.4
129.0
125.1
116.6
97.8
27.0
21.2
0.0
OTf
I
Br
SA19
230 220 210 200 190 180 170 160 150 140 130 120 110 100 90 80 70 60 50 40 30 20 10 0 -10
f1 (ppm)

B4-X1150E
CDCl3 1H

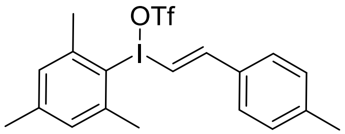
SA20

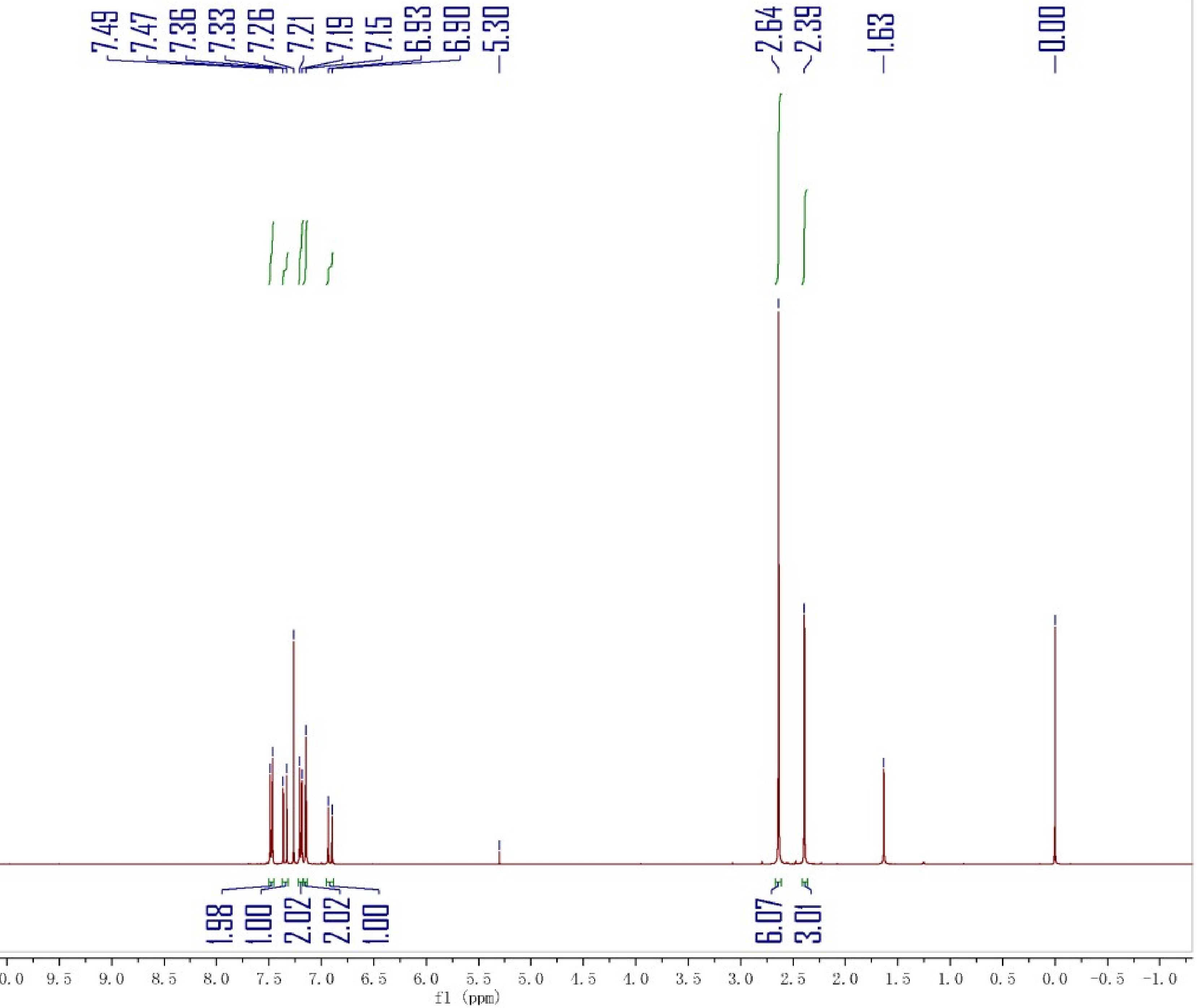

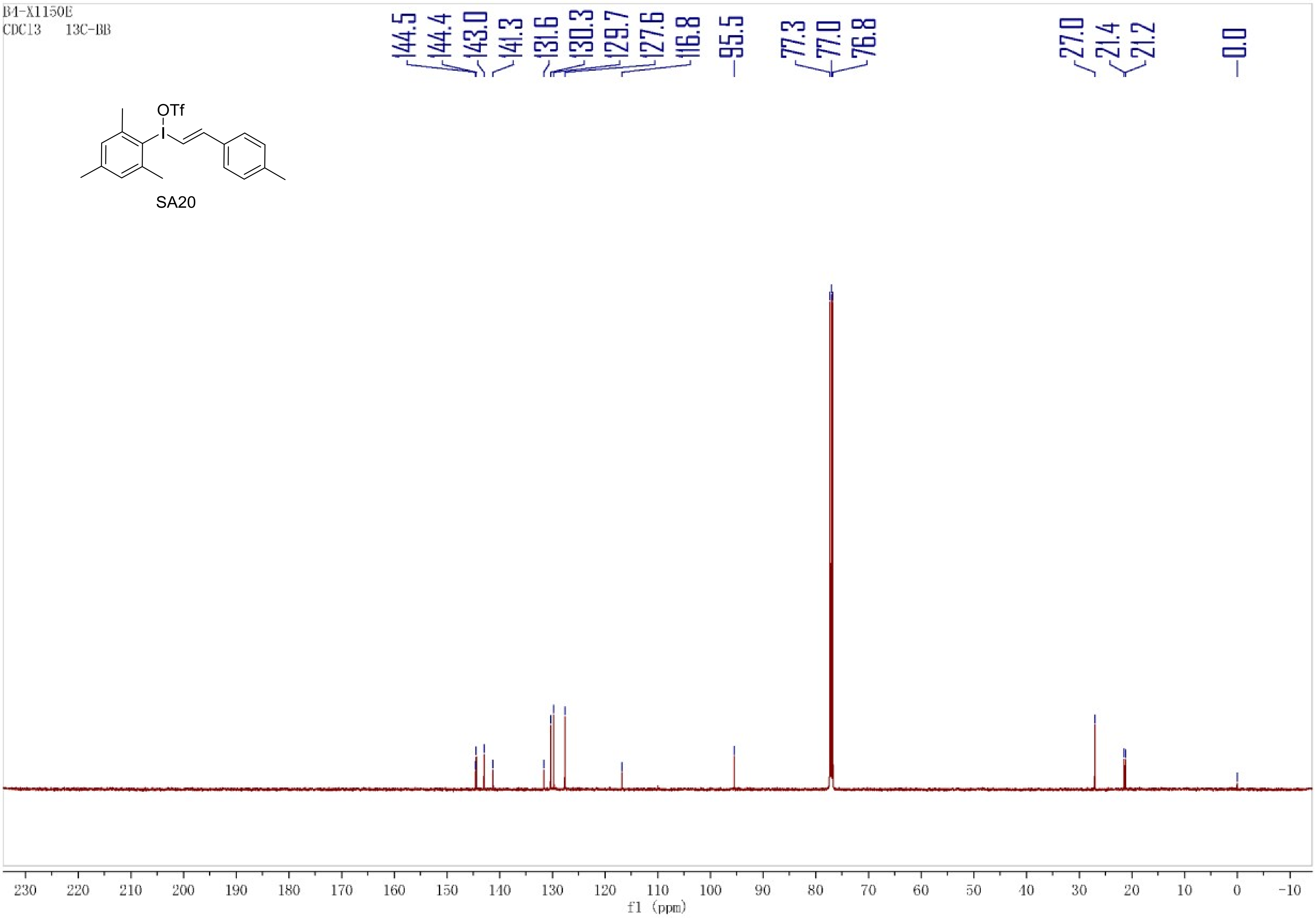

B4-X1150E
CDCl3 13C-BB
OTf
I
SA20
144.5
144.4
143.0
141.3
131.6
130.3
129.7
127.6
116.8
95.5
77.3
77.0
76.8
27.0
21.4
21.2
0.0
230 220 210 200 190 180 170 160 150 140 130 120 110 100 90 80 70 60 50 40 30 20 10 0 -10
f1 (ppm)

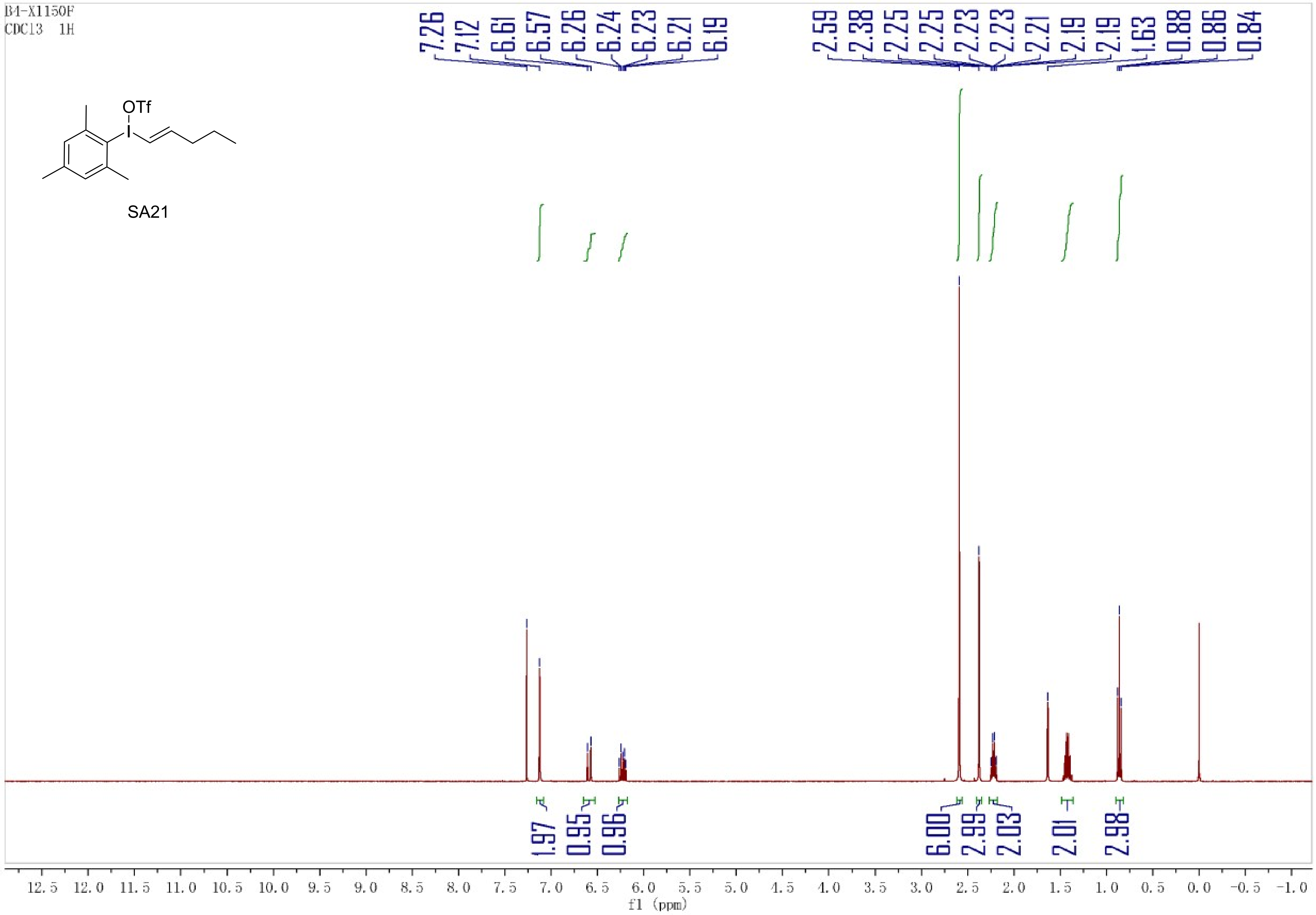
B4-X1150F
CDCl3 1H
OTf
I
SA21
7.26
7.12
6.61
6.57
6.26
6.24
6.23
6.21
6.19
2.59
2.38
2.25
2.25
2.23
2.23
2.21
2.19
2.19
1.63
0.88
0.86
0.84
1.97
0.95
0.96
6.00
2.99
2.03
2.01
2.98
12.5 12.0 11.5 11.0 10.5 10.0 9.5 9.0 8.5 8.0 7.5 7.0 6.5 6.0 5.5 5.0 4.5 4.0 3.5 3.0 2.5 2.0 1.5 1.0 0.5 0.0 -0.5 -1.0
f1 (ppm)

B4-X1150F
CDC13 13C-BB

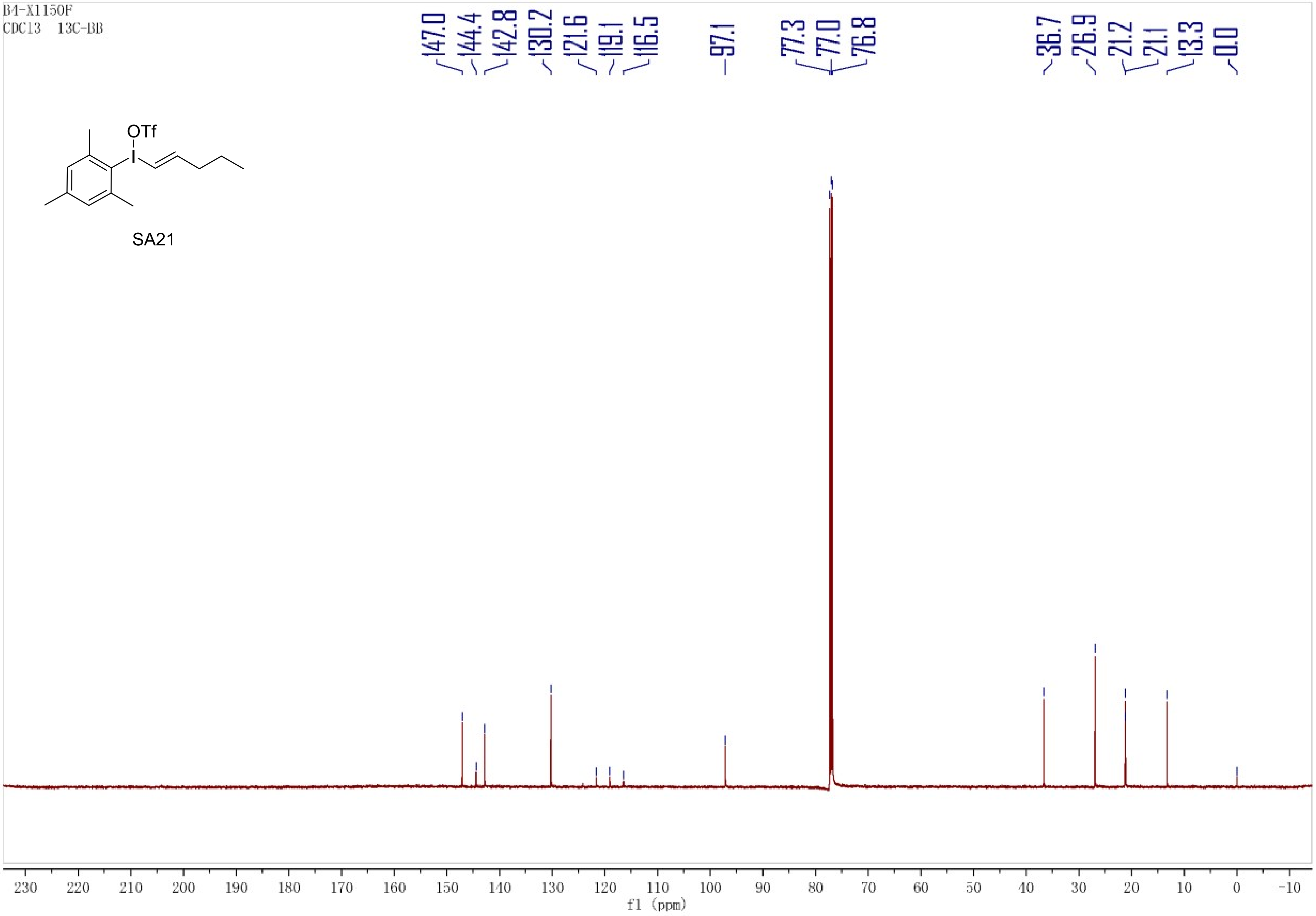

rphs2
rphs2 CDCl3 1H

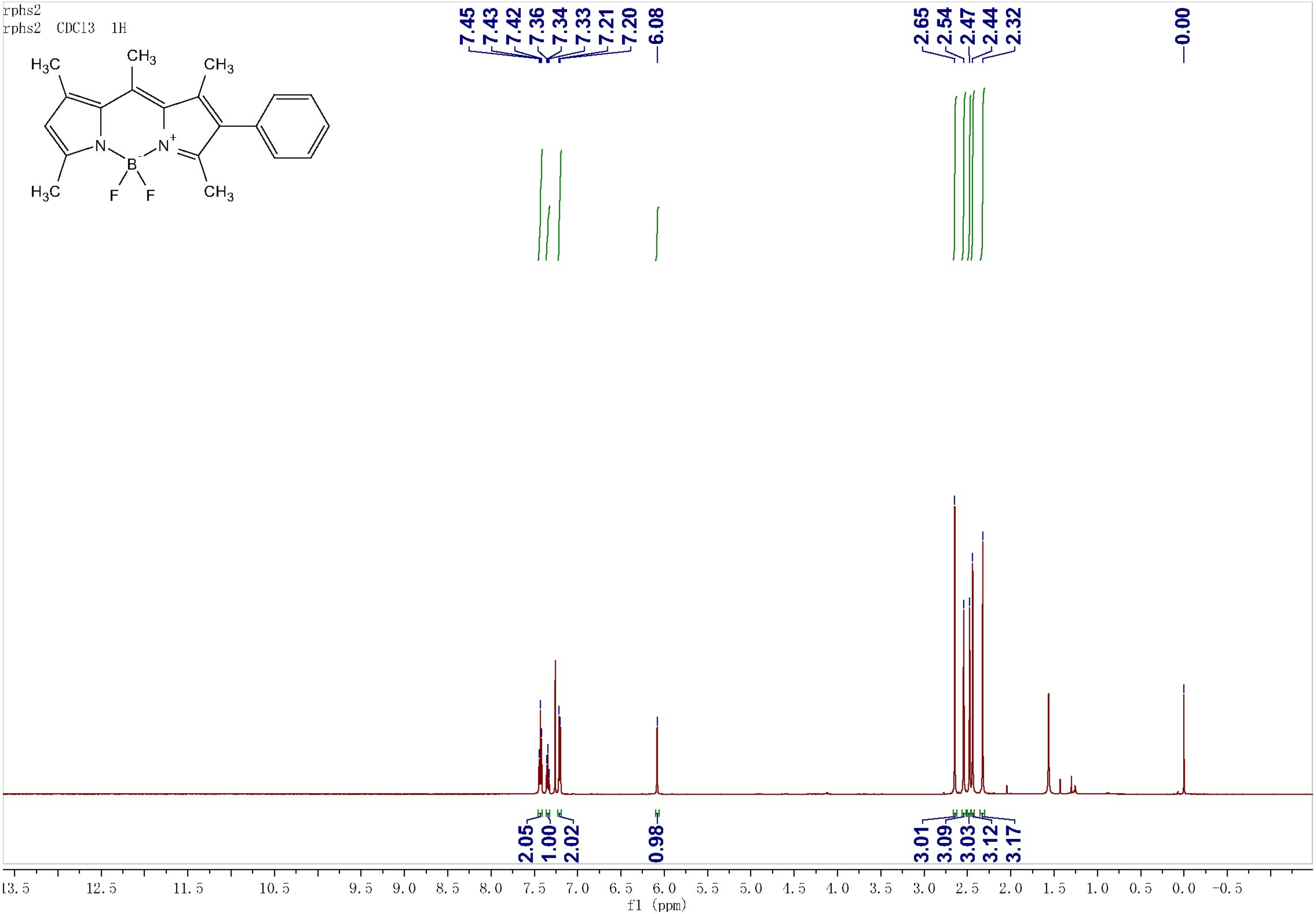

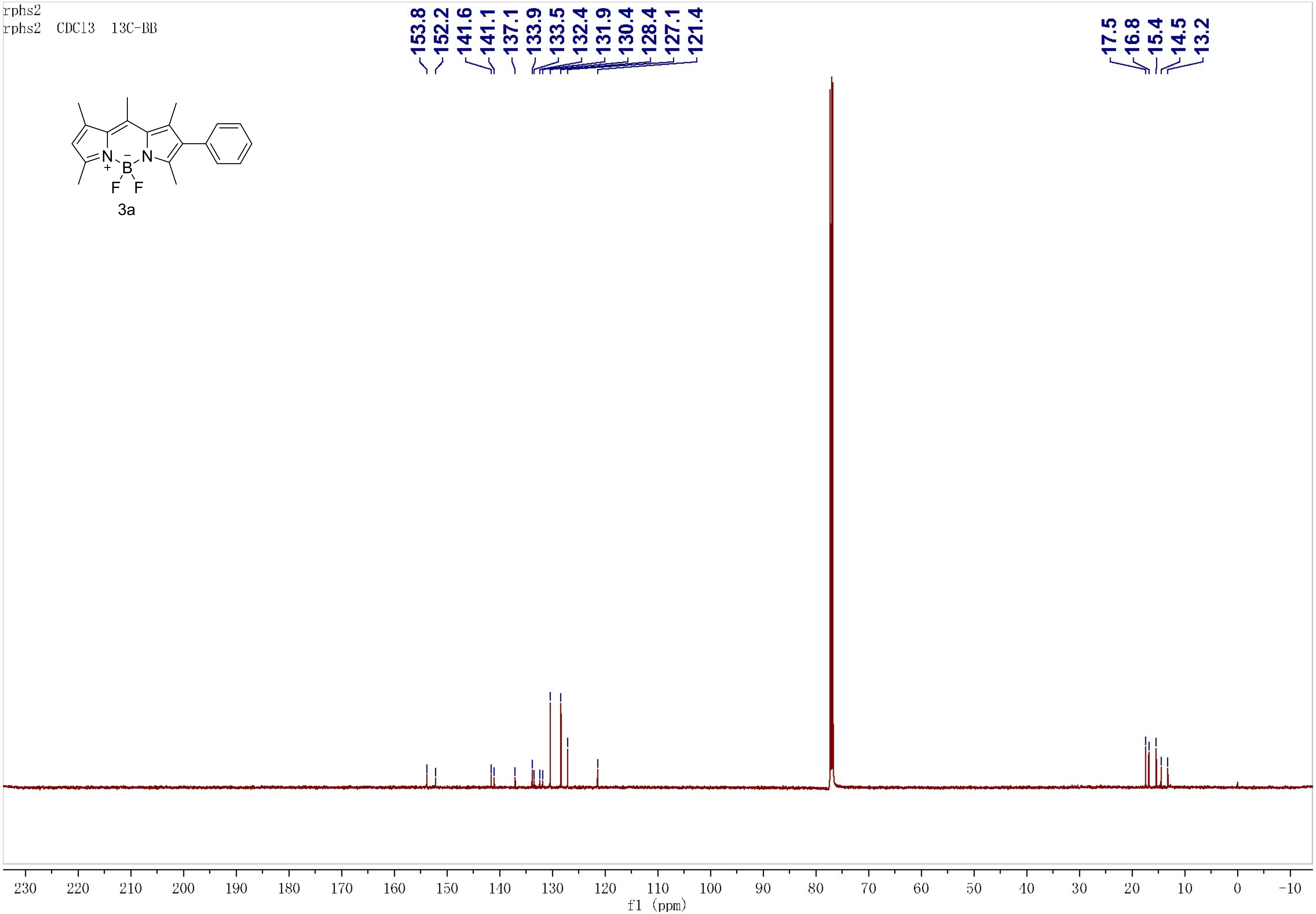
rphs2
rphs2 CDCl3 13C-BB
153.8
152.2
141.6
141.1
137.1
133.9
133.5
132.4
131.9
130.4
128.4
127.1
121.4
17.5
16.8
15.4
14.5
13.2
3a
230 220 210 200 190 180 170 160 150 140 130 120 110 100 90 80 70 60 50 40 30 20 10 0 -10
f1 (ppm)

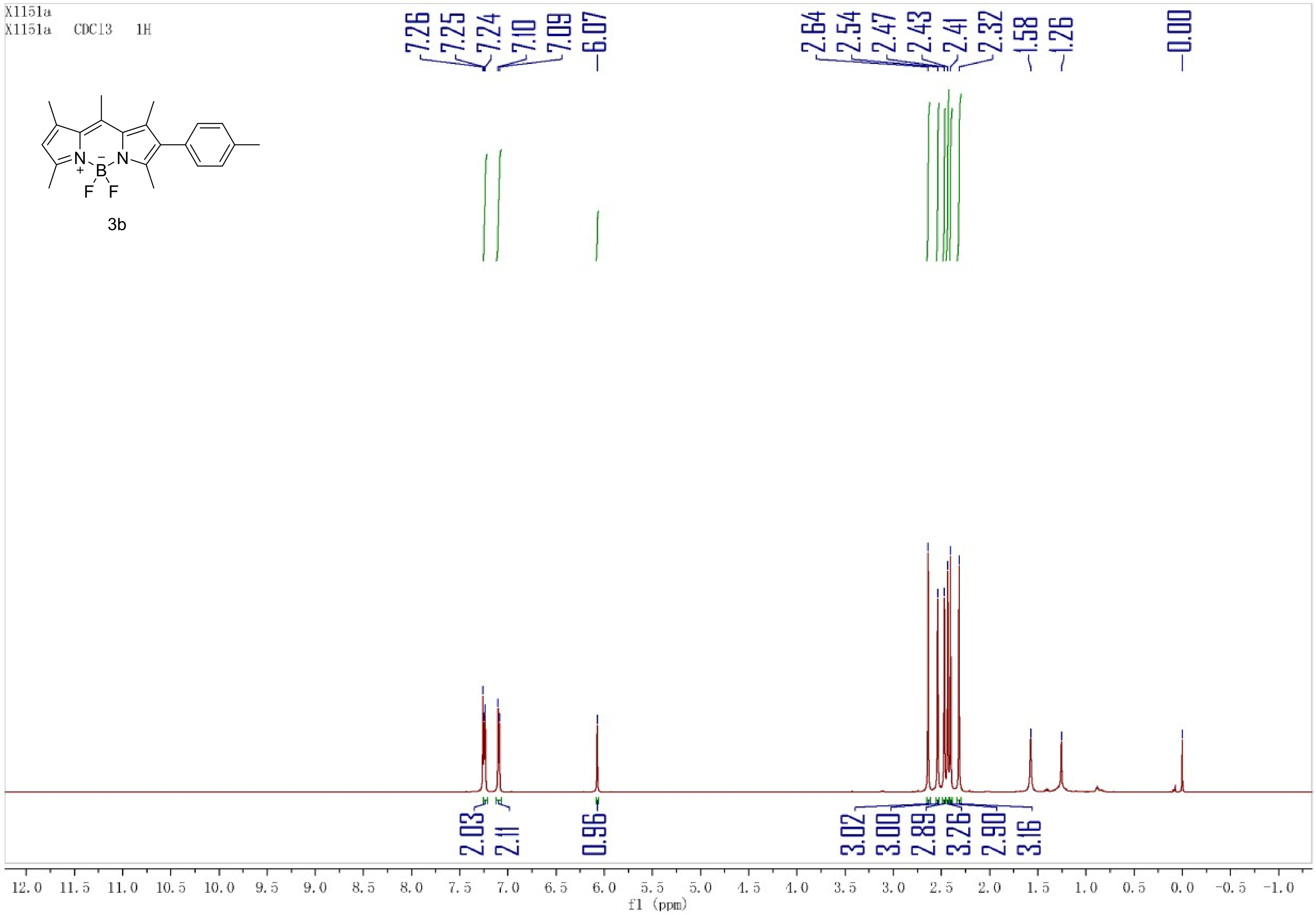
X1151a
X1151a CDCl3 1H
7.26
7.25
7.24
7.10
7.09
6.07
2.64
2.54
2.47
2.43
2.41
2.32
1.58
1.26
0.00
3b
2.03
2.11
0.96
3.02
3.00
2.89
3.26
2.90
3.16
12.0 11.5 11.0 10.5 10.0 9.5 9.0 8.5 8.0 7.5 7.0 6.5 6.0 5.5 5.0 4.5 4.0 3.5 3.0 2.5 2.0 1.5 1.0 0.5 0.0 -0.5 -1.0
f1 (ppm)

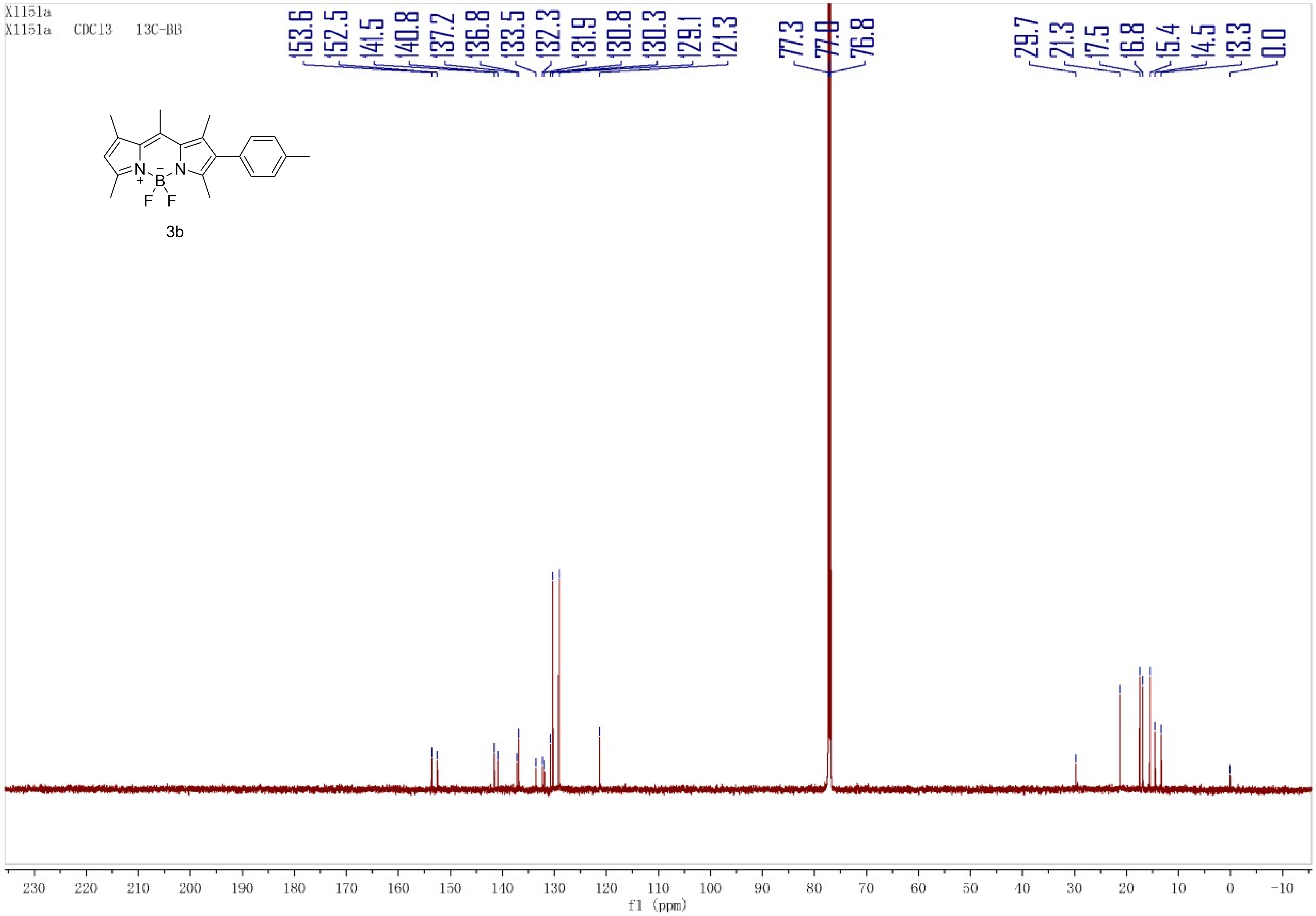
X1151a
X1151a CDCl3 13C-BB
153.6
152.5
141.5
140.8
137.2
136.8
133.5
132.3
131.9
130.8
130.3
129.1
121.3
77.3
77.0
76.8
29.7
21.3
17.5
16.8
15.4
14.5
13.3
0.0
N
N
B
F
F
3b
230 220 210 200 190 180 170 160 150 140 130 120 110 100 90 80 70 60 50 40 30 20 10 0 -10
f1 (ppm)

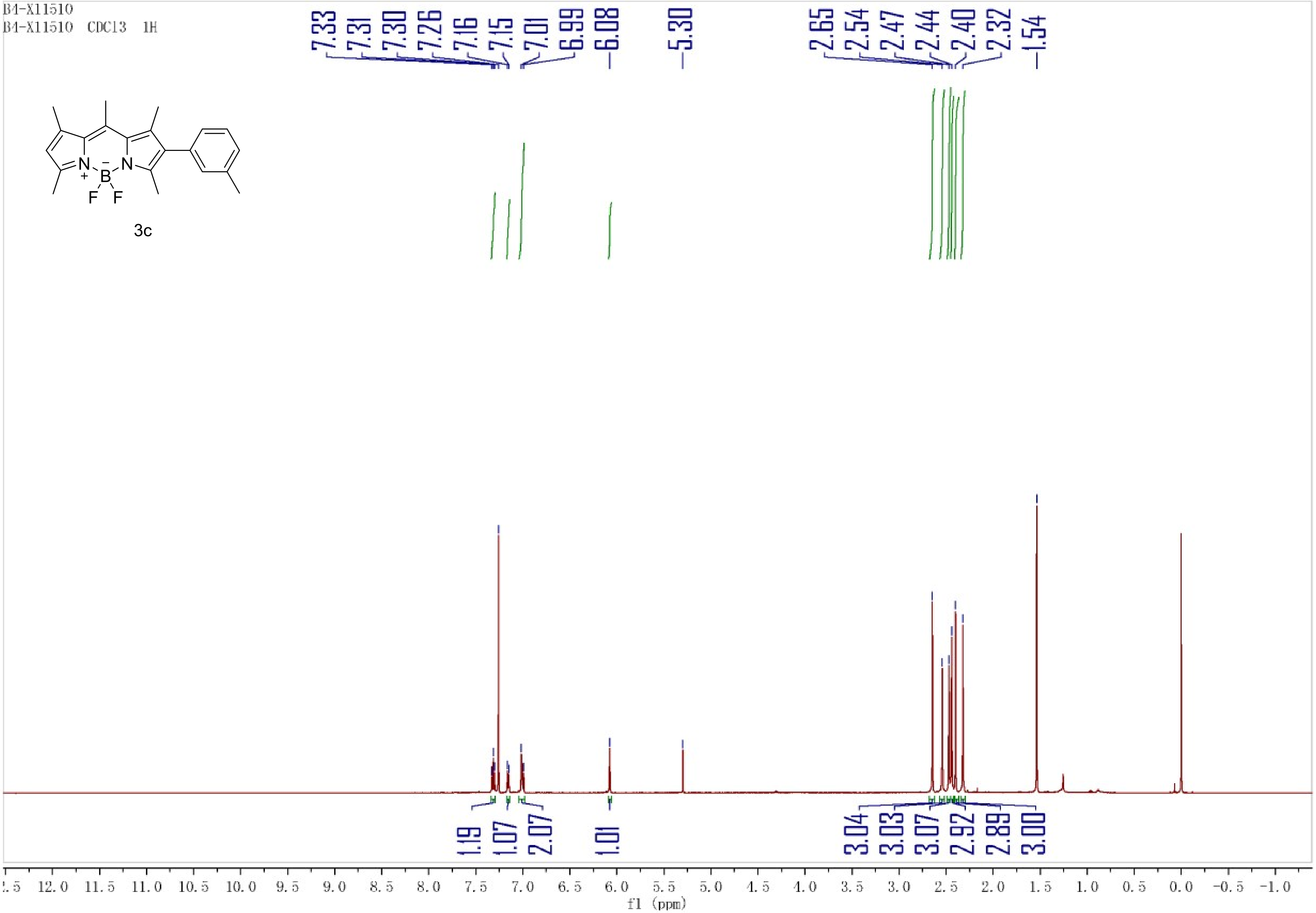
B4-X11510
B4-X11510 CDCl3 1H
7.33
7.31
7.30
7.26
7.16
7.15
7.01
6.99
6.08
5.30
2.65
2.54
2.47
2.44
2.40
2.32
1.54
3c
1.19
1.07
2.07
1.01
3.04
3.03
3.07
2.92
2.89
3.00
f1 (ppm)

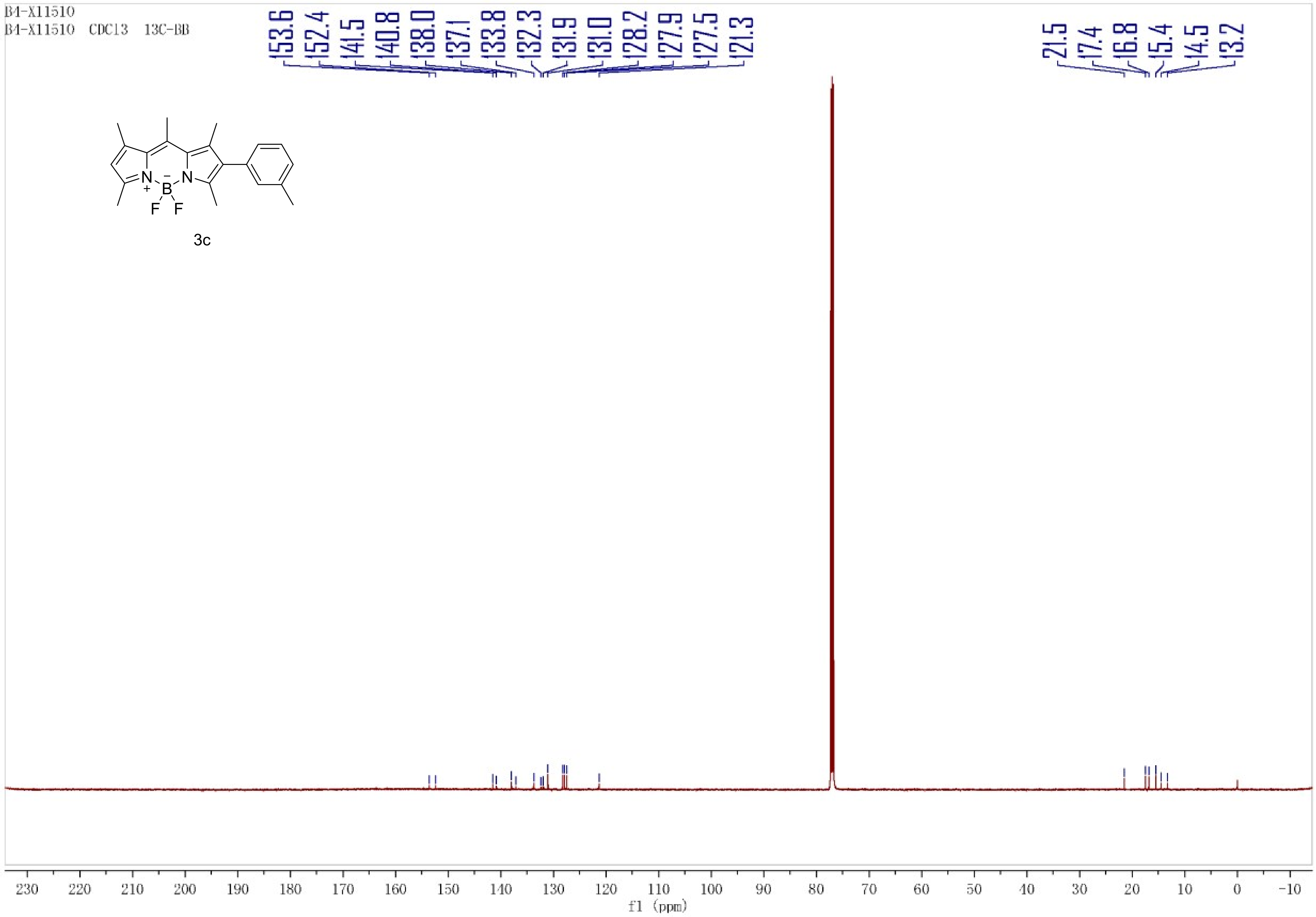

B4-X11510
B4-X11510 CDC13 13C-BB
153.6
152.4
141.5
140.8
138.0
137.1
133.8
132.3
131.9
131.0
128.2
127.9
127.5
121.3
21.5
17.4
16.8
15.4
14.5
13.2
N
B
N
F F
3c
230 220 210 200 190 180 170 160 150 140 130 120 110 100 90 80 70 60 50 40 30 20 10 0 -10
f1 (ppm)

X1151b
X1151b CDCl3 1H

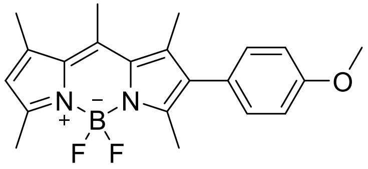

3e

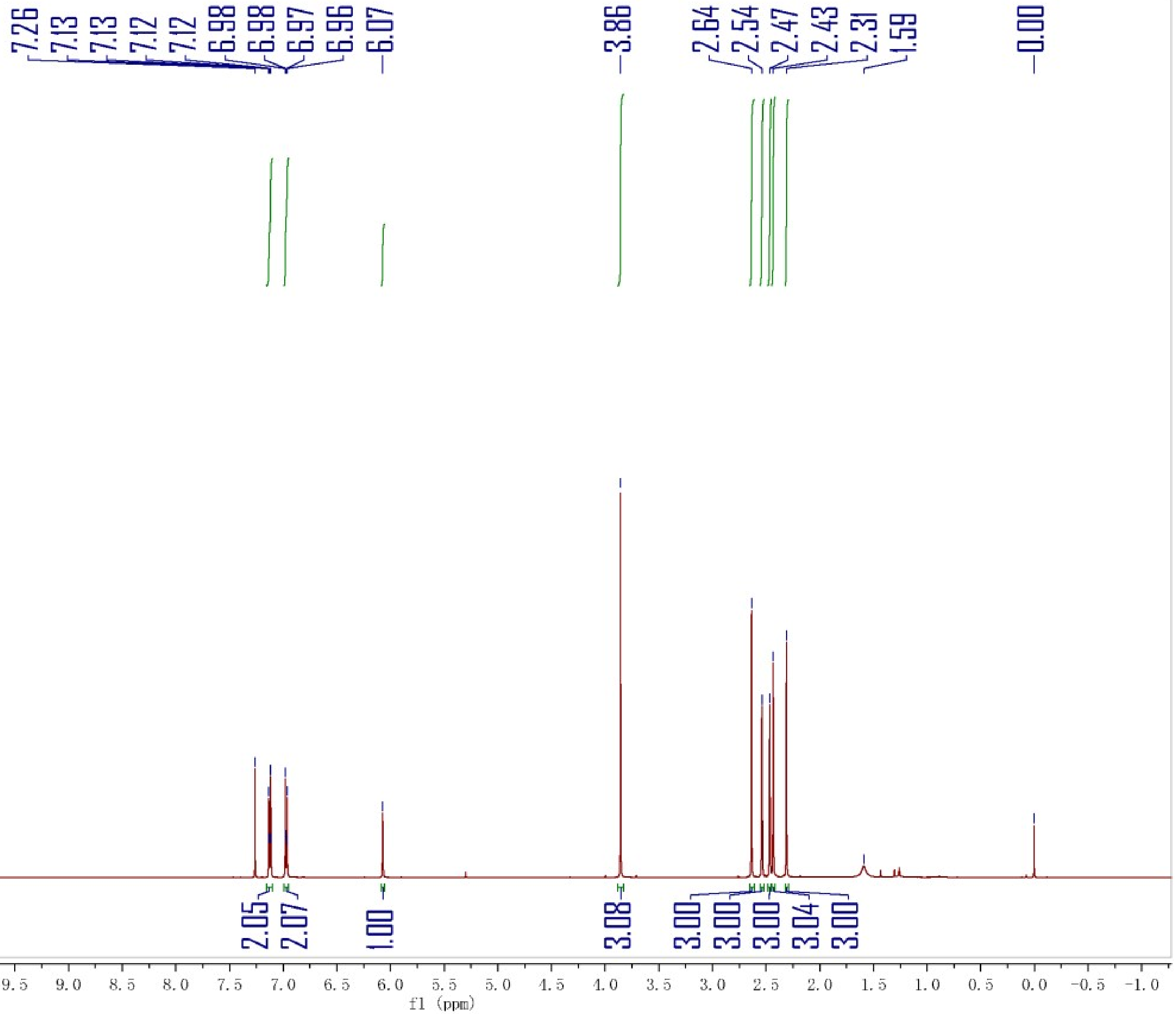

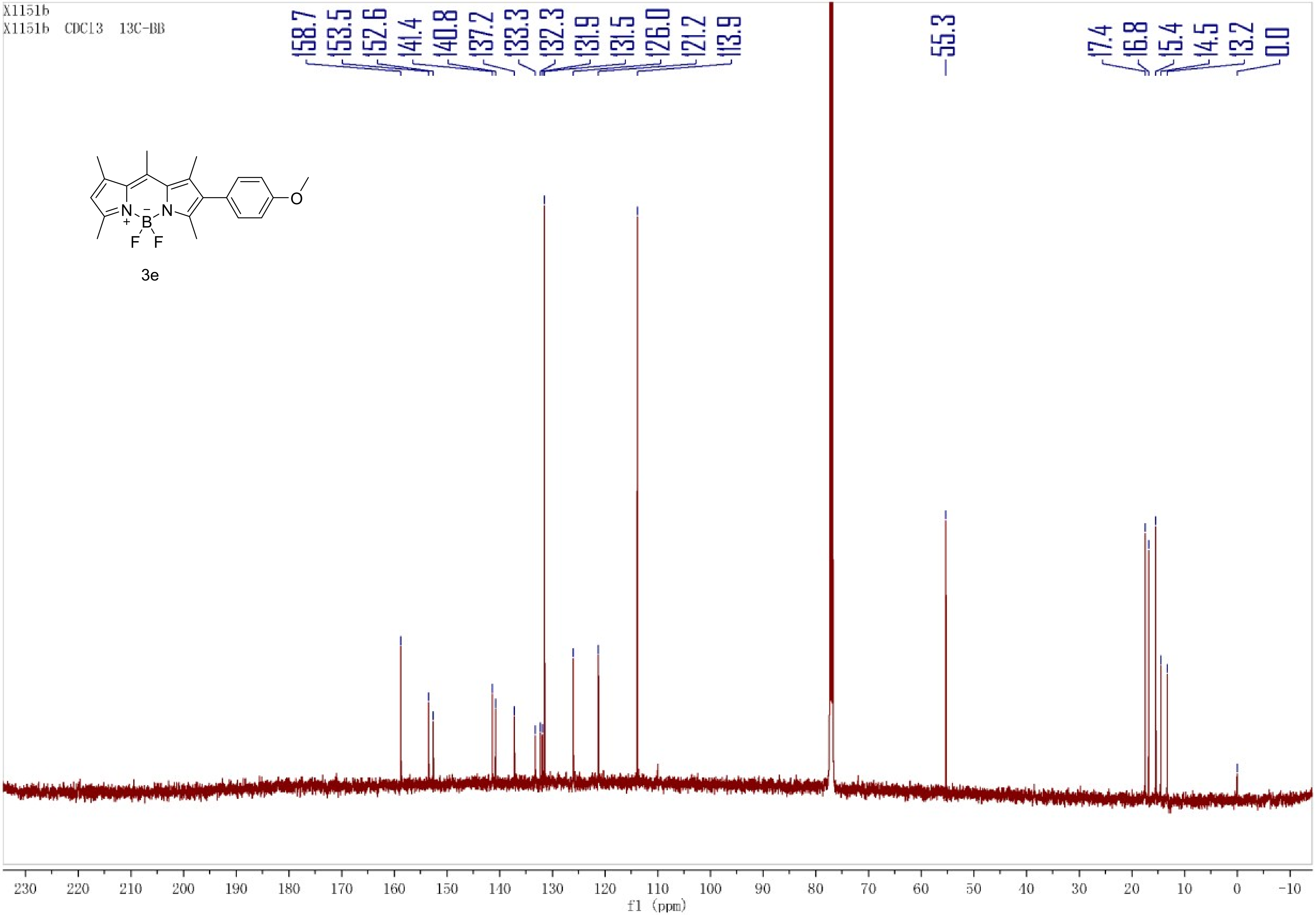

X1151b
X1151b CDCl3 13C-BB
158.7
153.5
152.6
141.4
140.8
137.2
133.3
132.3
131.9
131.5
126.0
121.2
113.9
55.3
17.4
16.8
15.4
14.5
13.2
0.0
230
220
210
200
190
180
170
160
150
140
130
120
110
100
90
80
70
60
50
40
30
20
10
0
-10
f1 (ppm)

3e

X1151c
X1151c CDCl3 1H

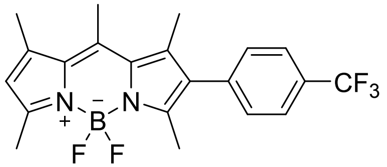

3f

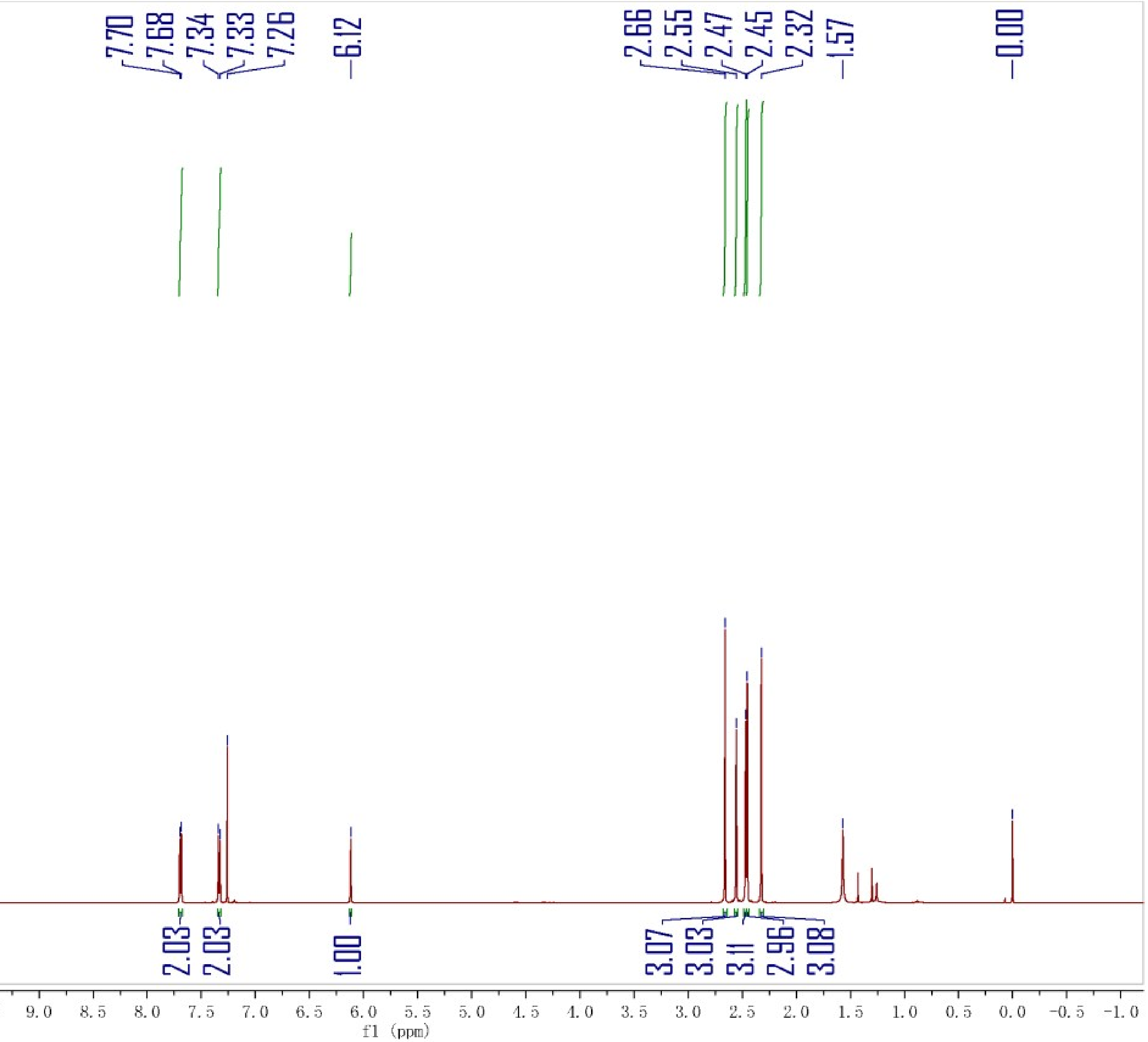

X1151c
X1151c CDCl3 13C-BB

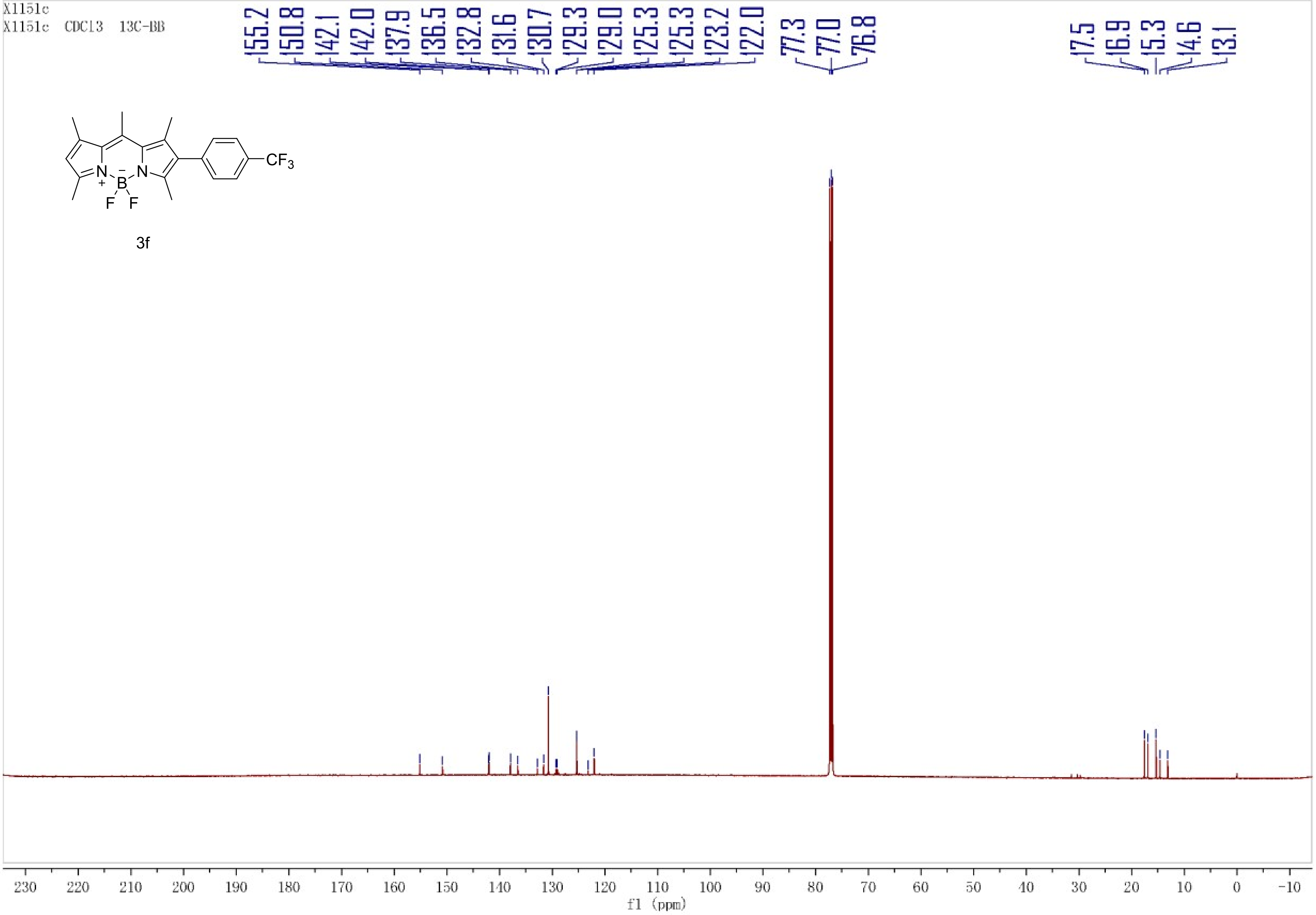

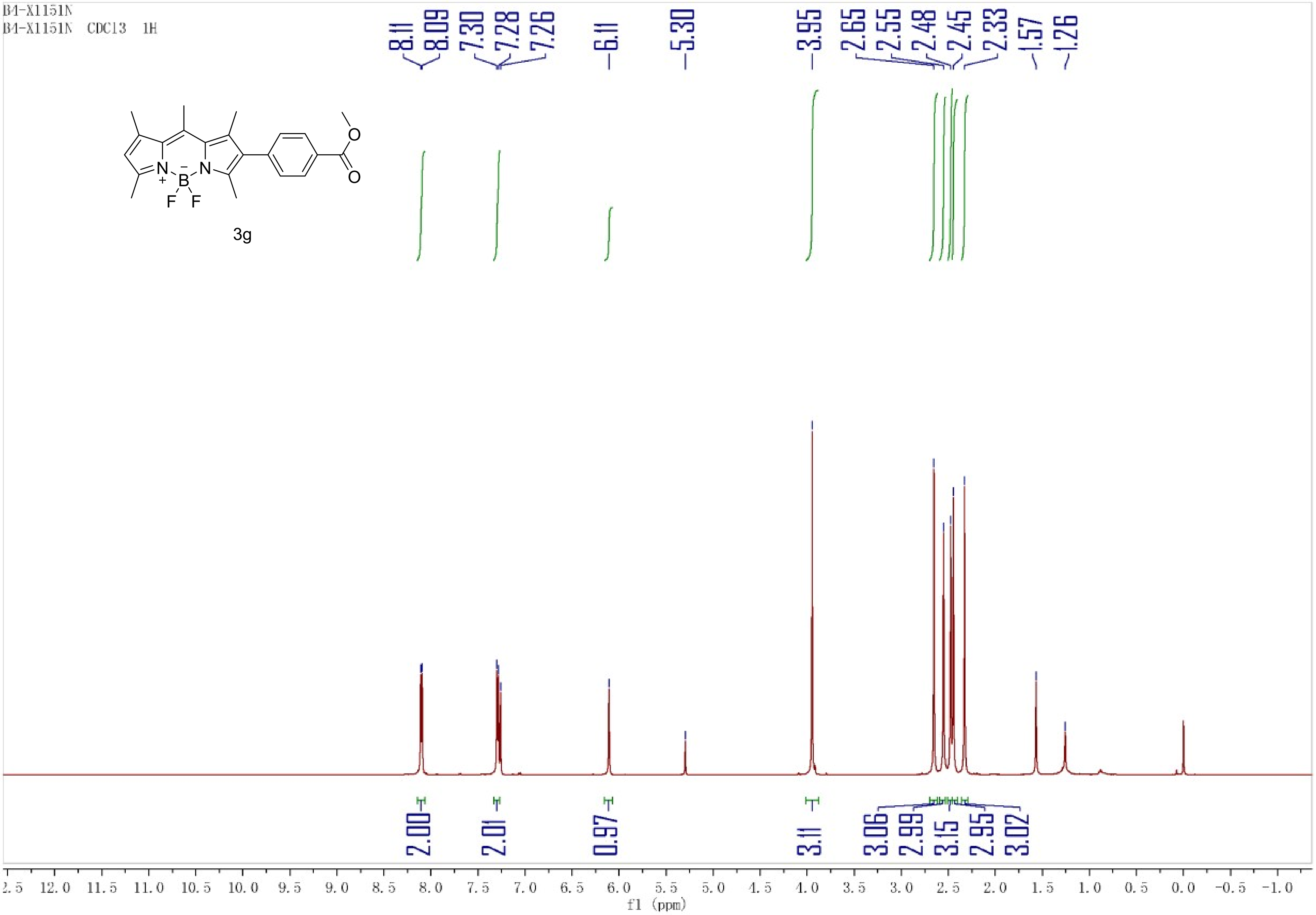
B4-X1151N
B4-X1151N CDCl3 1H
8.11
8.09
7.30
7.28
7.26
6.11
5.30
3.95
2.65
2.55
2.48
2.45
2.33
1.57
1.26
3g
2.00
2.01
0.97
3.11
3.06
2.99
3.15
2.95
3.02
2.5 12.0 11.5 11.0 10.5 10.0 9.5 9.0 8.5 8.0 7.5 7.0 6.5 6.0 5.5 5.0 4.5 4.0 3.5 3.0 2.5 2.0 1.5 1.0 0.5 0.0 -0.5 -1.0
f1 (ppm)

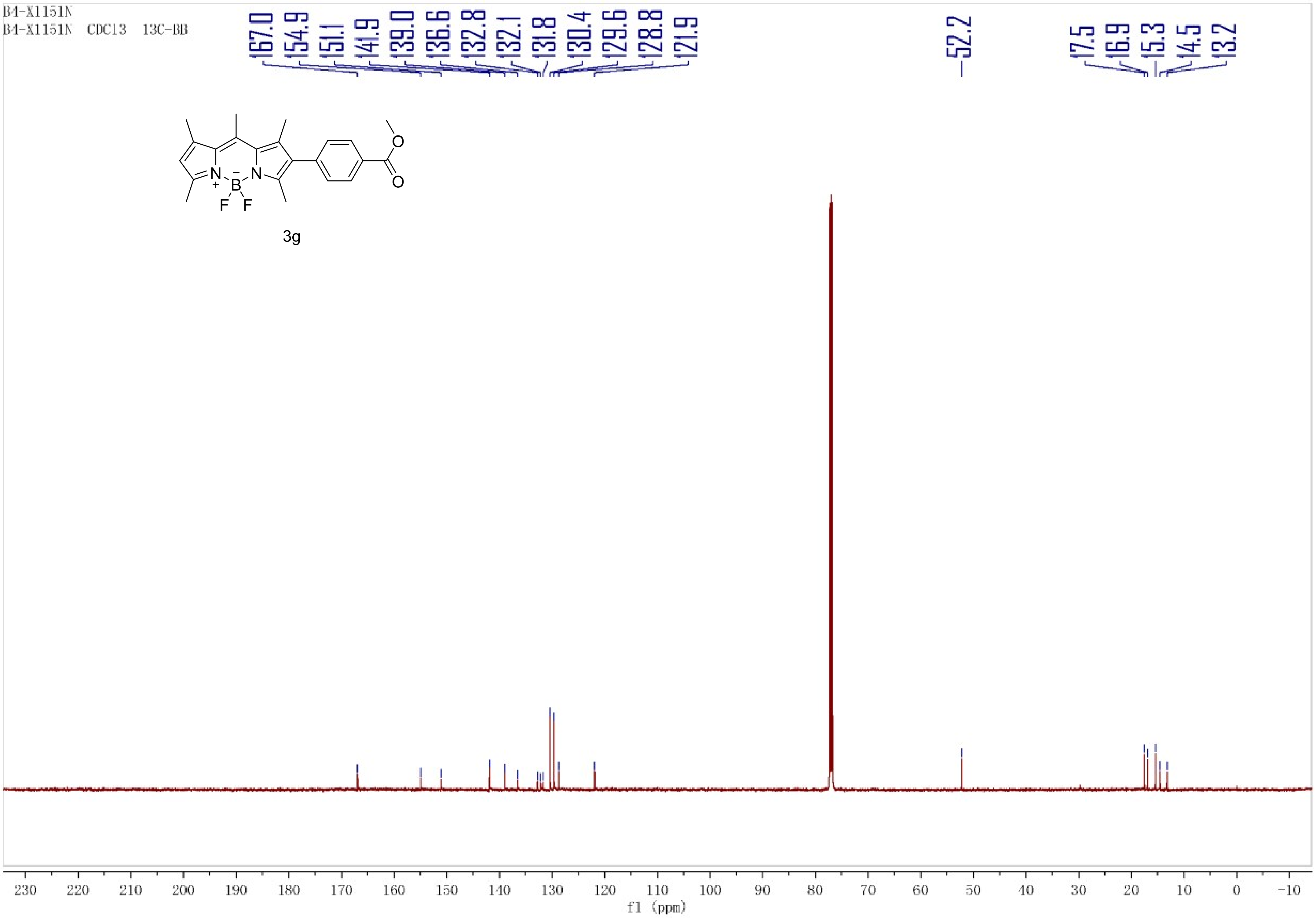

B4-X1151N
B4-X1151N CDCl3 13C-BB
167.0
154.9
151.1
141.9
139.0
136.6
132.8
132.1
131.8
130.4
129.6
128.8
121.9
52.2
17.5
16.9
15.3
14.5
13.2
3g
230 220 210 200 190 180 170 160 150 140 130 120 110 100 90 80 70 60 50 40 30 20 10 0 -10
f1 (ppm)

X1151D
X1151D CDCl3 1H

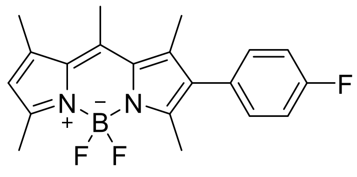

3h

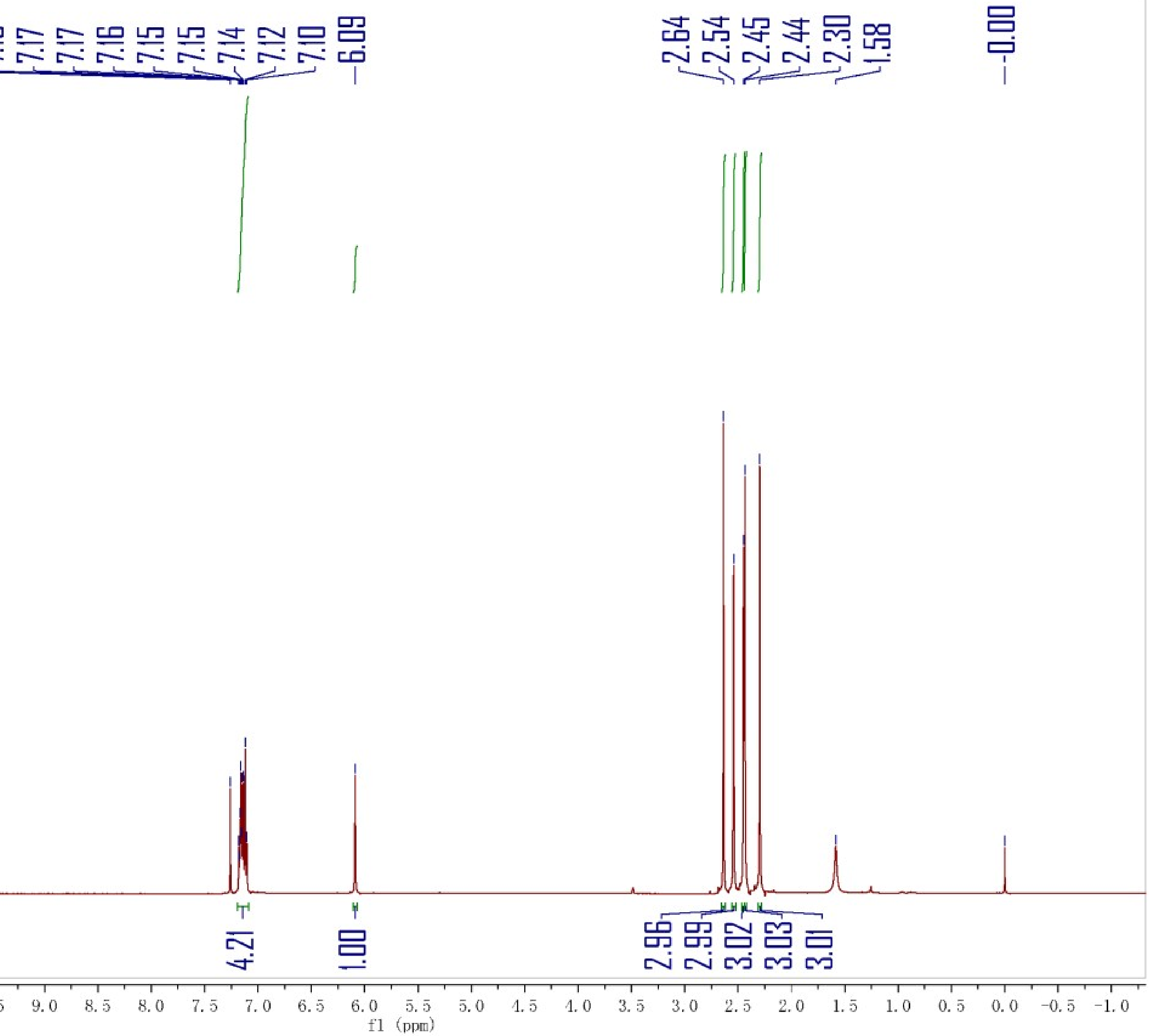

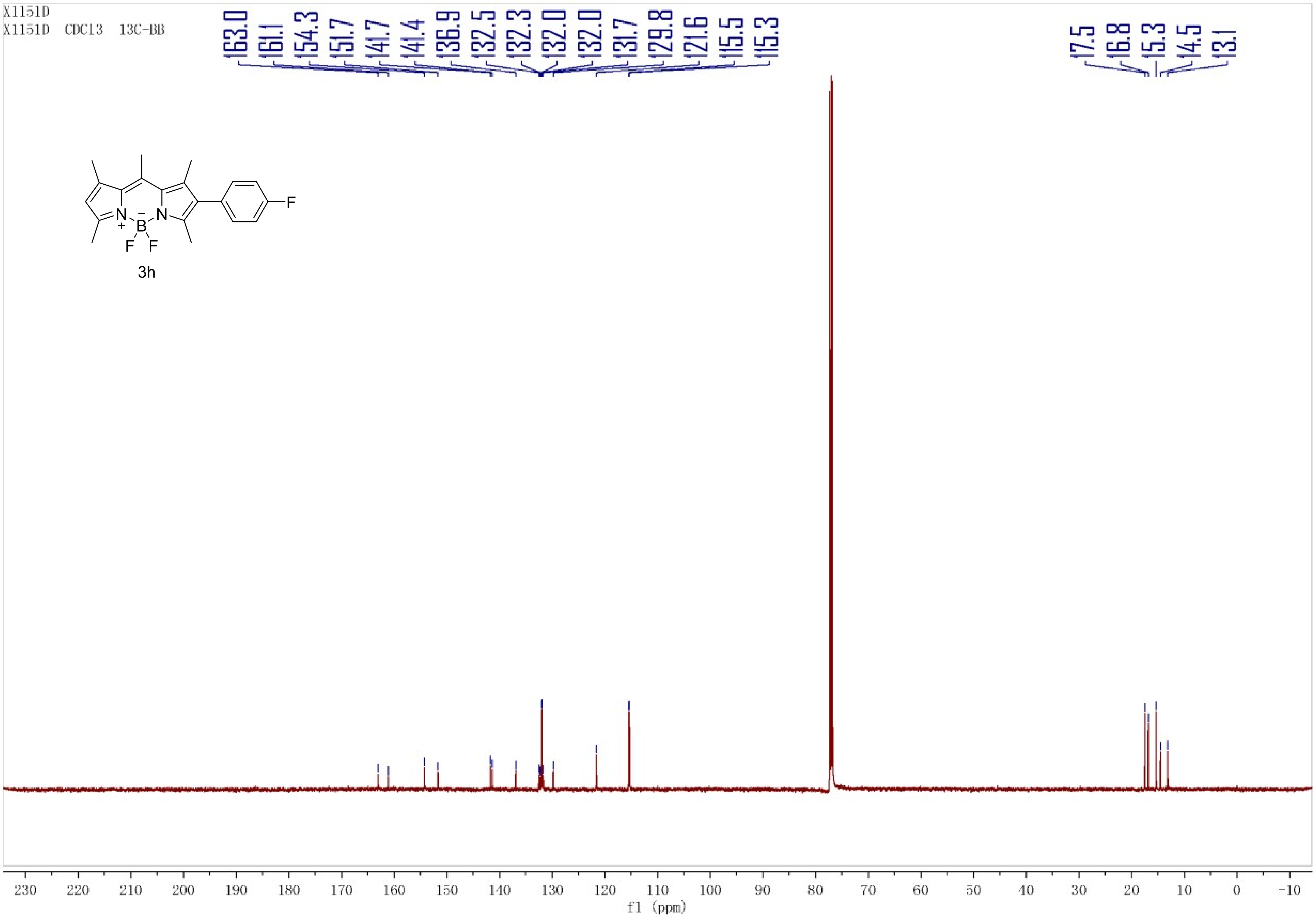
X1151D
X1151D CDCl3 13C-BB
163.0
161.1
154.3
151.7
141.7
141.4
136.9
132.5
132.3
132.0
132.0
131.7
129.8
121.6
115.5
115.3
17.5
16.8
15.3
14.5
13.1
3h
230 220 210 200 190 180 170 160 150 140 130 120 110 100 90 80 70 60 50 40 30 20 10 0 -10
f1 (ppm)

B4-X11511
B4-X11511 CDCl3 1H

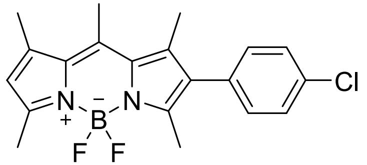

3i

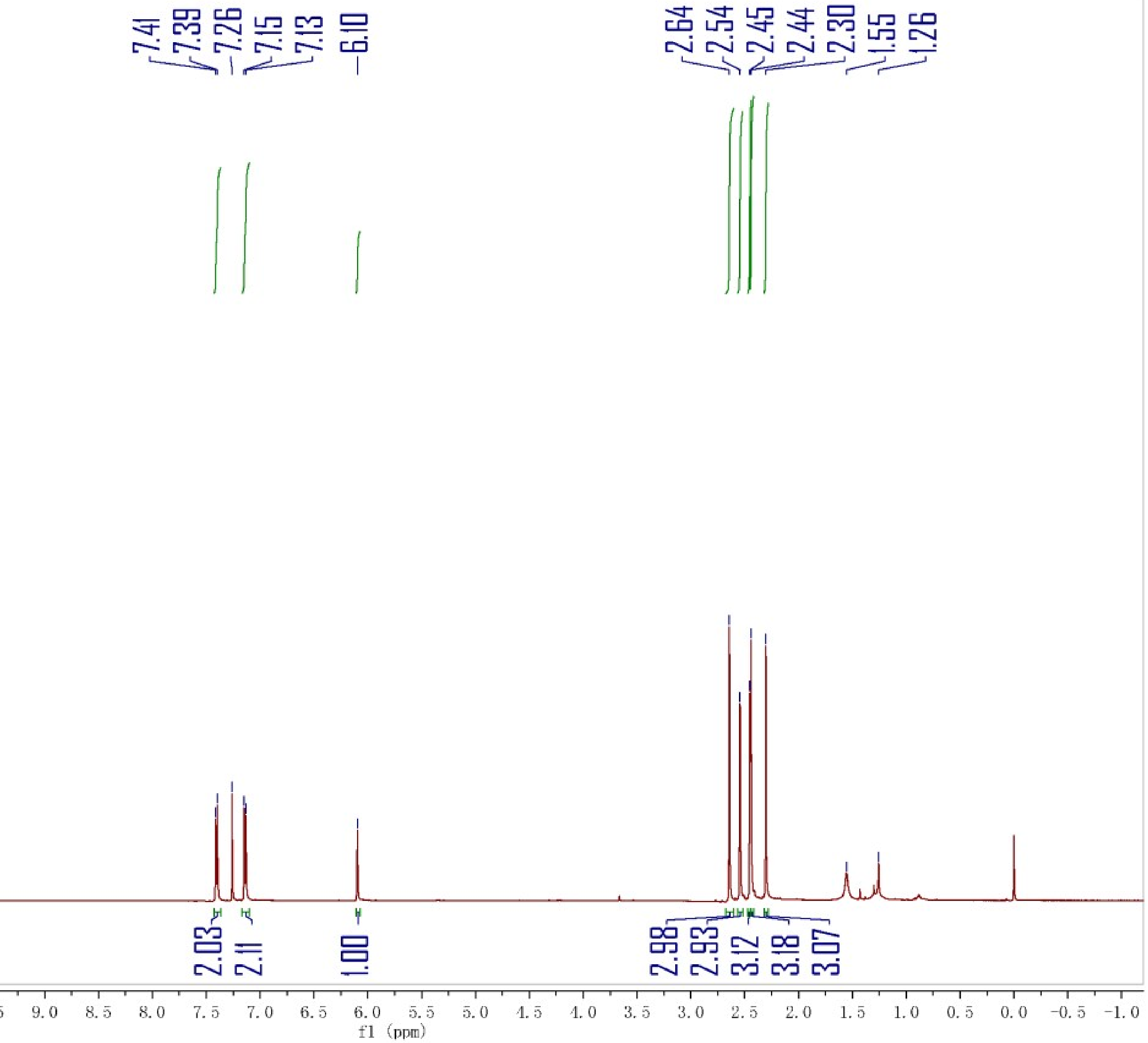

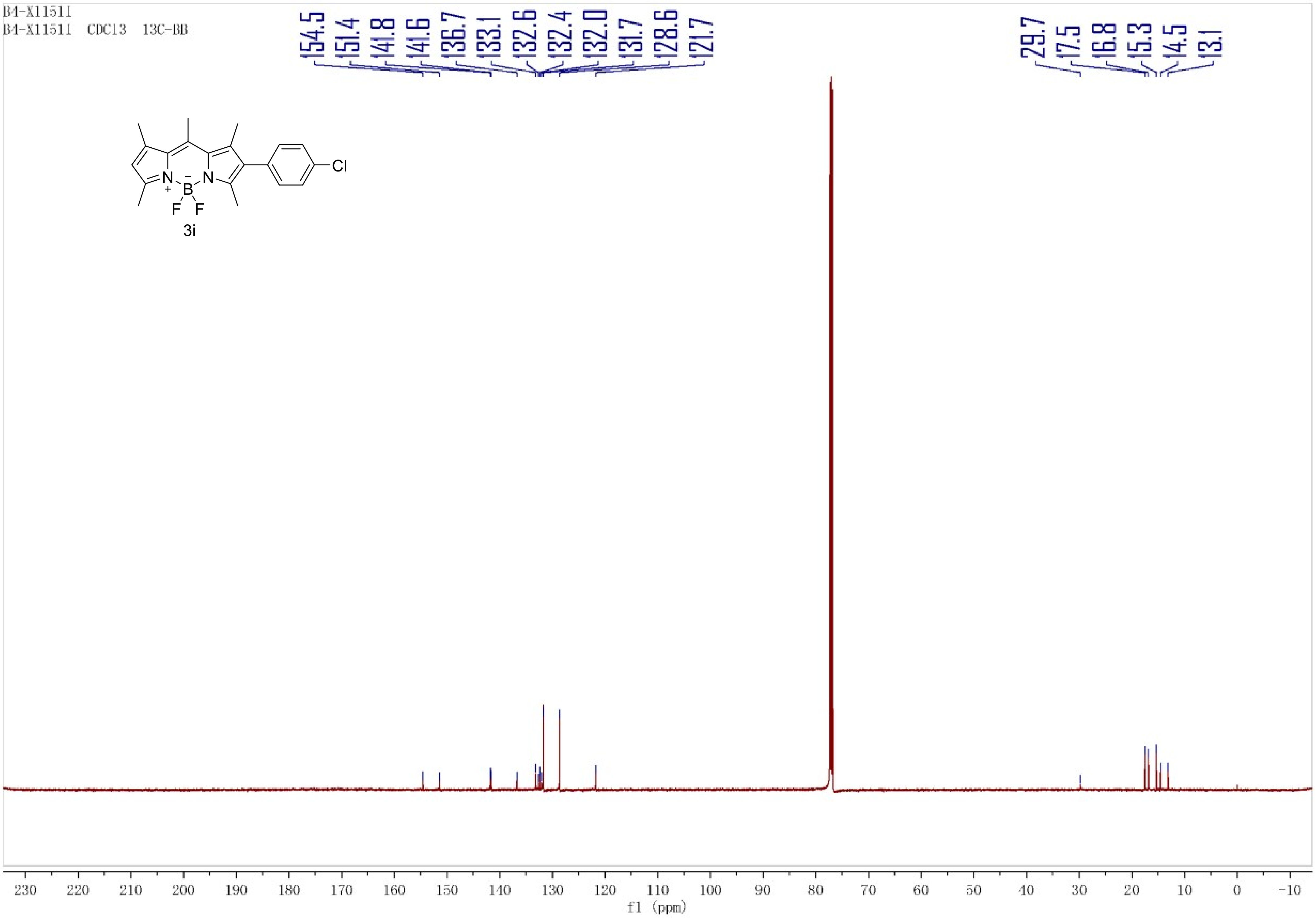
B4-X11511
B4-X11511 CDCl3 13C-BB
154.5
151.4
141.8
141.6
136.7
133.1
132.6
132.4
132.0
131.7
128.6
121.7
29.7
17.5
16.8
15.3
14.5
13.1
Cl
N
B
N
F
F
3i
230
220
210
200
190
180
170
160
150
140
130
120
110
100
90
80
70
60
50
40
30
20
10
0
-10
f1 (ppm)

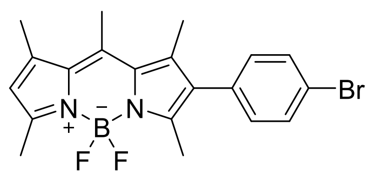

Br
N
B
N
F
F

3j

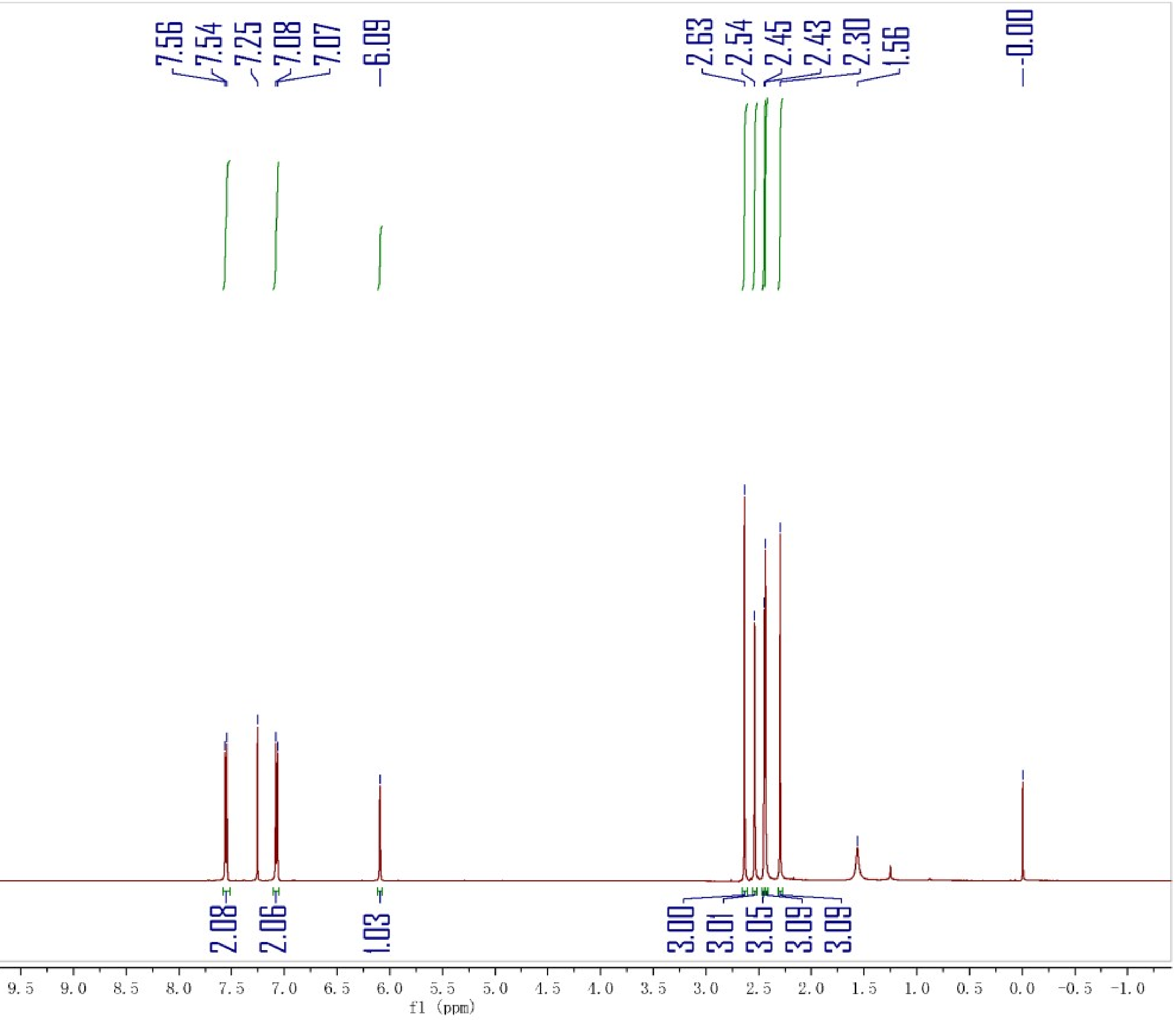

X1151F
X1151F CDCl3 1H
7.56
7.54
7.25
7.08
7.07
6.09
2.63
2.54
2.45
2.43
2.30
1.56
0.00
2.08
2.06
1.03
3.00
3.01
3.05
3.09
3.09
12.0 11.5 11.0 10.5 10.0 9.5 9.0 8.5 8.0 7.5 7.0 6.5 6.0 5.5 5.0 4.5 4.0 3.5 3.0 2.5 2.0 1.5 1.0 0.5 0.0 -0.5 -1.0
f1 (ppm)

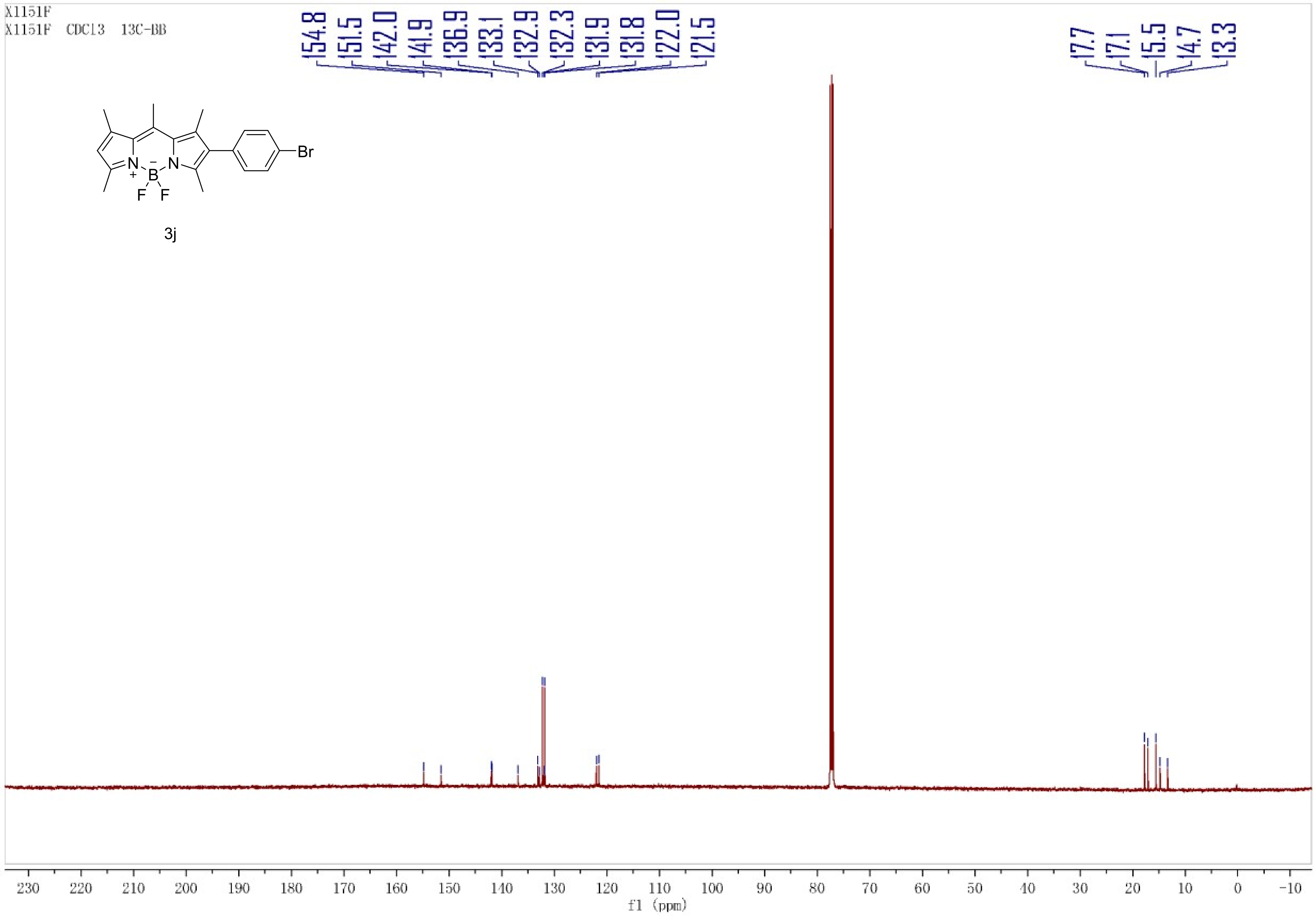

X1151F
X1151F CDCl3 13C-BB
154.8
151.5
142.0
141.9
136.9
133.1
132.9
132.3
131.9
131.8
122.0
121.5
17.7
17.1
15.5
14.7
13.3
N
B
N
F
F
Br
3j
230
220
210
200
190
180
170
160
150
140
130
120
110
100
90
80
70
60
50
40
30
20
10
0
-10
f1 (ppm)

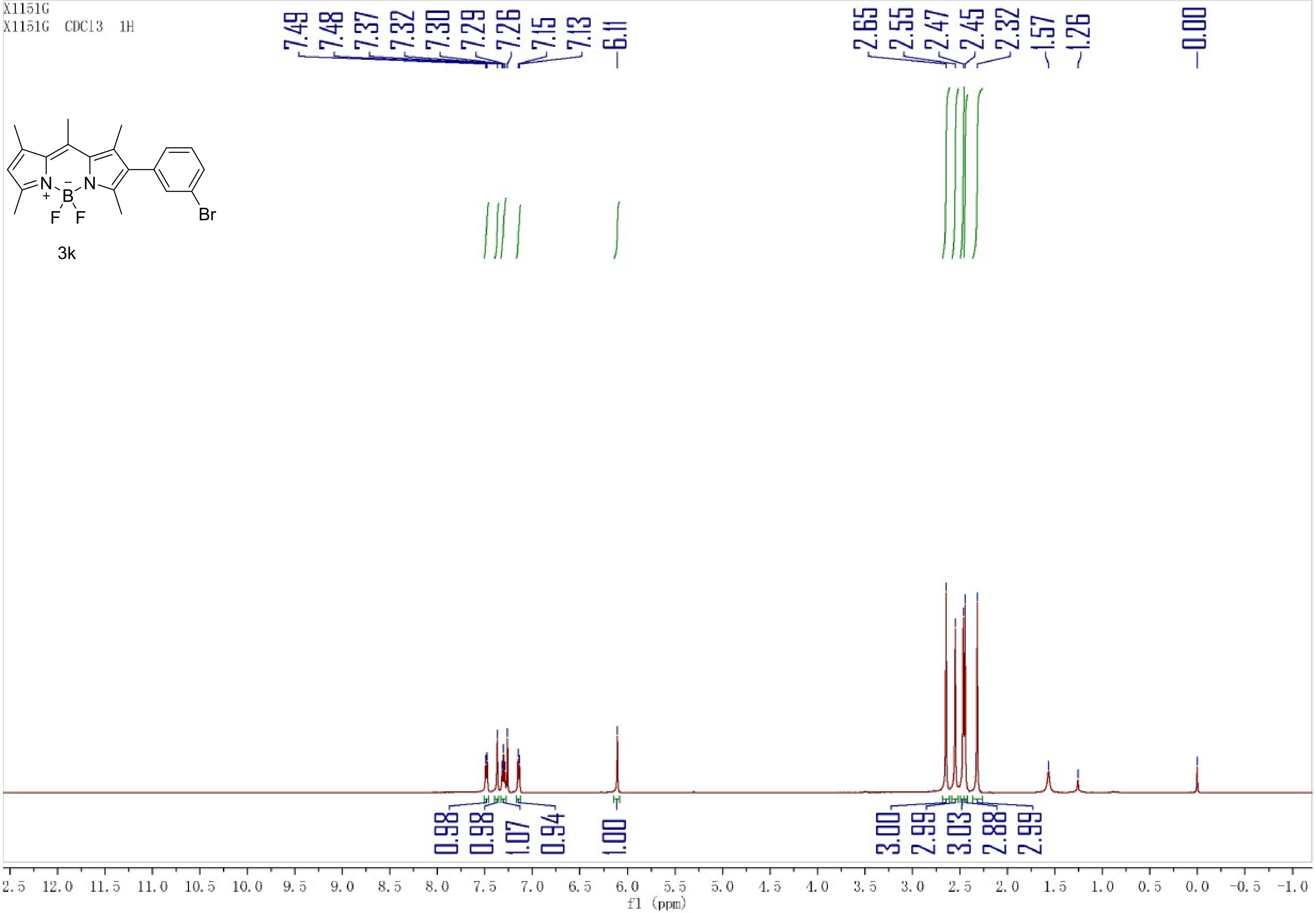

X1151G
X1151G CDCl3 1H
7.49
7.48
7.37
7.32
7.30
7.29
7.26
7.15
7.13
6.11
2.65
2.55
2.47
2.45
2.32
1.57
1.26
0.00
N
N
B
F
F
Br
3k
0.98
0.98
1.07
0.94
1.00
3.00
2.99
3.03
2.88
2.99
2.5
12.0
11.5
11.0
10.5
10.0
9.5
9.0
8.5
8.0
7.5
7.0
6.5
6.0
5.5
5.0
4.5
4.0
3.5
3.0
2.5
2.0
1.5
1.0
0.5
0.0
-0.5
-1.0
f1 (ppm)

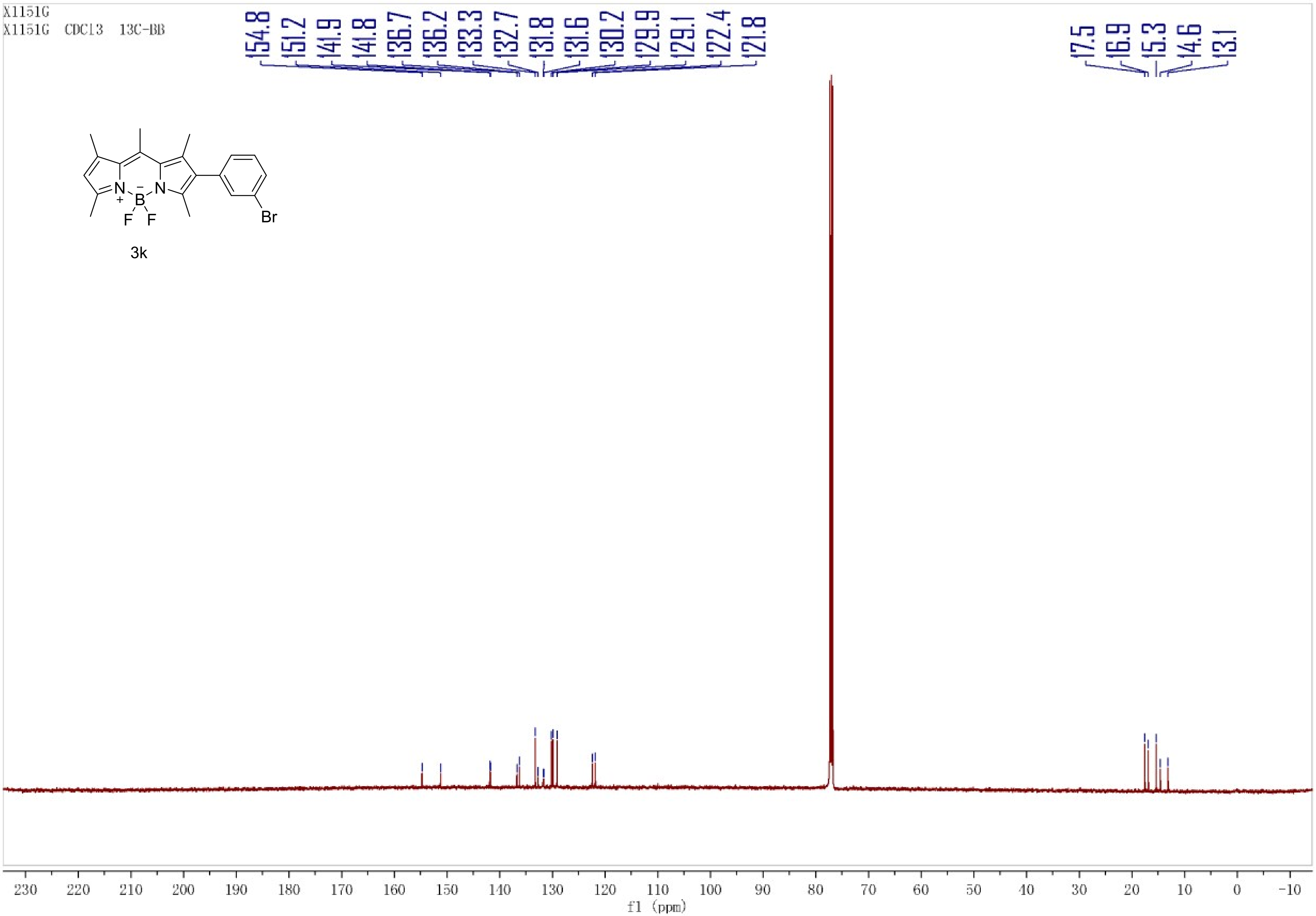

X1151G
X1151G CDCl3 13C-BB
154.8
151.2
141.9
141.8
136.7
136.2
133.3
132.7
131.8
131.6
130.2
129.9
129.1
122.4
121.8
17.5
16.9
15.3
14.6
13.1
N
B
N
F
F
Br
3k
230
220
210
200
190
180
170
160
150
140
130
120
110
100
90
80
70
60
50
40
30
20
10
0
-10
f1 (ppm)

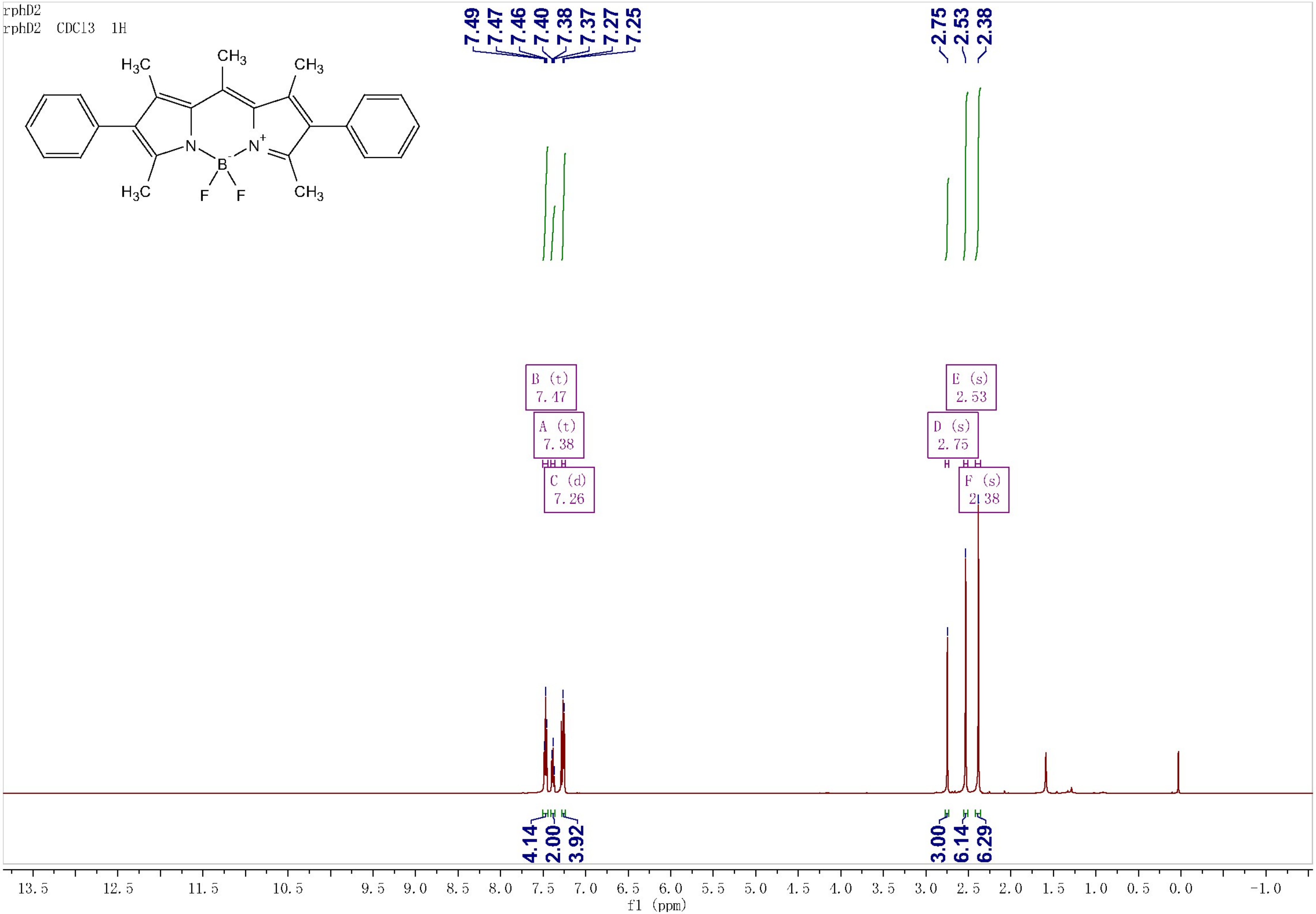
rphD2
rphD2 CDCl3 1H
7.49
7.47
7.46
7.40
7.38
7.37
7.27
7.25
2.75
2.53
2.38
B (t)
7.47
A (t)
7.38
C (d)
7.26
E (s)
2.53
D (s)
2.75
F (s)
2.38
4.14
2.00
3.92
3.00
6.14
6.29
13.5
12.5
11.5
10.5
9.5
9.0
8.5
8.0
7.5
7.0
6.5
6.0
5.5
5.0
4.5
4.0
3.5
3.0
2.5
2.0
1.5
1.0
0.5
0.0
-1.0
f1 (ppm)

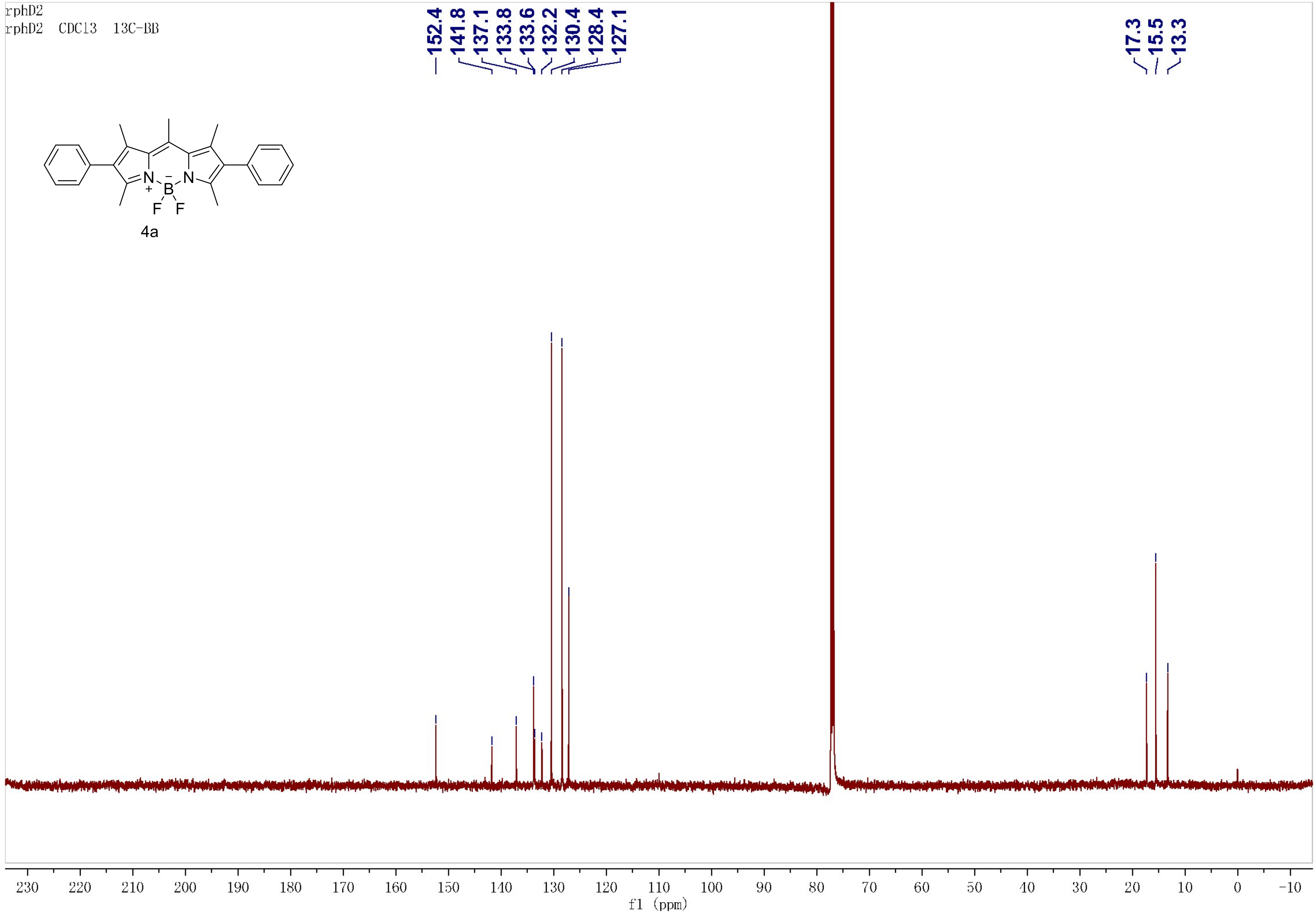

rphD2
rphD2 CDCl3 13C-BB
152.4
141.8
137.1
133.8
133.6
132.2
130.4
128.4
127.1
17.3
15.5
13.3
4a
f1 (ppm)

B4-X1151R
B4-X1151R CDCl3 1H

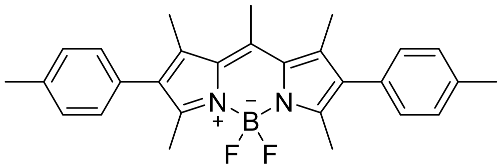

4b

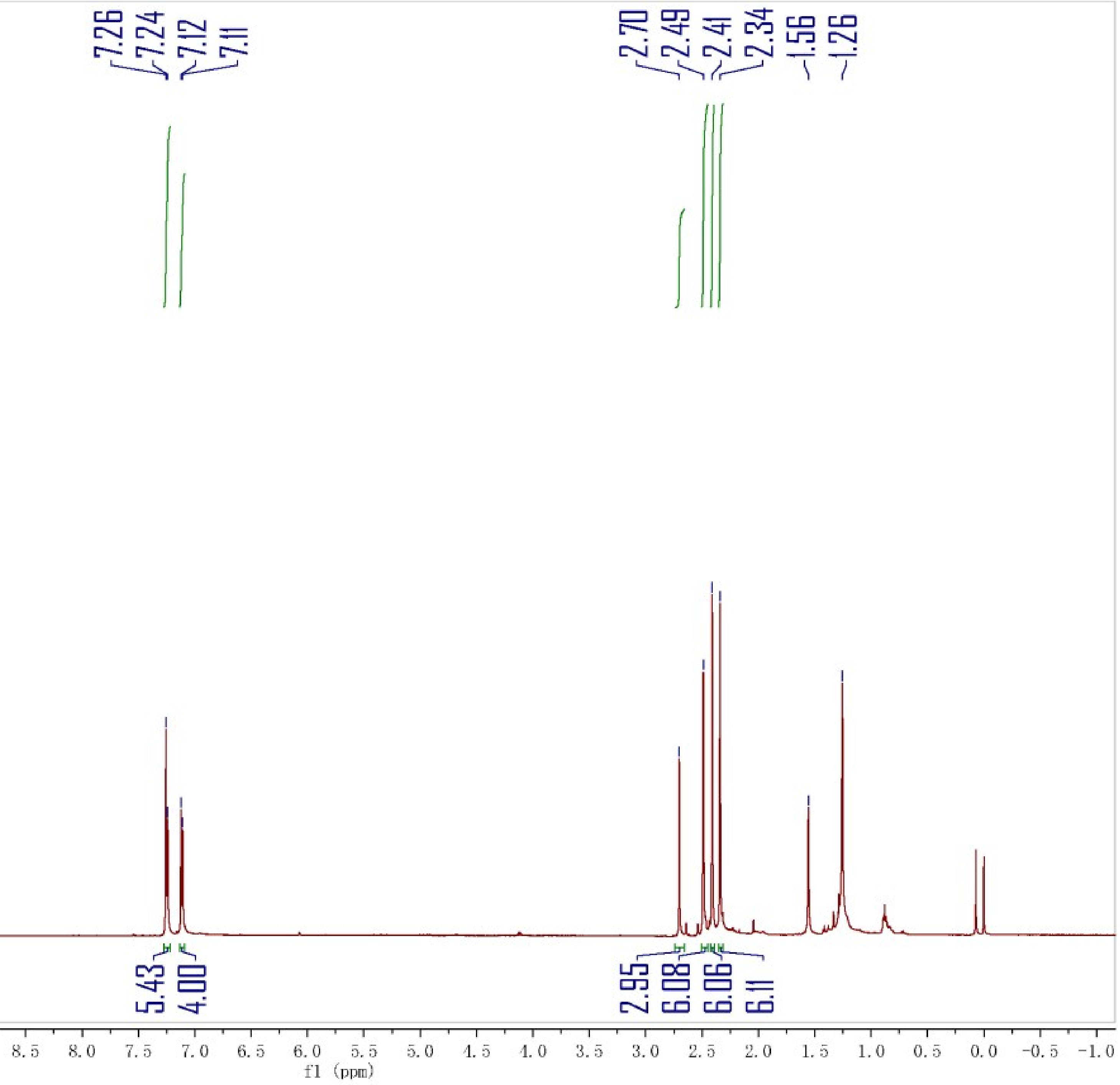

B4-X1151R
B4-X1151R CDCl3 13C-BB

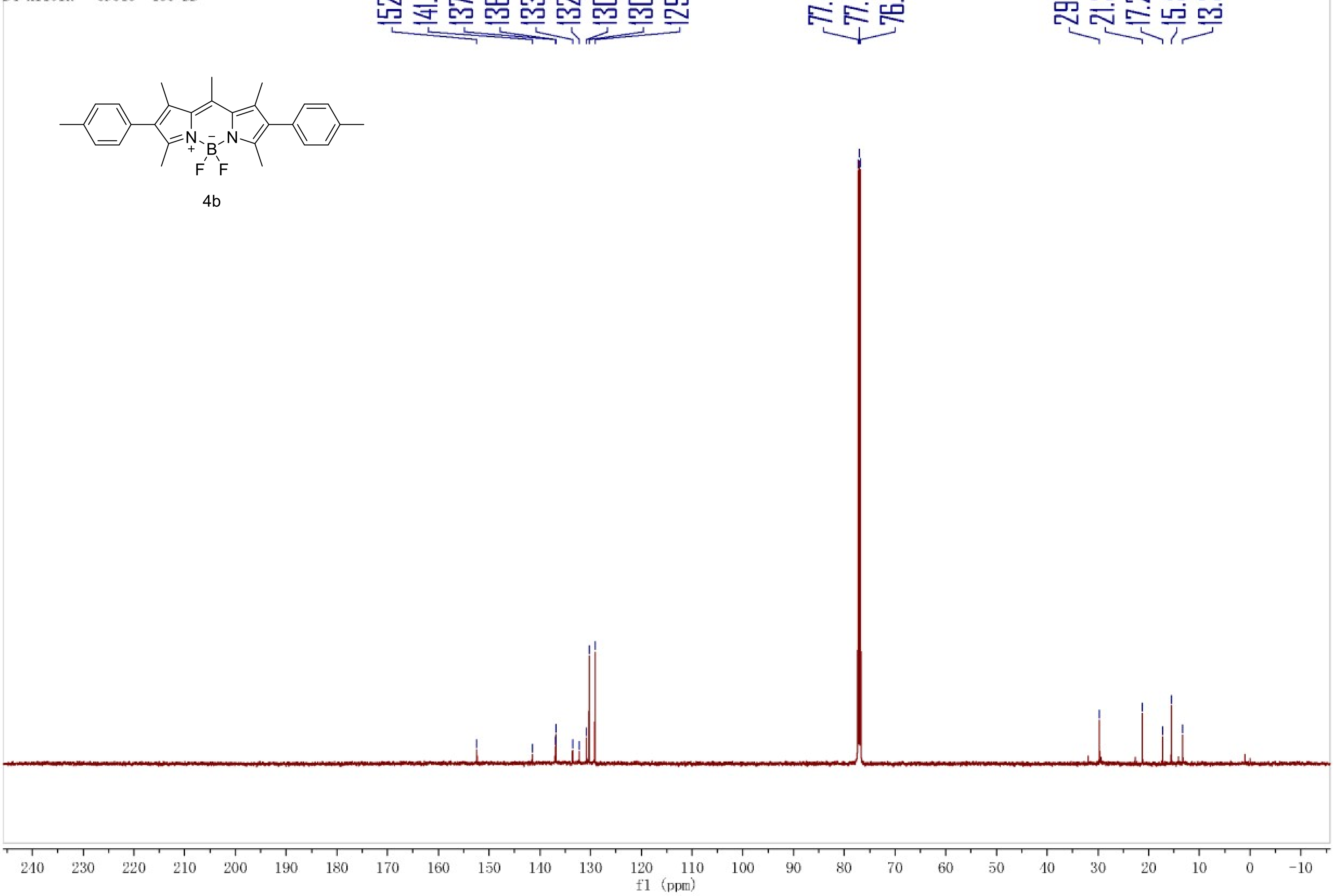

B4-X1151P
B4-X1151P CDCl3 1H

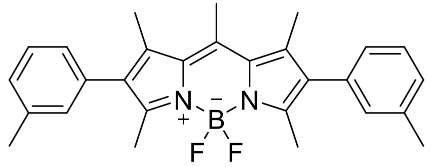

4c

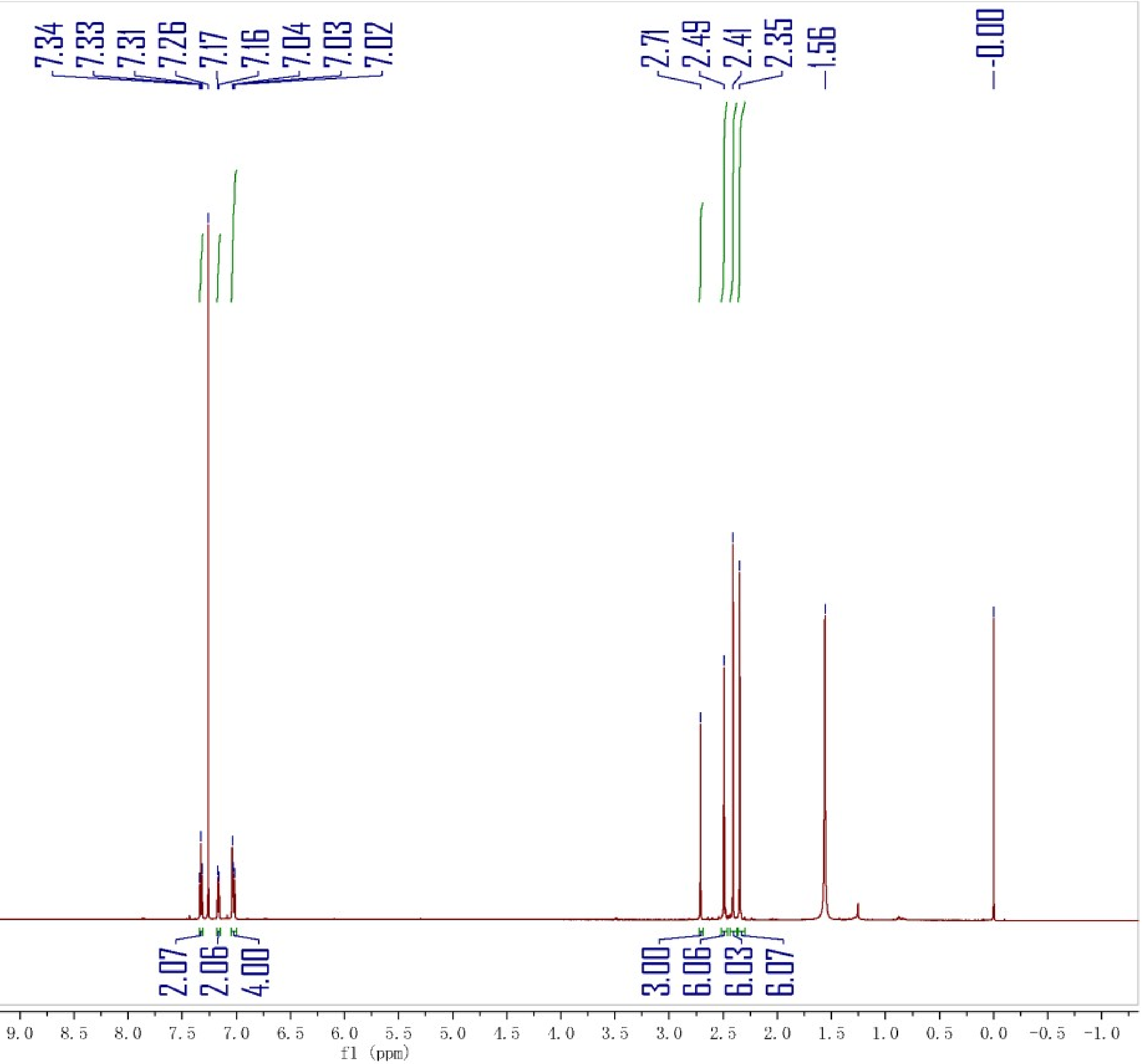

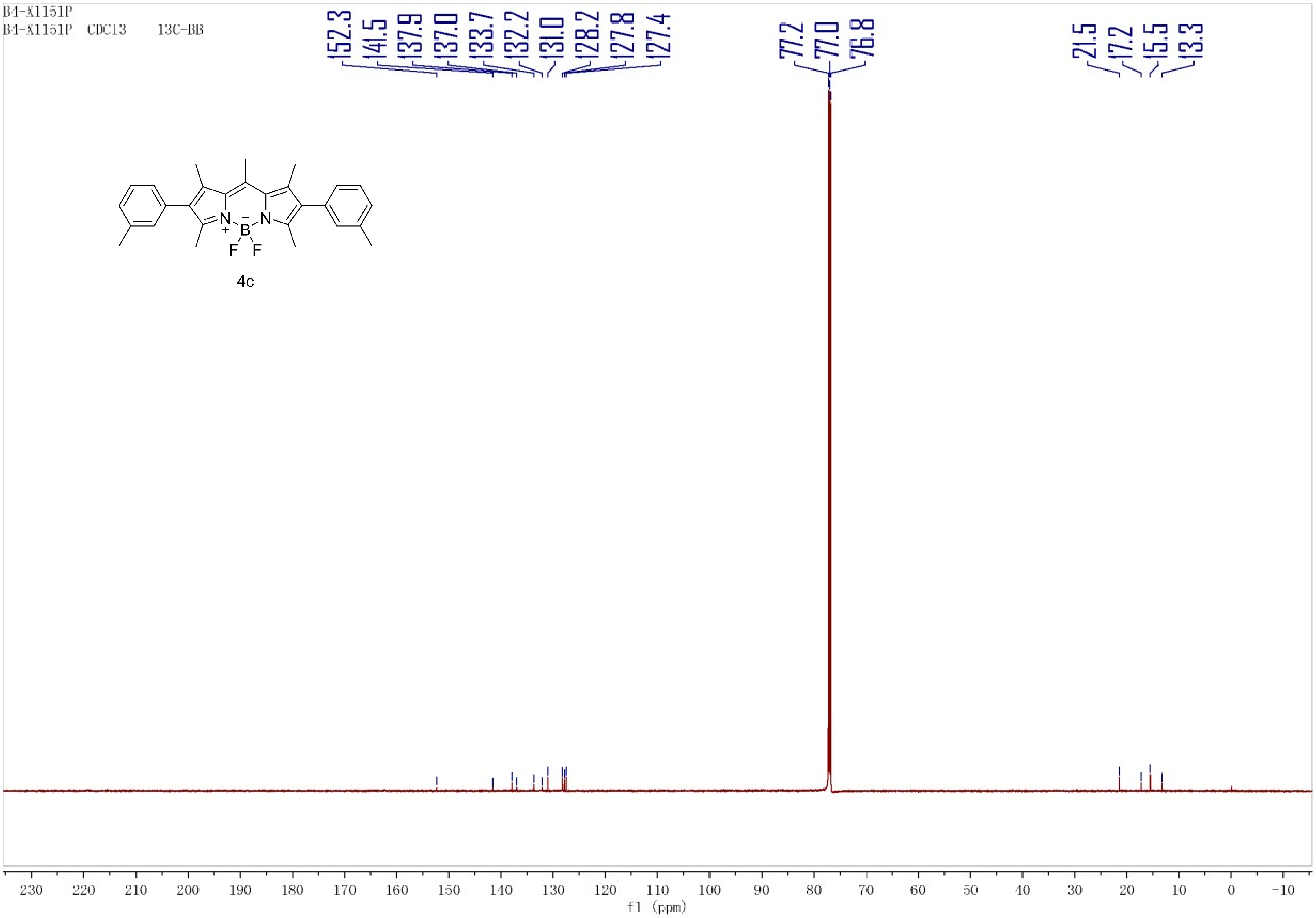
B4-X1151P
B4-X1151P CDCl3 13C-BB
152.3
141.5
137.9
137.0
133.7
132.2
131.0
128.2
127.8
127.4
77.2
77.0
76.8
21.5
17.2
15.5
13.3
N
N
B
F
F
4c
230 220 210 200 190 180 170 160 150 140 130 120 110 100 90 80 70 60 50 40 30 20 10 0 -10
f1 (ppm)

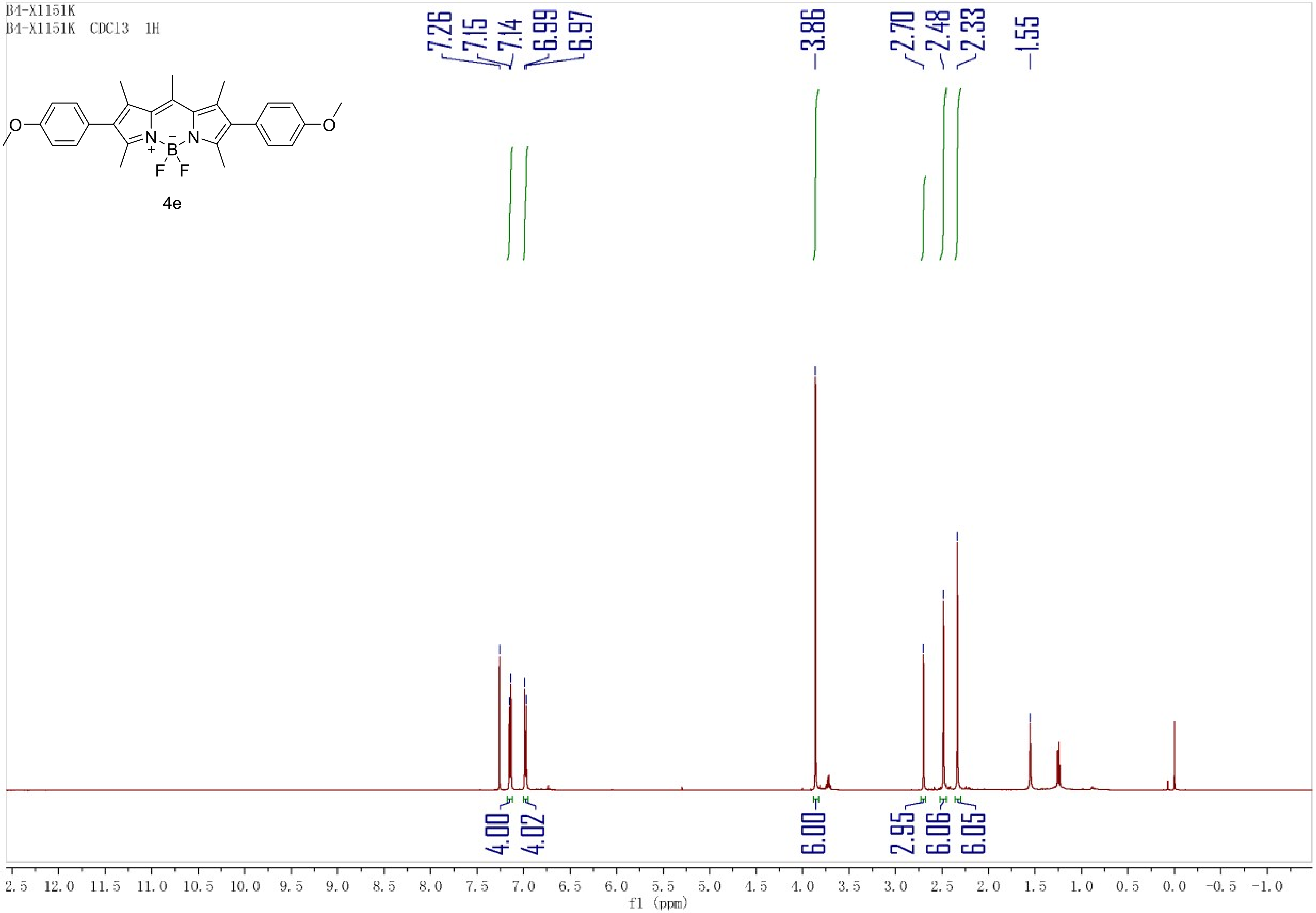

B4-X1151K
B4-X1151K CDCl3 1H
4e
7.26
7.15
7.14
6.99
6.97
3.86
2.70
2.48
2.33
1.55
4.00
4.02
6.00
2.95
6.06
6.05
f1 (ppm)

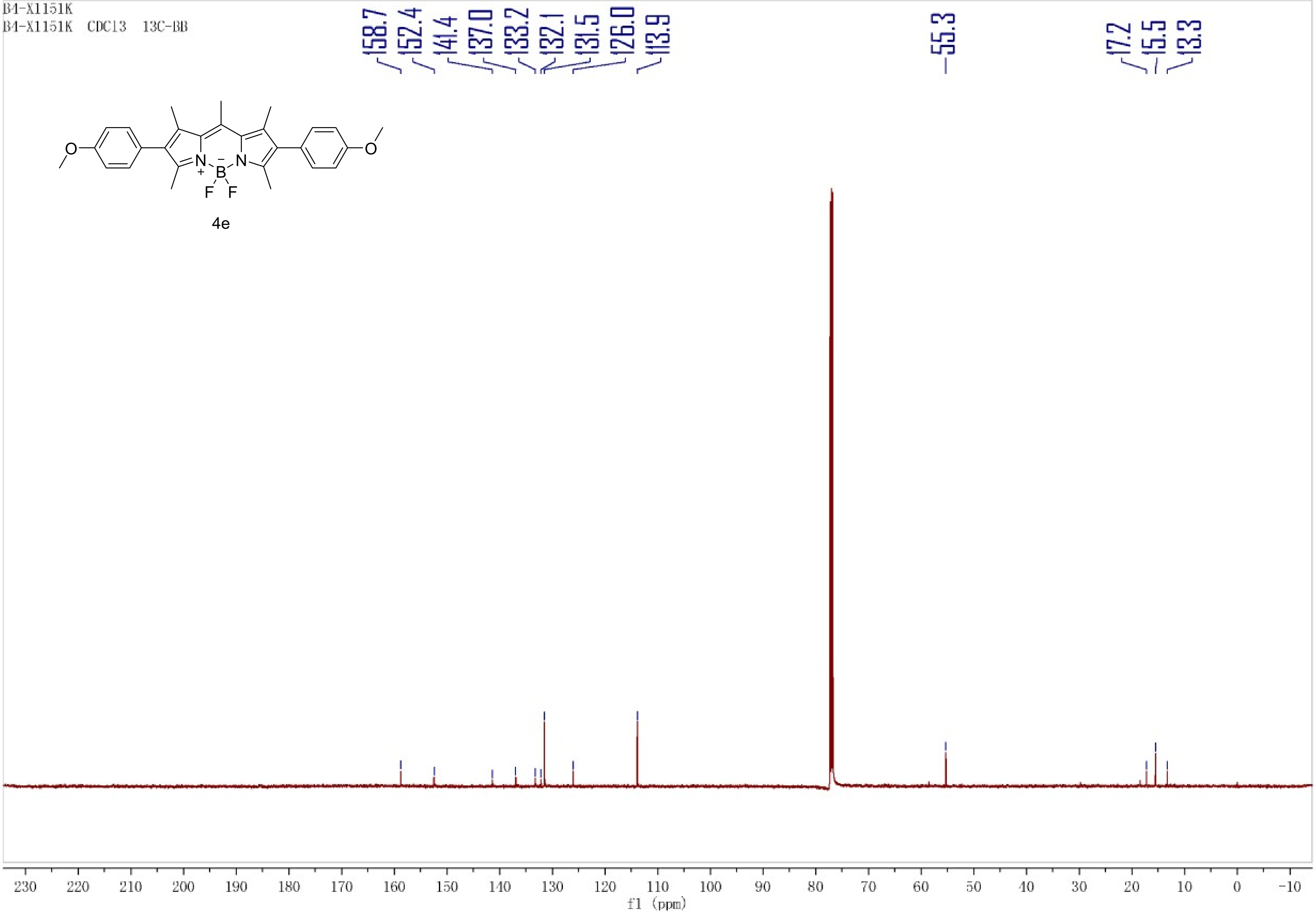

B4-X1151K
B4-X1151K CDCl3 13C-BB
158.7
152.4
141.4
137.0
133.2
132.1
131.5
126.0
113.9
55.3
17.2
15.5
13.3
O
O
N
N
B
F
F
4e
230 220 210 200 190 180 170 160 150 140 130 120 110 100 90 80 70 60 50 40 30 20 10 0 -10
f1 (ppm)

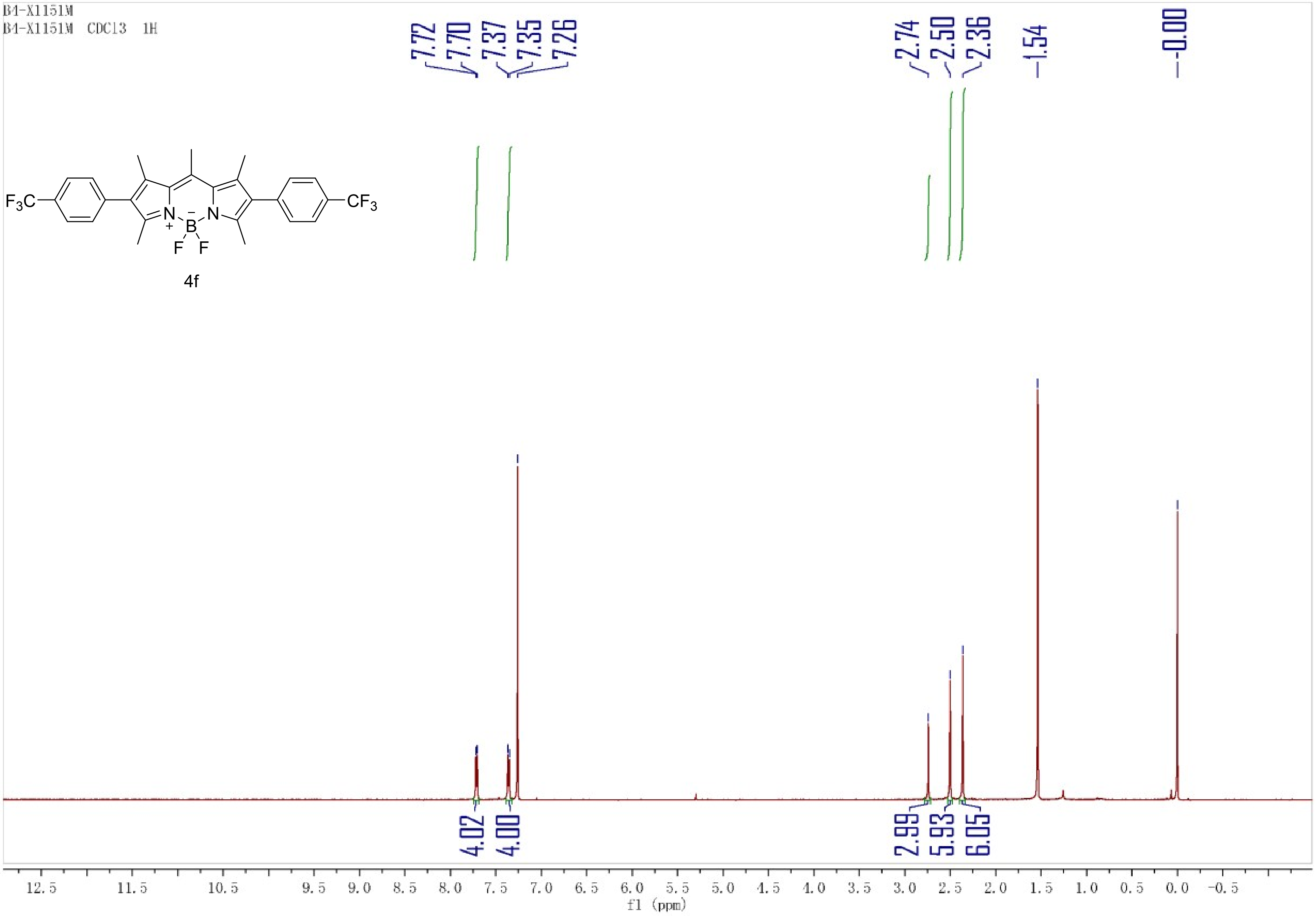
B4-X1151M
B4-X1151M CDCl3 1H
7.72
7.70
7.37
7.35
7.26
2.74
2.50
2.36
1.54
0.00
F3C
CF3
N
B
N
F F
4.02
4.00
2.99
5.93
6.05
12.5 11.5 10.5 9.5 9.0 8.5 8.0 7.5 7.0 6.5 6.0 5.5 5.0 4.5 4.0 3.5 3.0 2.5 2.0 1.5 1.0 0.5 0.0 -0.5
f1 (ppm)

4f

B4-X1151M
B4-X1151M CDCl3 13C-BB

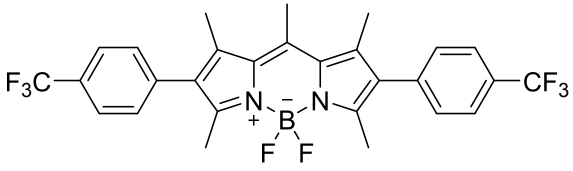

4f

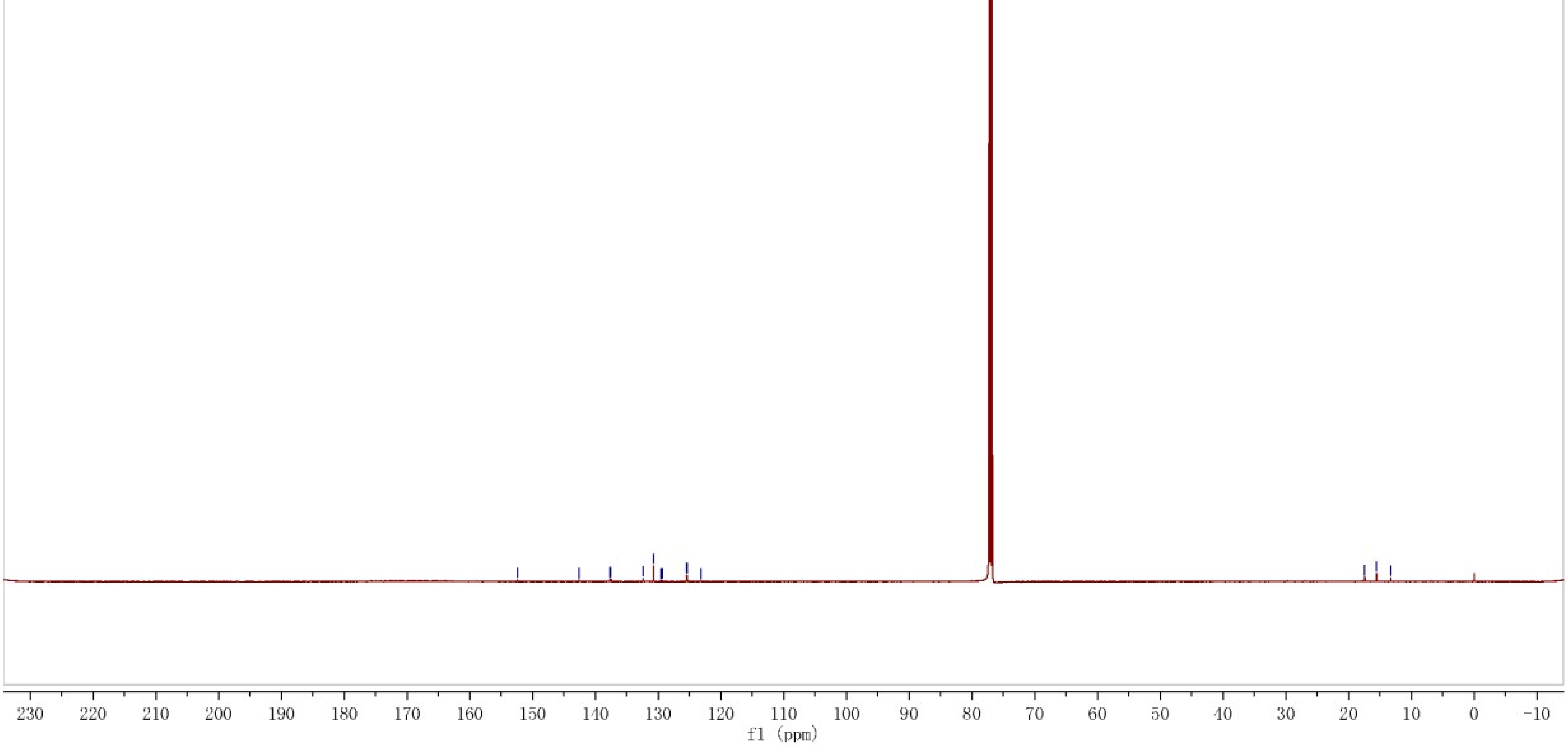

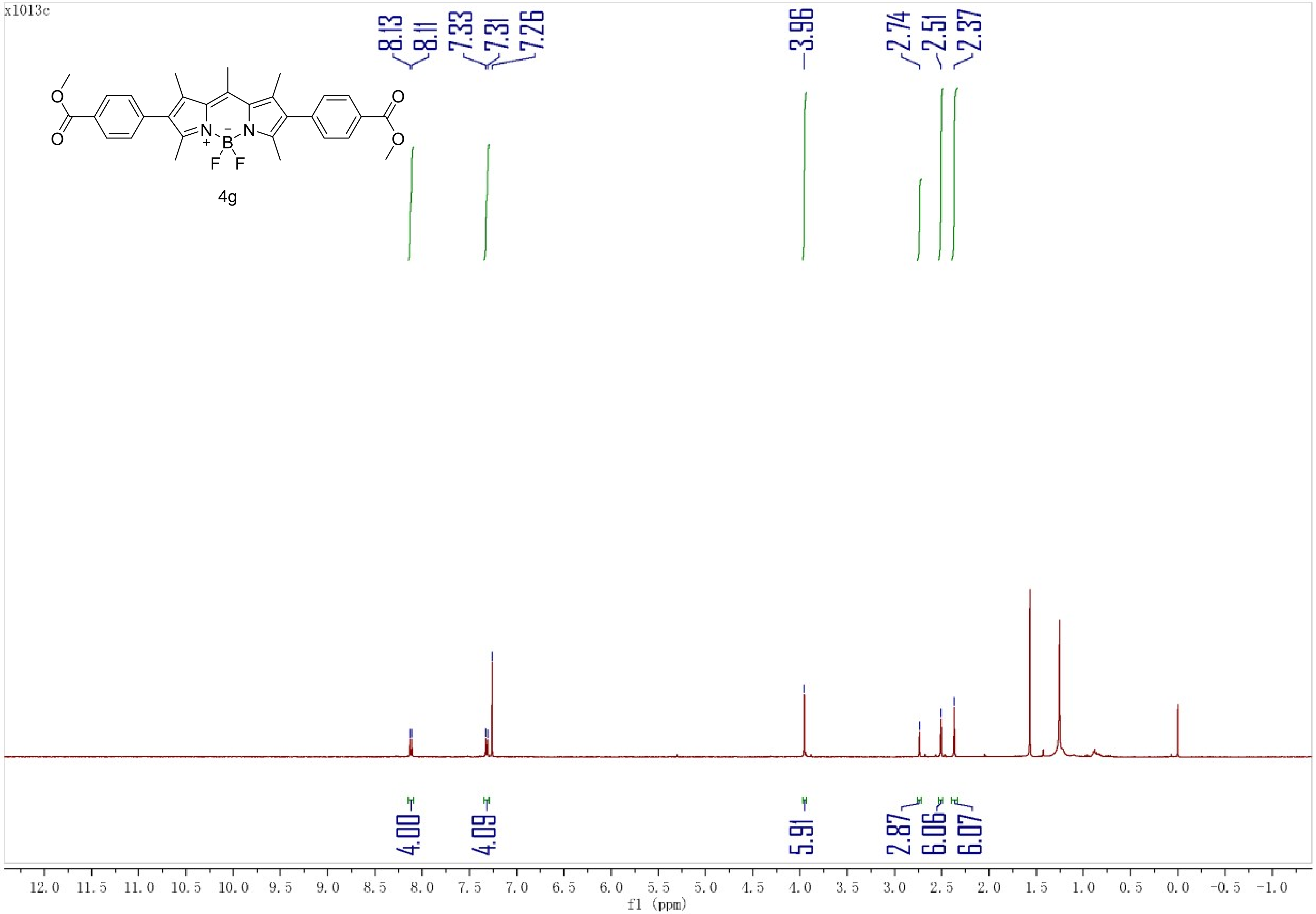
x1013c
8.13
8.11
7.33
7.31
7.26
3.96
2.74
2.51
2.37
4g
4.00
4.09
5.91
2.87
6.06
6.07
12.0 11.5 11.0 10.5 10.0 9.5 9.0 8.5 8.0 7.5 7.0 6.5 6.0 5.5 5.0 4.5 4.0 3.5 3.0 2.5 2.0 1.5 1.0 0.5 0.0 -0.5 -1.0
f1 (ppm)

B4-X1151H
B4-X1151H CDCl3 13C-BB

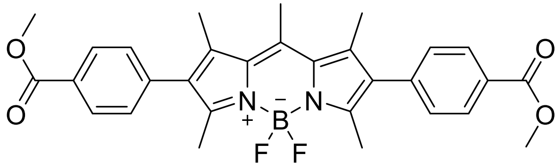

4g

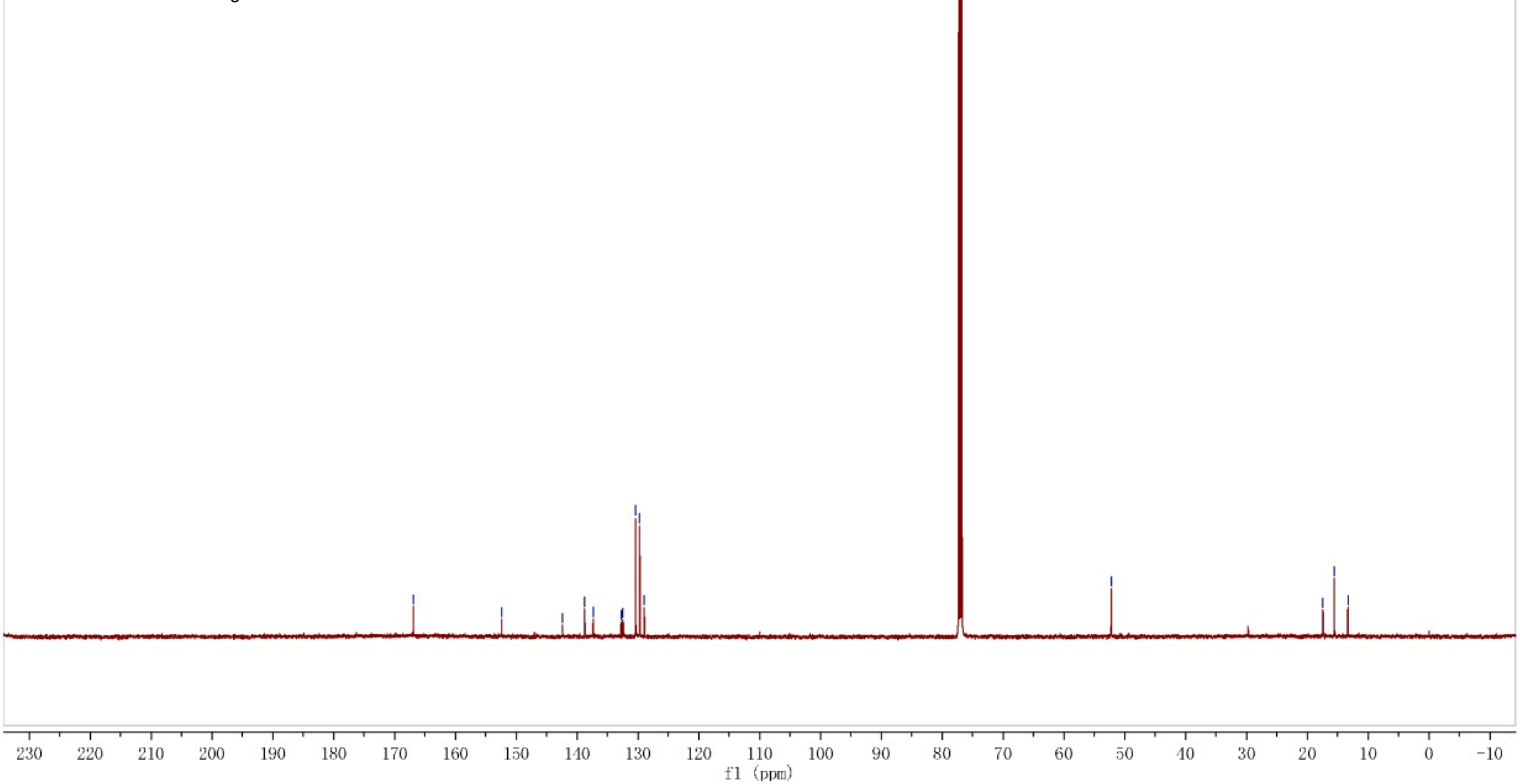

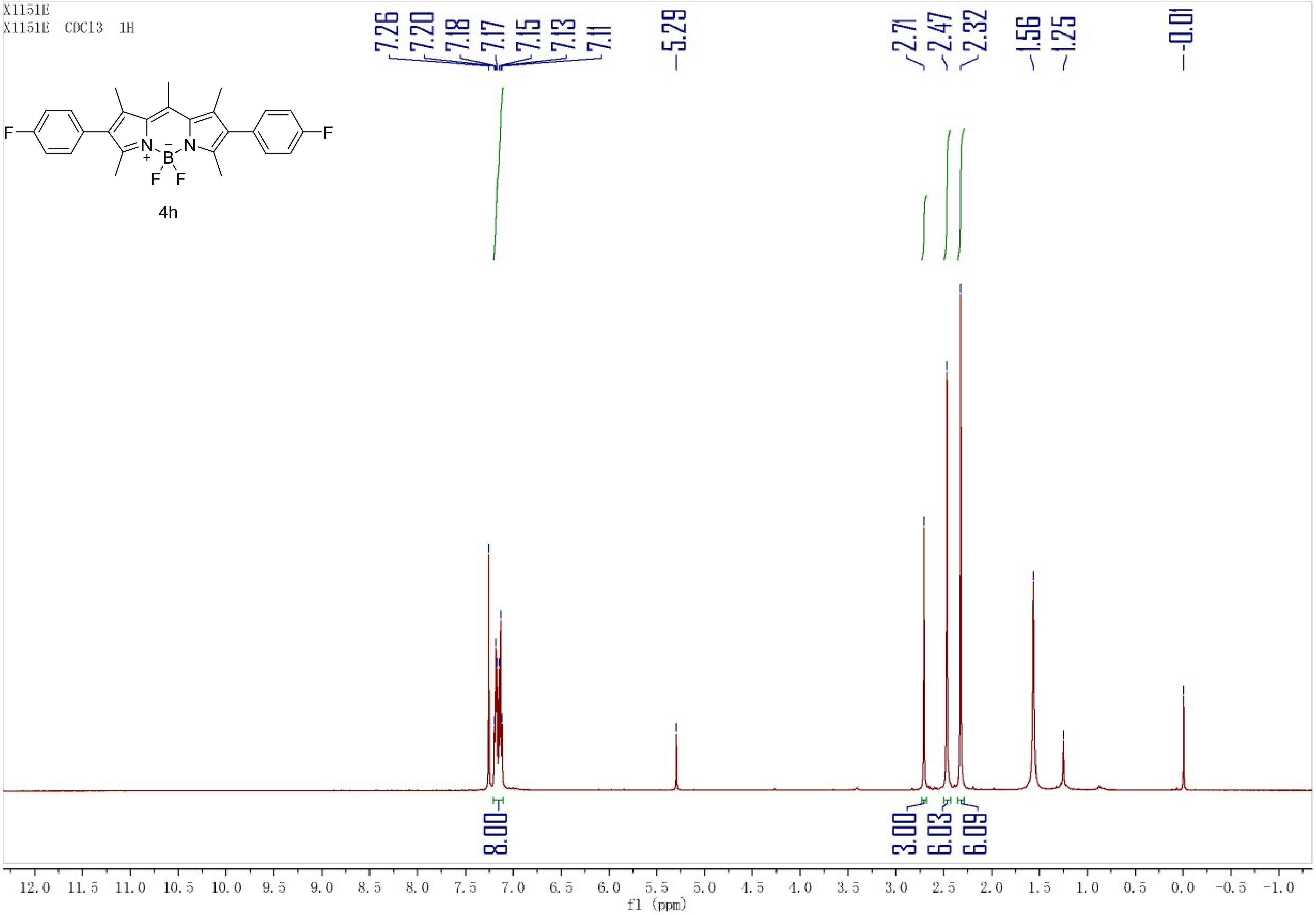
X1151E
X1151E CDCl3 1H
7.26
7.20
7.18
7.17
7.15
7.13
7.11
5.29
2.71
2.47
2.32
1.56
1.25
-0.01
F
F
N
B
N
F
F
4h
8.00
3.00
6.03
6.09
12.0 11.5 11.0 10.5 10.0 9.5 9.0 8.5 8.0 7.5 7.0 6.5 6.0 5.5 5.0 4.5 4.0 3.5 3.0 2.5 2.0 1.5 1.0 0.5 0.0 -0.5 -1.0
f1 (ppm)

X1151E
X1151E CDCl3 13C-BB

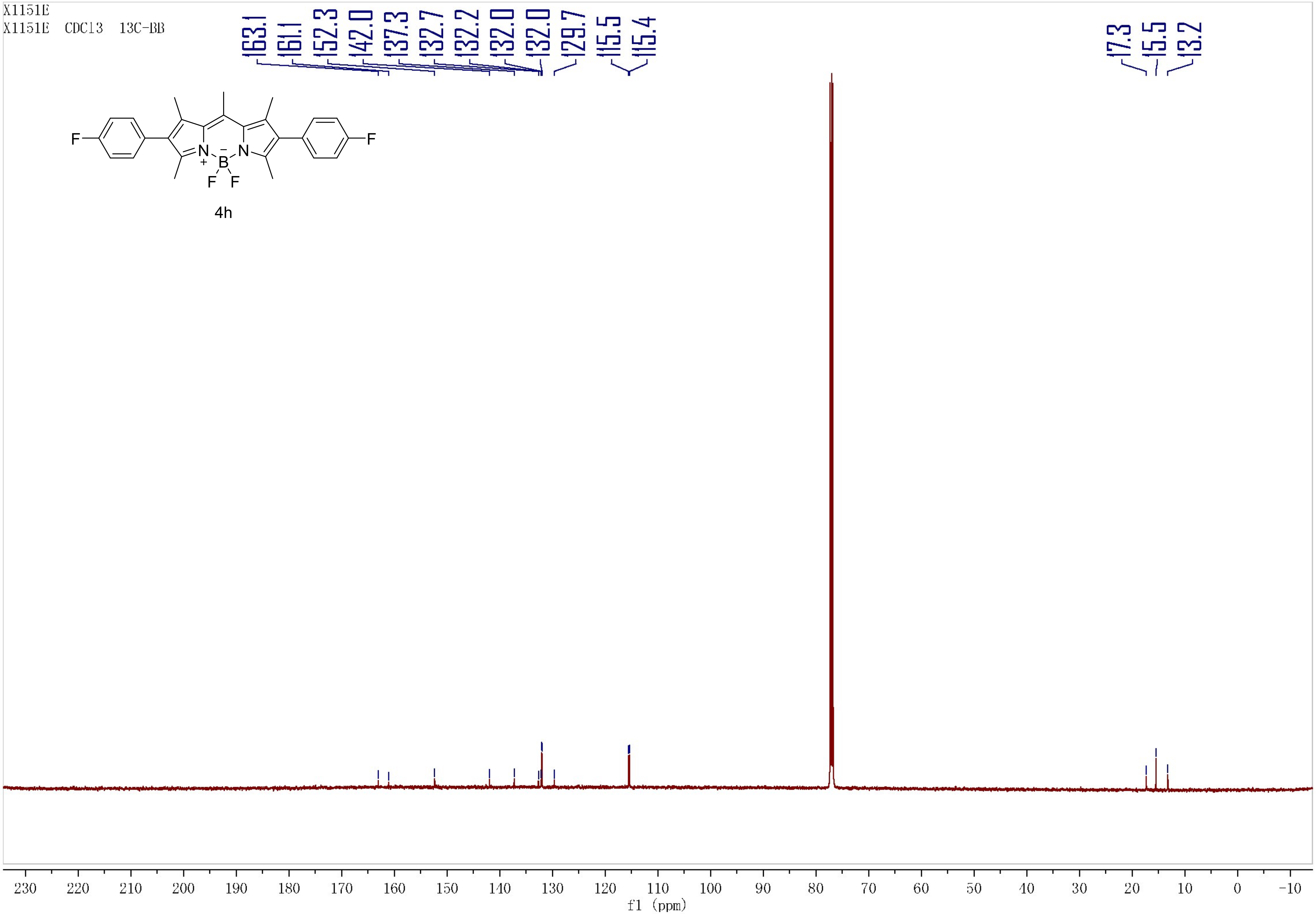

B4-X1151J
B4-X1151J CDCl3 1H

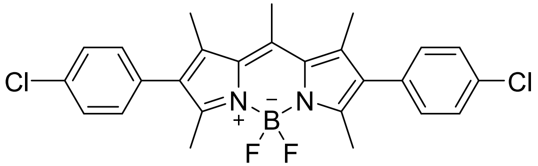

4i

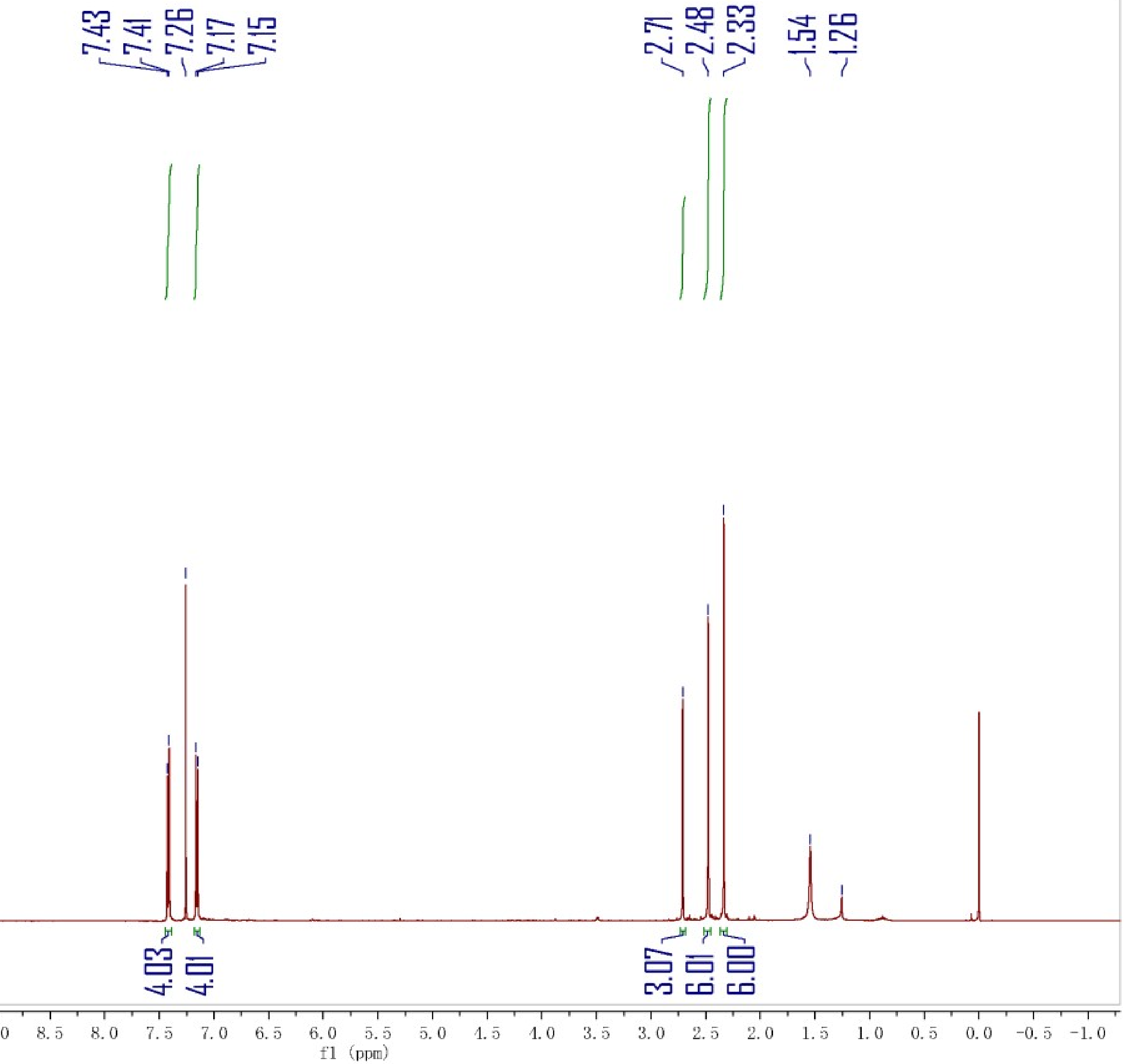

B4-X1151J
B4-X1151J CDCl3 13C-BB

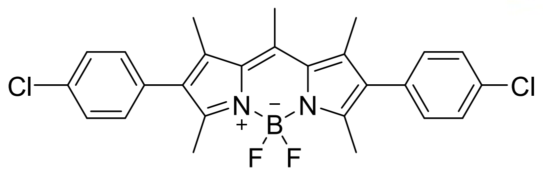

4i

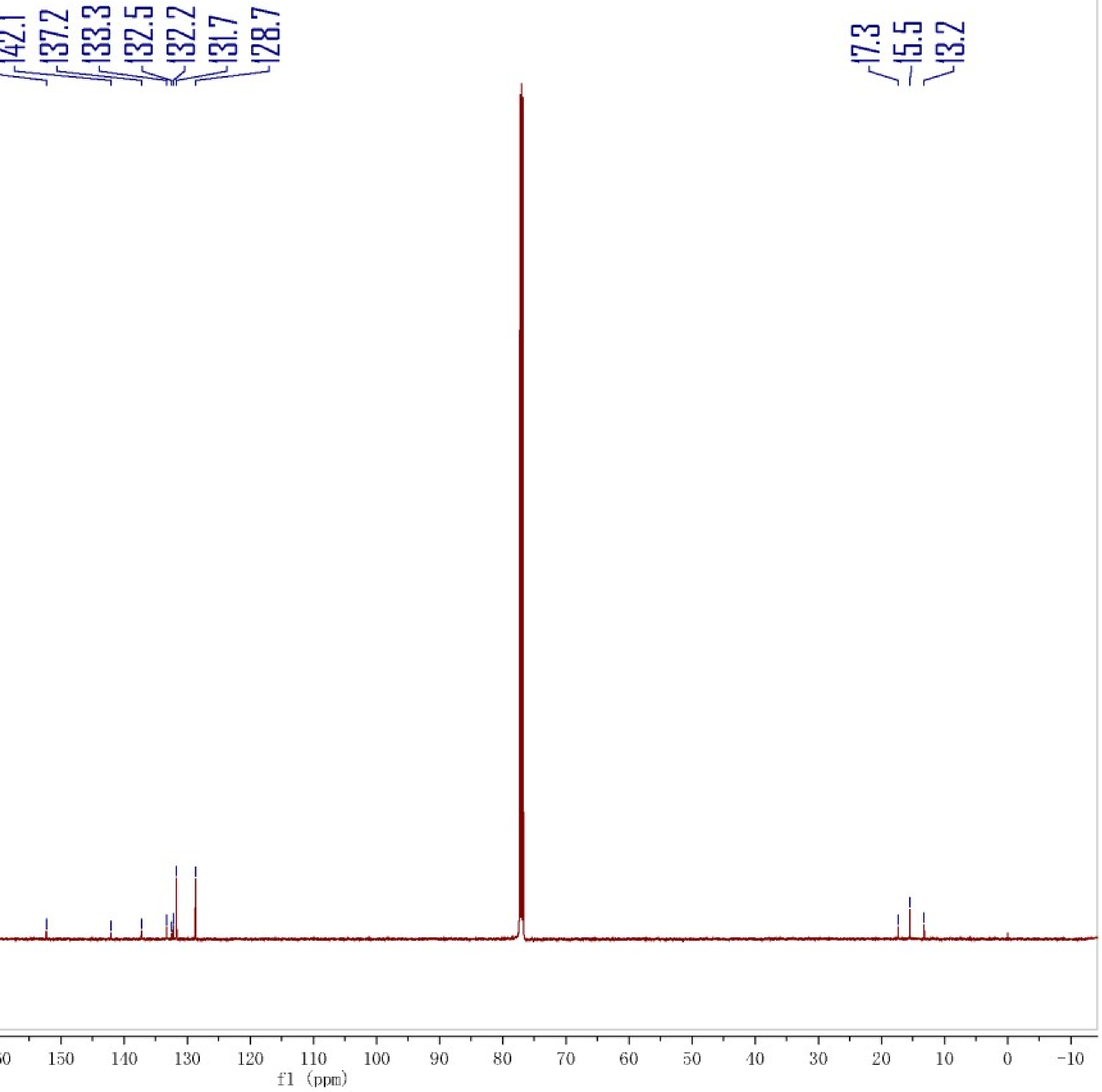

B4-X1151L
B4-X1151L CDCl3 1H

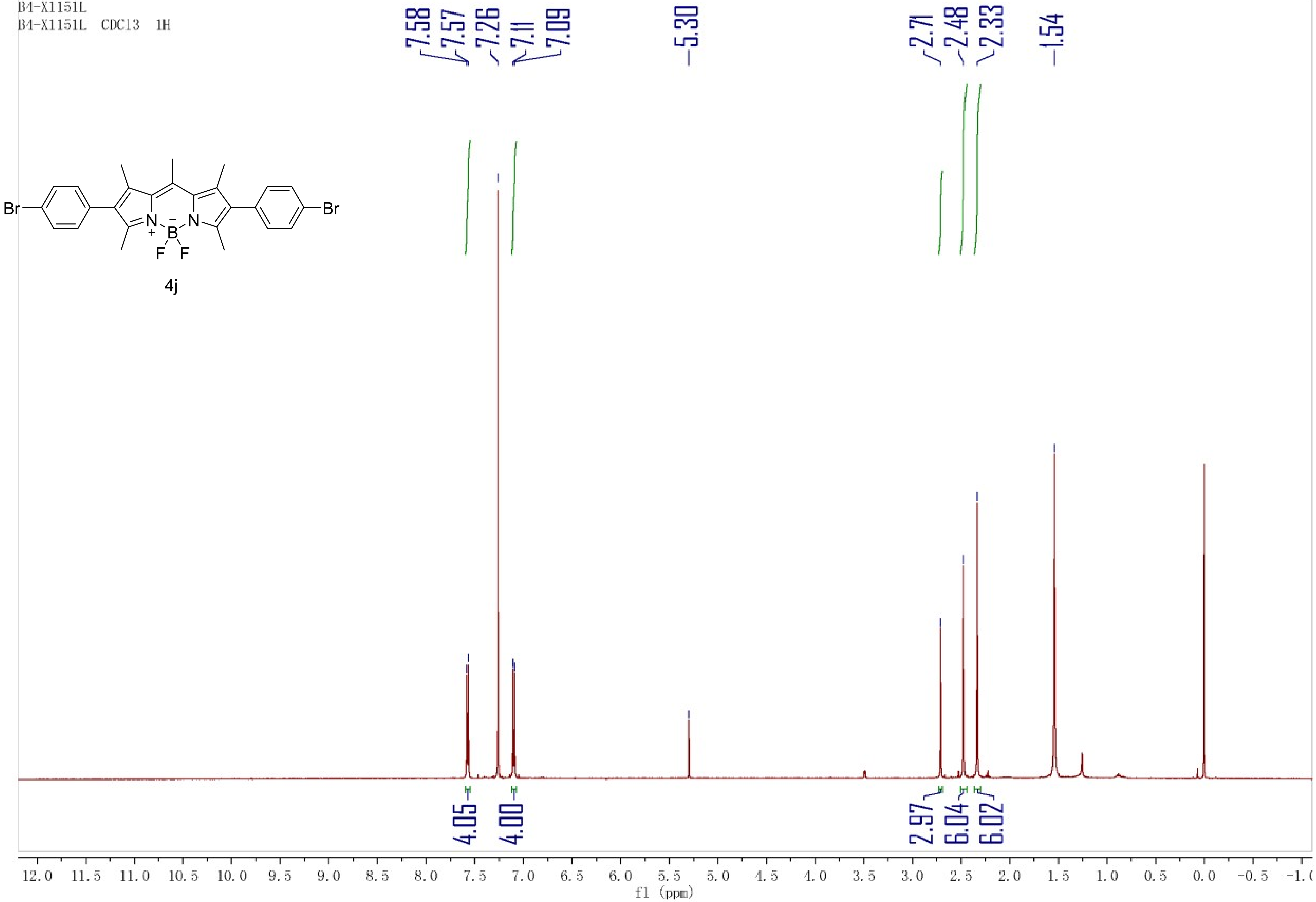

4j

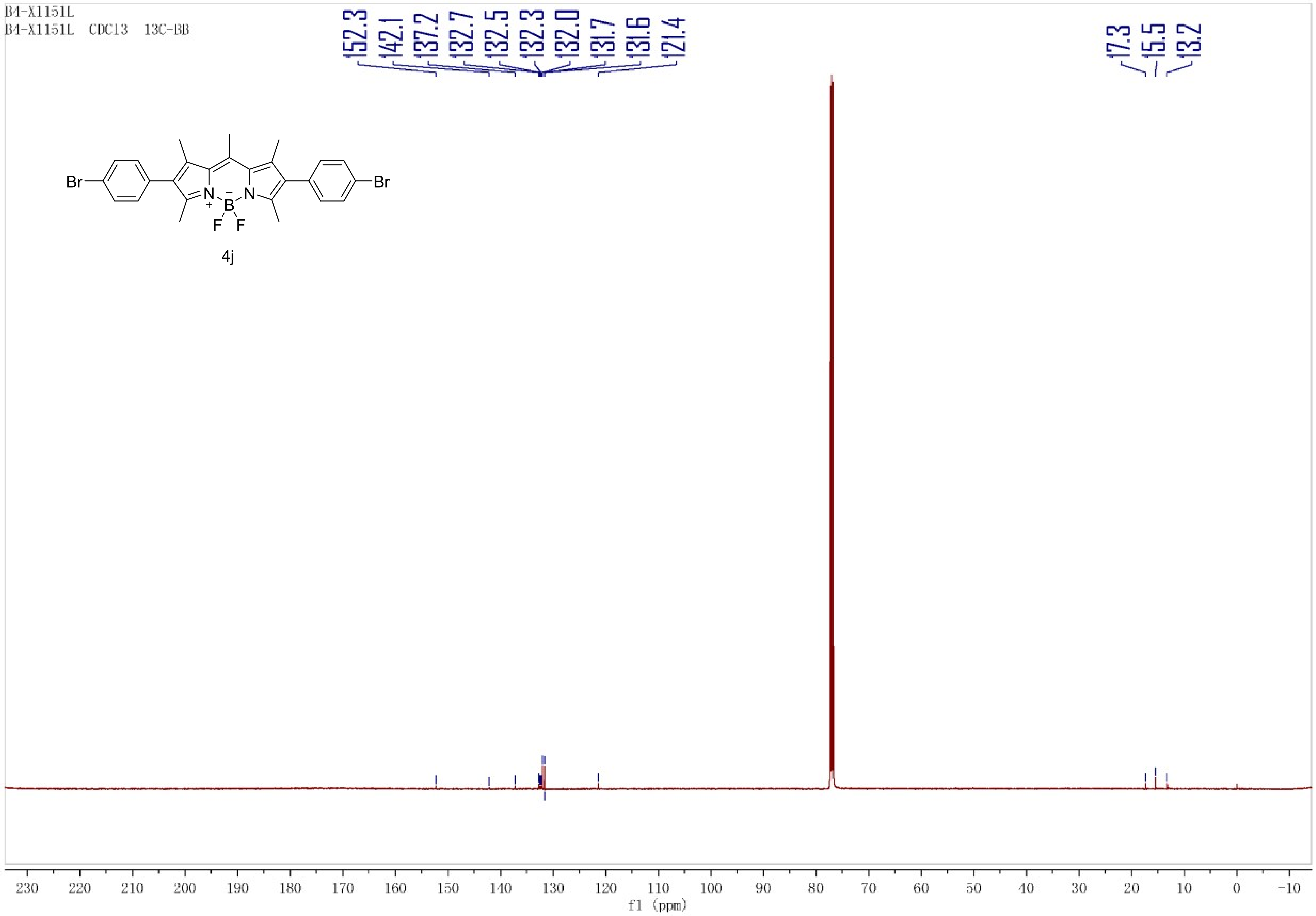
B4-X1151L
B4-X1151L CDCl3 13C-BB
152.3
142.1
137.2
132.7
132.5
132.3
132.0
131.7
131.6
121.4
17.3
15.5
13.2
Br
Br
N
B
N
F
F
4j
230 220 210 200 190 180 170 160 150 140 130 120 110 100 90 80 70 60 50 40 30 20 10 0 -10
f1 (ppm)

B4-X1151Q
B4-X1151Q CDCl3 1H

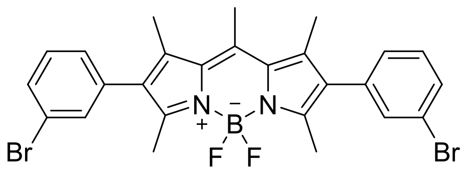

4k

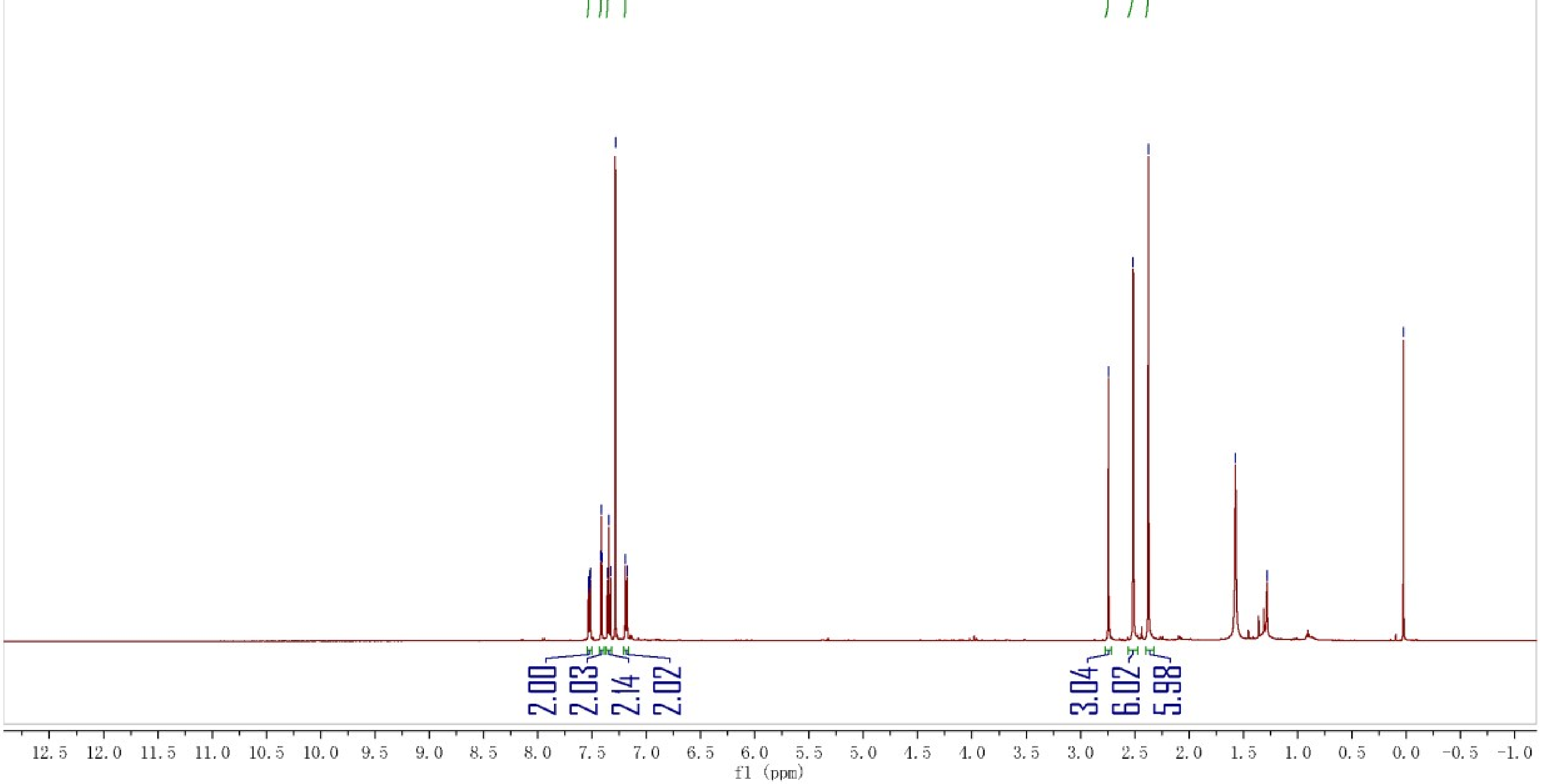

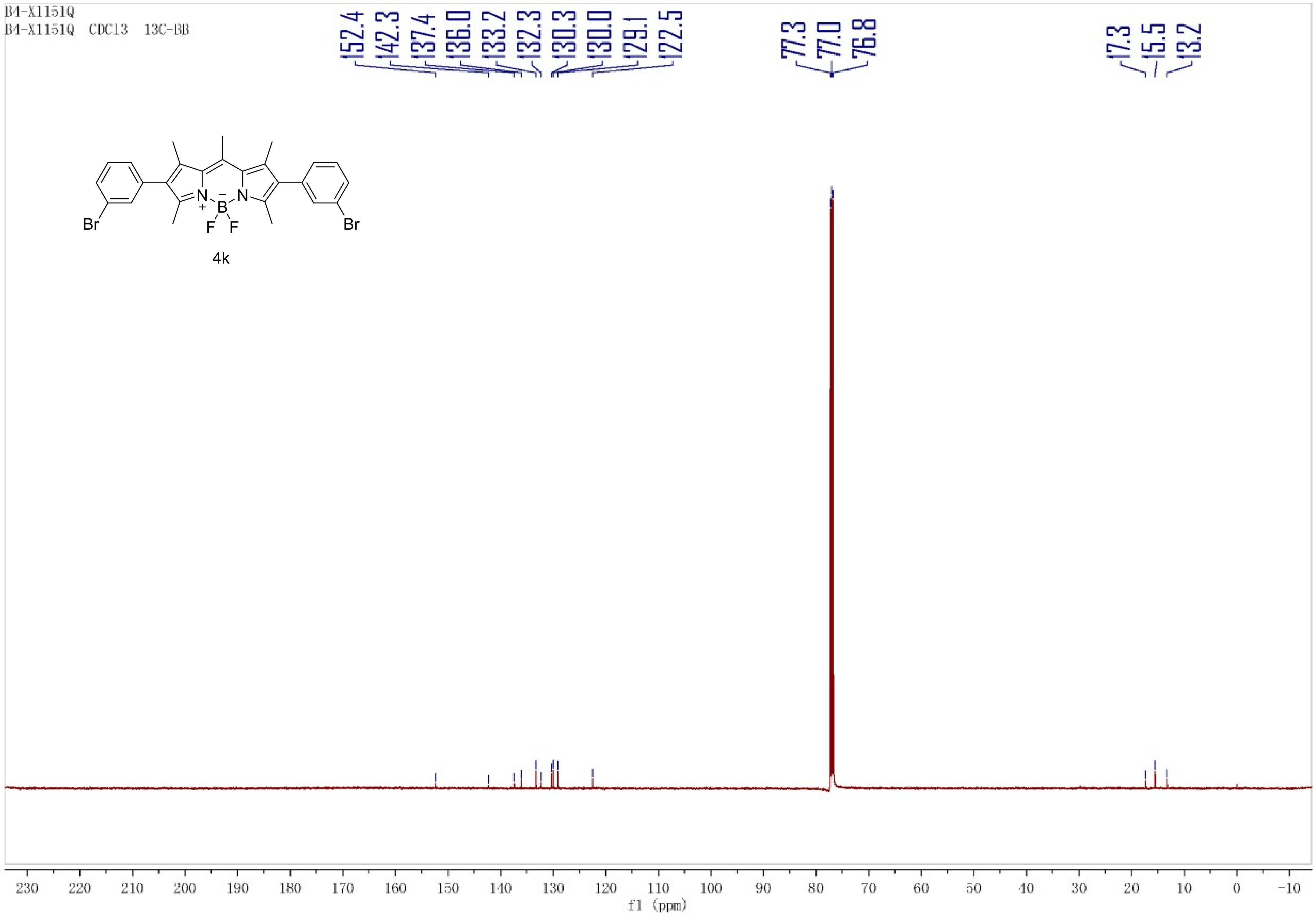
B4-X1151Q
B4-X1151Q CDCl3 13C-BB
152.4
142.3
137.4
136.0
133.2
132.3
130.3
130.0
129.1
122.5
77.3
77.0
76.8
17.3
15.5
13.2
Br
N
B
N
F
F
Br
4k
230 220 210 200 190 180 170 160 150 140 130 120 110 100 90 80 70 60 50 40 30 20 10 0 -10
f1 (ppm)

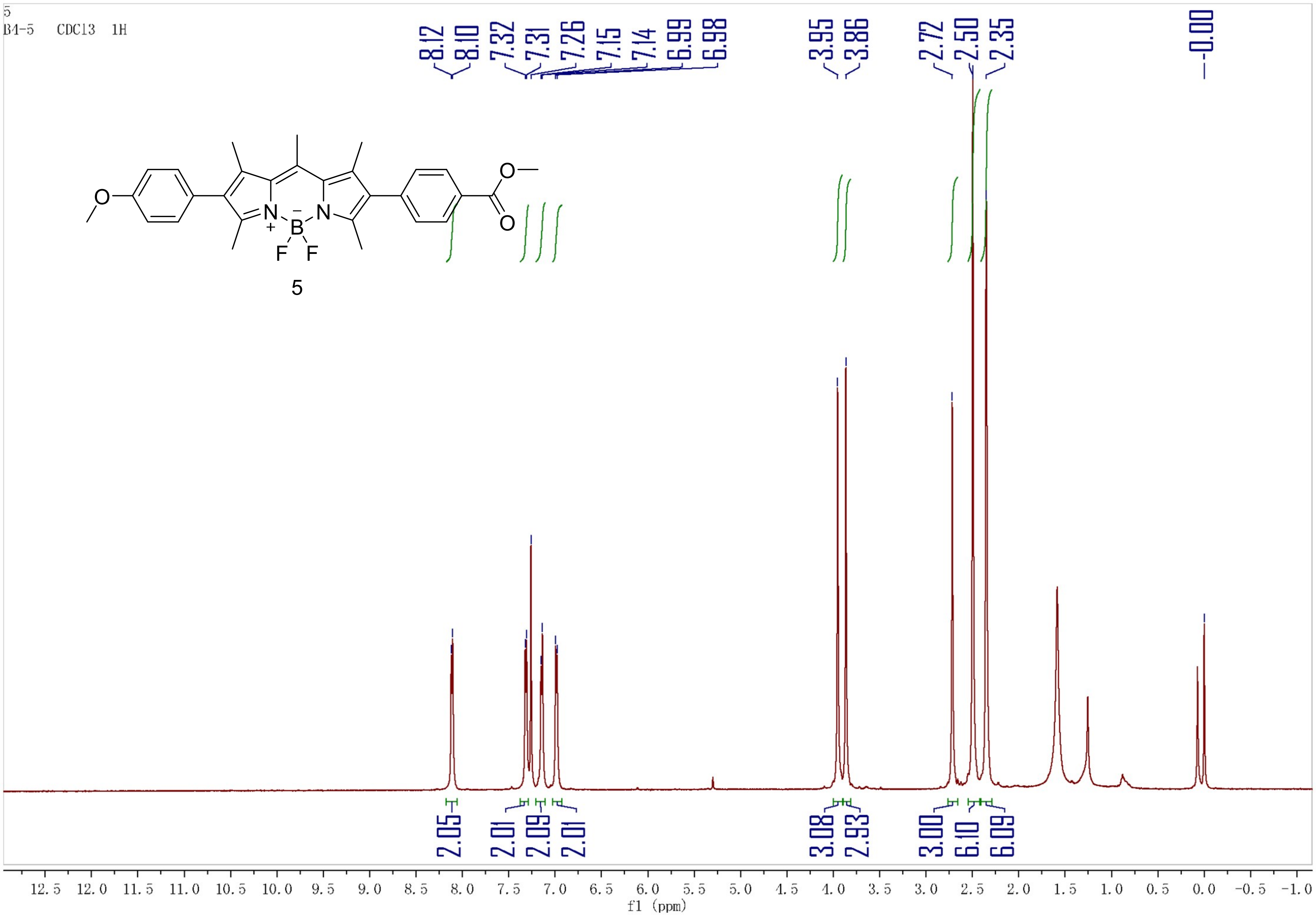
5
B4-5 CDCl3 1H
8.12
8.10
7.32
7.31
7.26
7.15
7.14
6.99
6.98
3.95
3.86
2.72
2.50
2.35
-0.00
2.05
2.01
2.09
2.01
3.08
2.93
3.00
6.10
6.09
12.5 12.0 11.5 11.0 10.5 10.0 9.5 9.0 8.5 8.0 7.5 7.0 6.5 6.0 5.5 5.0 4.5 4.0 3.5 3.0 2.5 2.0 1.5 1.0 0.5 0.0 -0.5 -1.0
f1 (ppm)
5

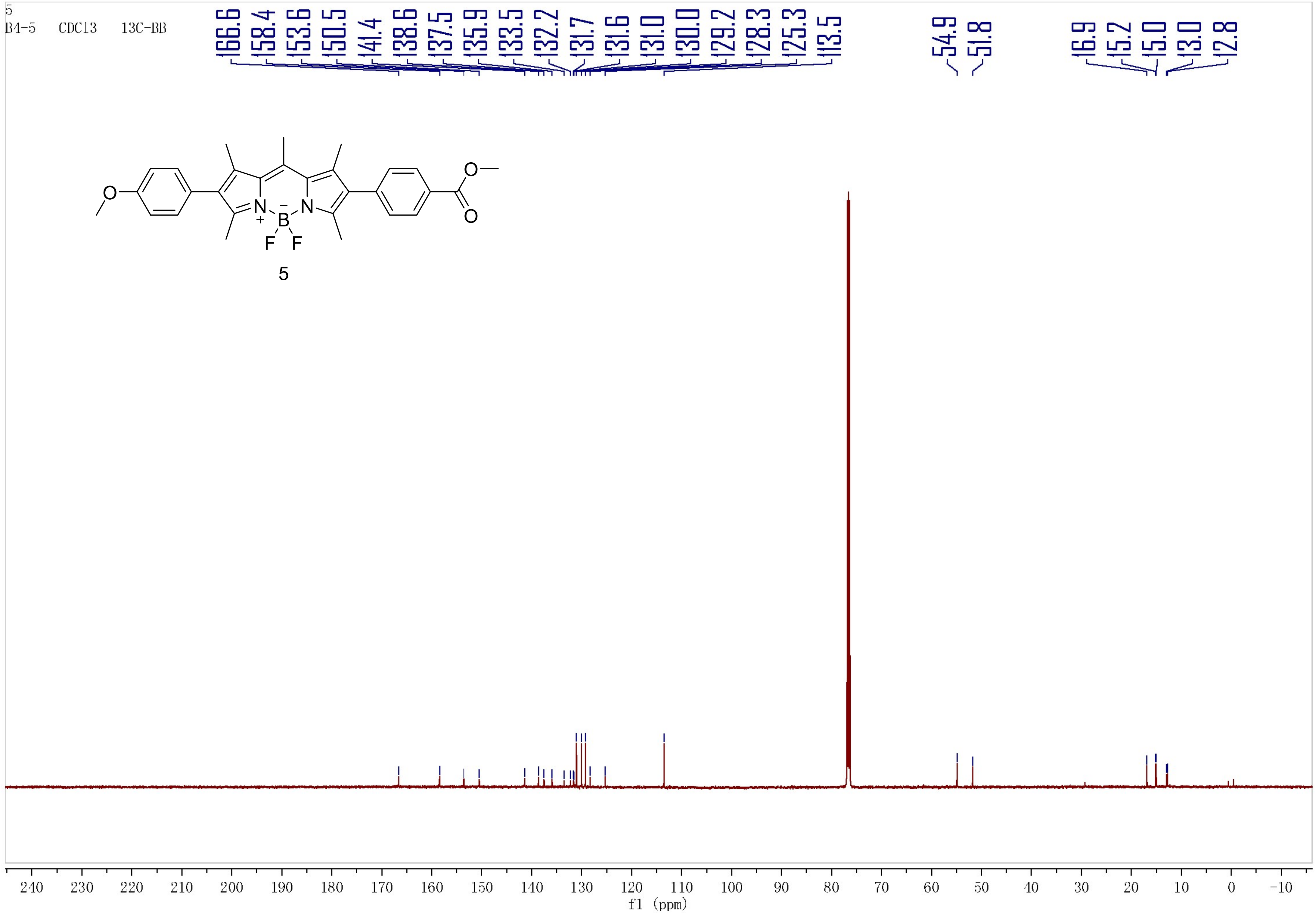

5
B4-5 CDCl3 13C-BB
166.6
158.4
153.6
150.5
141.4
138.6
137.5
135.9
133.5
132.2
131.7
131.6
131.0
130.0
129.2
128.3
125.3
113.5
54.9
51.8
16.9
15.2
15.0
13.0
12.8
O
N
B
N
F F
O
O
5
240 230 220 210 200 190 180 170 160 150 140 130 120 110 100 90 80 70 60 50 40 30 20 10 0 −10
f1 (ppm)

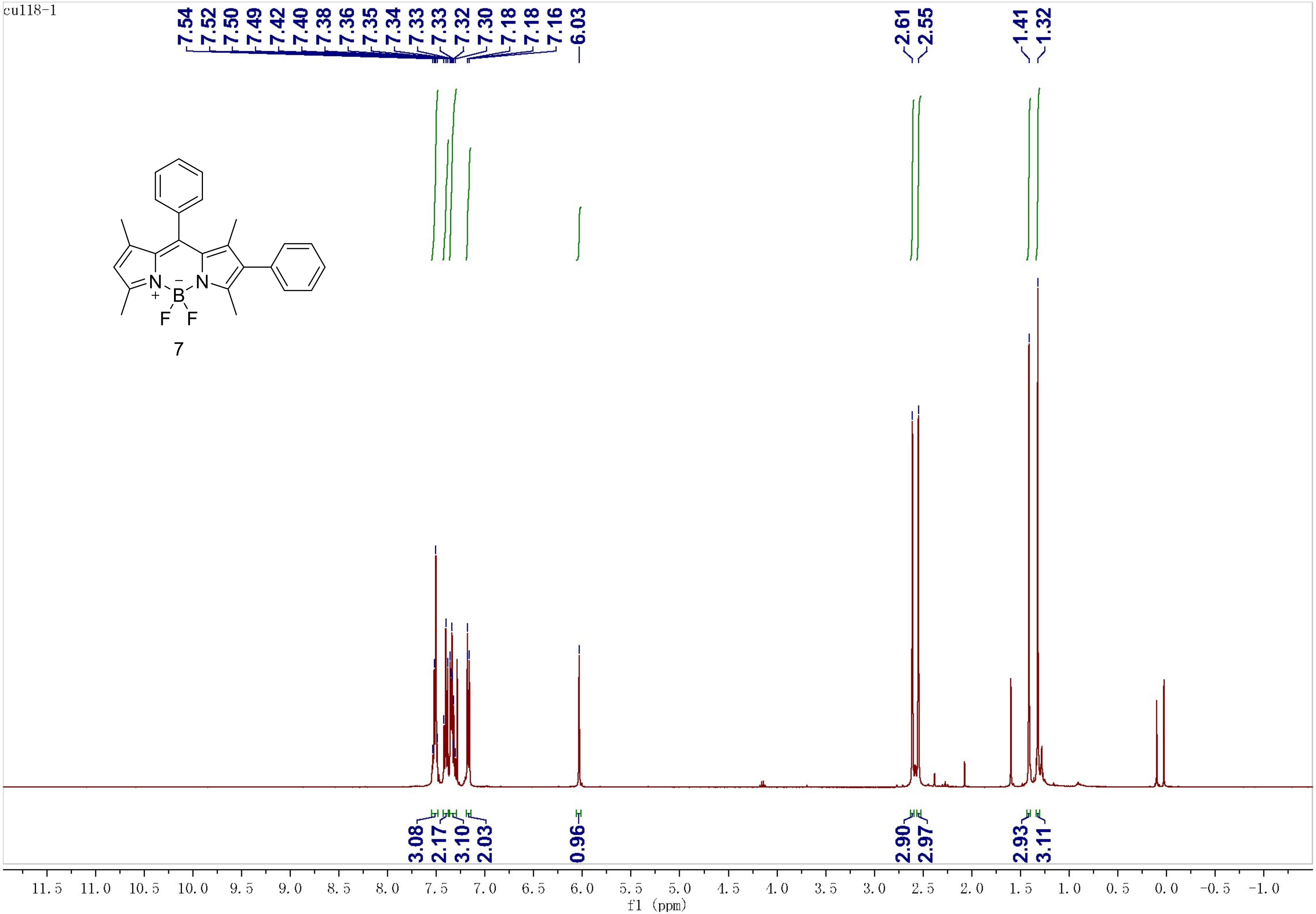

cu118-1
7.54 7.52 7.50 7.49 7.42 7.40 7.38 7.36 7.35 7.34 7.33 7.33 7.32 7.30 7.18 7.18 7.16
6.03
2.61 2.55
1.41 1.32
3.08 2.17 3.10 2.03
0.96
2.90 2.97
2.93 3.11
N
B
N
F F
7
11.5 11.0 10.5 10.0 9.5 9.0 8.5 8.0 7.5 7.0 6.5 6.0 5.5 5.0 4.5 4.0 3.5 3.0 2.5 2.0 1.5 1.0 0.5 0.0 -0.5 -1.0
f1 (ppm)

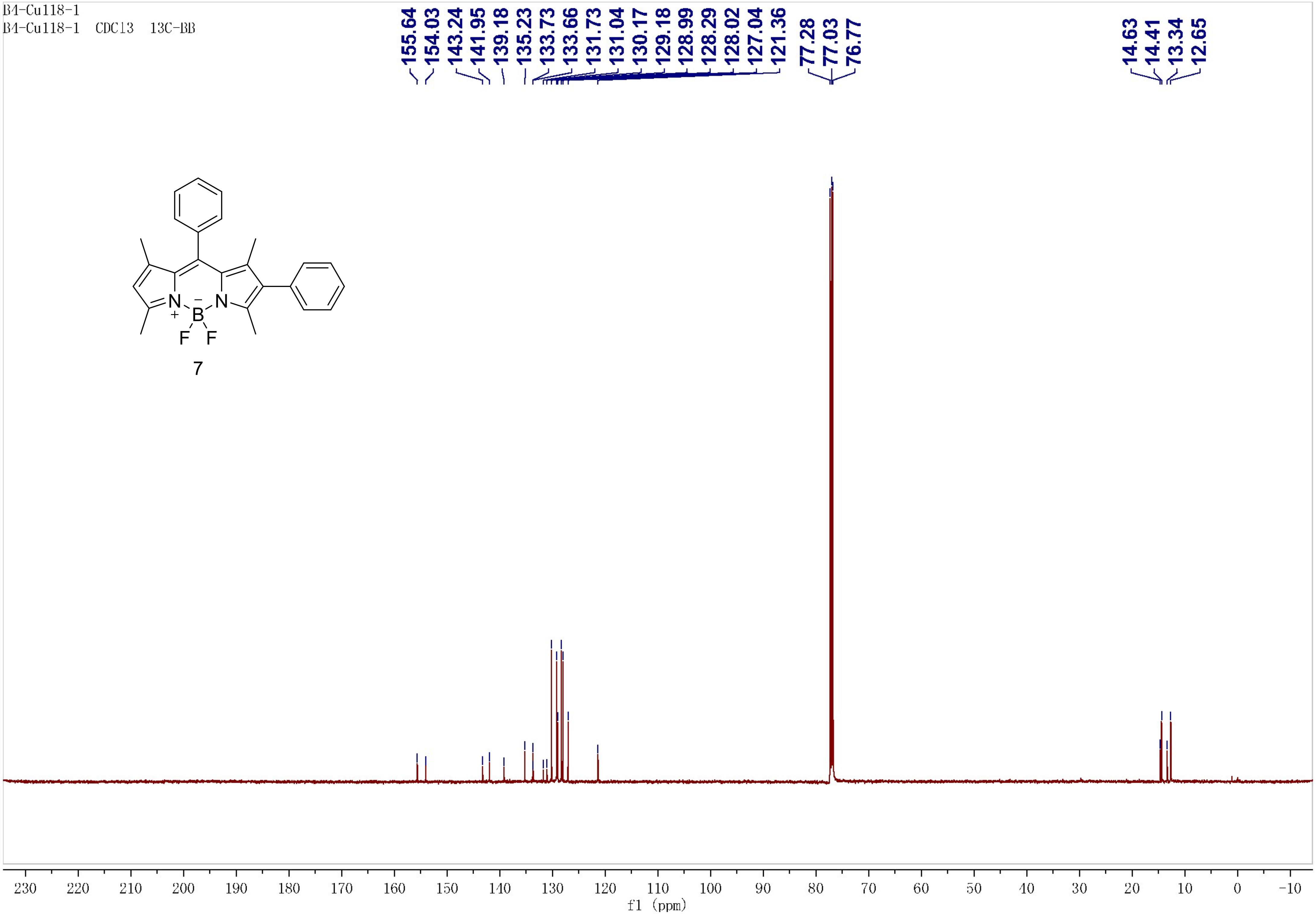
B4-Cu118-1
B4-Cu118-1 CDCl3 13C-BB
155.64
154.03
143.24
141.95
139.18
135.23
133.73
133.66
131.73
131.04
130.17
129.18
128.99
128.29
128.02
127.04
121.36
77.28
77.03
76.77
14.63
14.41
13.34
12.65
N
B
N
F
F
7
230 220 210 200 190 180 170 160 150 140 130 120 110 100 90 80 70 60 50 40 30 20 10 0 -10
f1 (ppm)

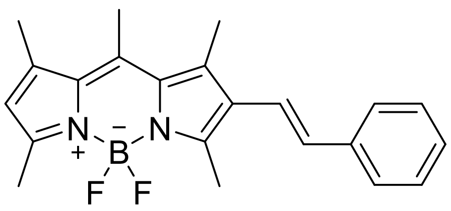

9a

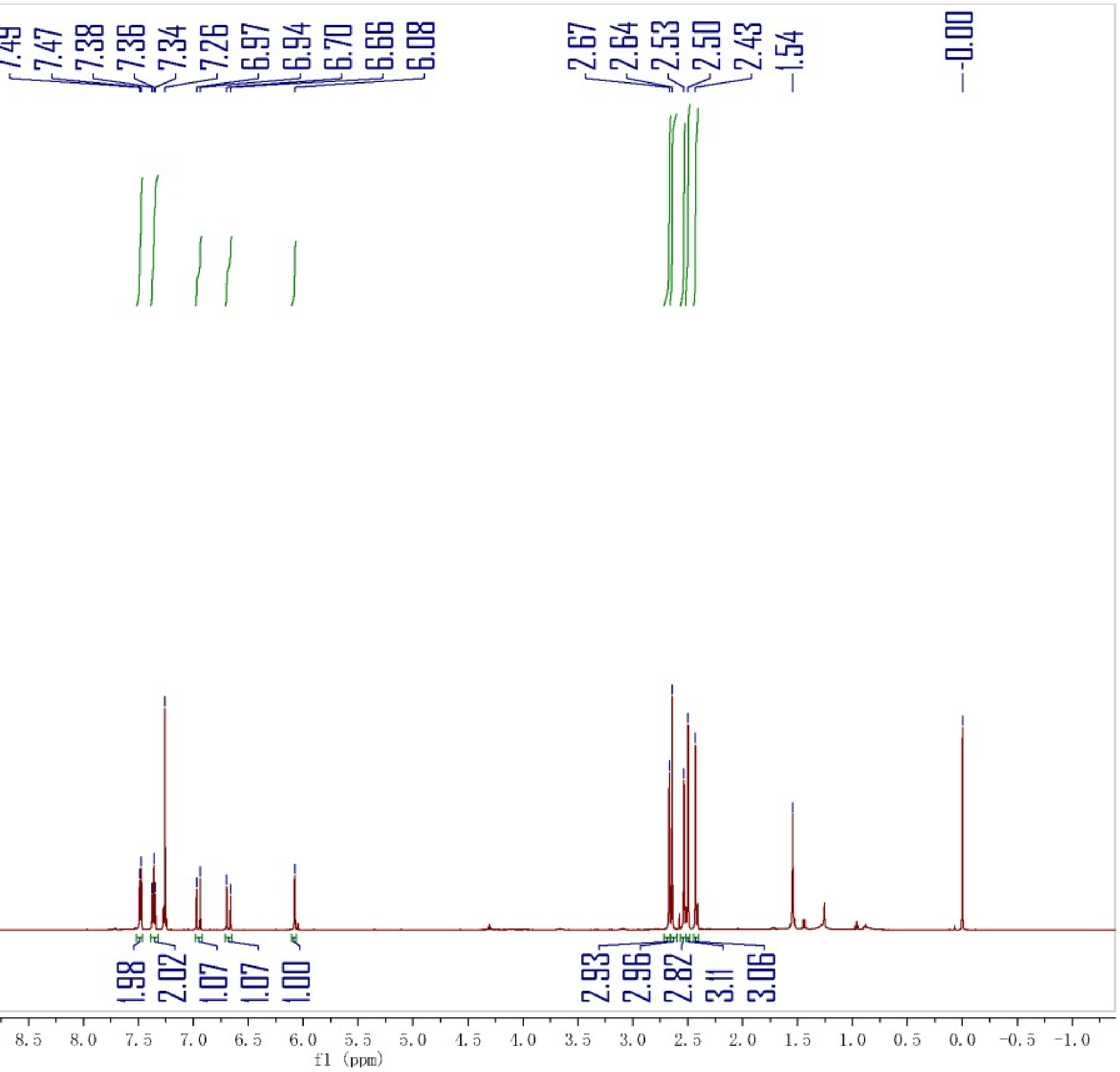

B4-X1152D-20170524
B4-X1152D-20170524 CDCl3 1H
7.49
7.47
7.38
7.36
7.34
7.26
6.97
6.94
6.70
6.66
6.08
2.67
2.64
2.53
2.50
2.43
1.54
0.00
1.98
2.02
1.07
1.07
1.00
2.93
2.96
2.82
3.11
3.06
2.5 12.0 11.5 11.0 10.5 10.0 9.5 9.0 8.5 8.0 7.5 7.0 6.5 6.0 5.5 5.0 4.5 4.0 3.5 3.0 2.5 2.0 1.5 1.0 0.5 0.0 -0.5 -1.0
f1 (ppm)

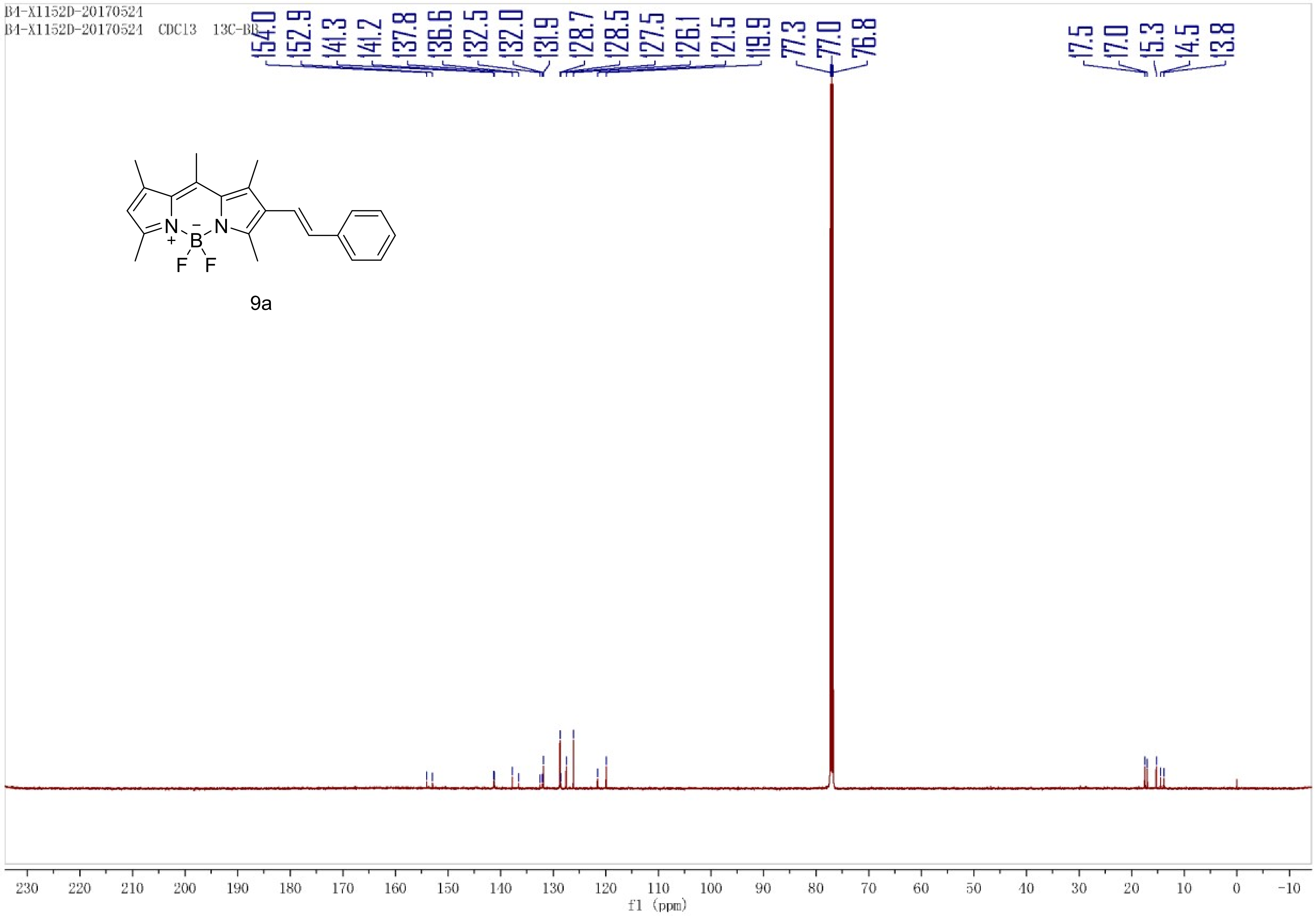
B4-X1152D-20170524
B4-X1152D-20170524 CDCl3 13C-BB
154.0
152.9
141.3
141.2
137.8
136.6
132.5
132.0
131.9
128.7
128.5
127.5
126.1
121.5
119.9
77.3
77.0
76.8
17.5
17.0
15.3
14.5
13.8
N
B
N
F
F
9a
230
220
210
200
190
180
170
160
150
140
130
120
110
100
90
80
70
60
50
40
30
20
10
0
-10
f1 (ppm)

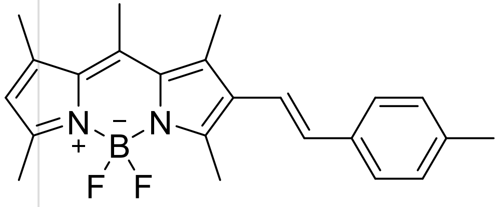

9b

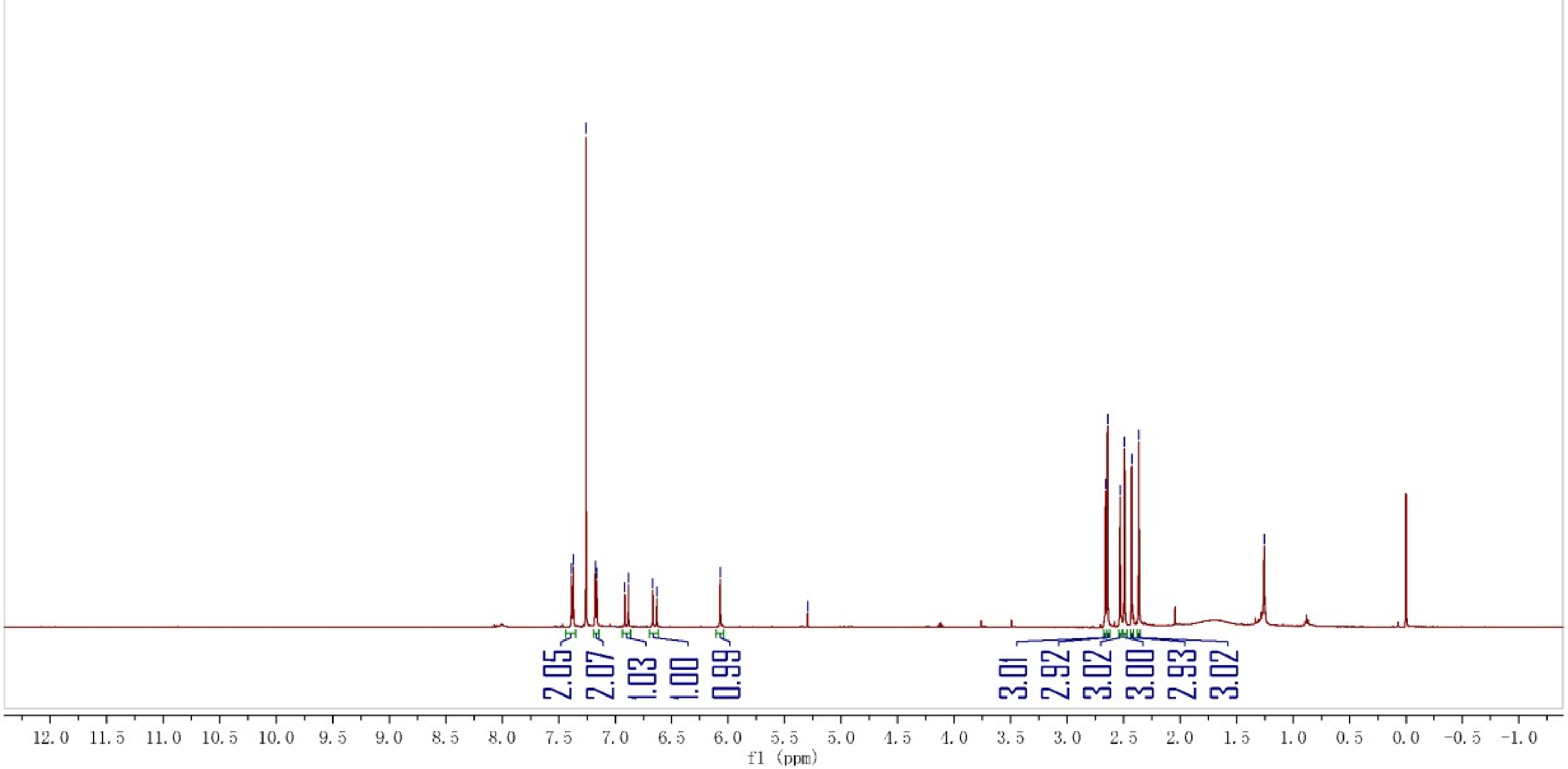

B4-X1152E
B4-X1152E CDCl3 1H
7.39
7.37
7.26
7.18
7.16
6.91
6.88
6.67
6.63
6.07
5.30
2.66
2.64
2.53
2.49
2.43
2.36
1.26
2.05
2.07
1.03
1.00
0.99
3.01
2.92
3.02
3.00
2.93
3.02
12.0 11.5 11.0 10.5 10.0 9.5 9.0 8.5 8.0 7.5 7.0 6.5 6.0 5.5 5.0 4.5 4.0 3.5 3.0 2.5 2.0 1.5 1.0 0.5 0.0 -0.5 -1.0
f1 (ppm)

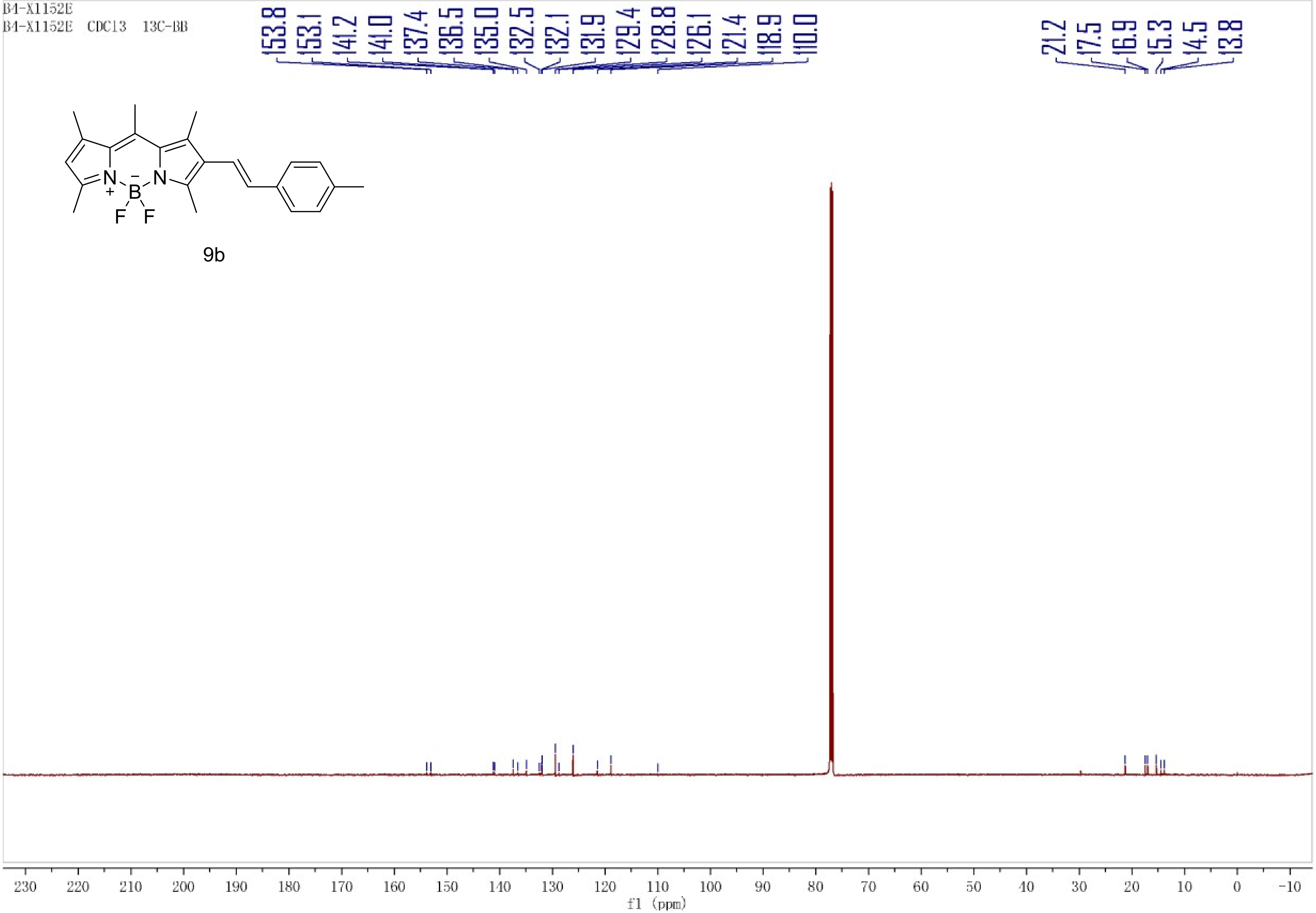
B4-X1152E
B4-X1152E CDCl3 13C-BB
153.8
153.1
141.2
141.0
137.4
136.5
135.0
132.5
132.1
131.9
129.4
128.8
126.1
121.4
118.9
110.0
21.2
17.5
16.9
15.3
14.5
13.8
N
N
B
F
F
9b
230 220 210 200 190 180 170 160 150 140 130 120 110 100 90 80 70 60 50 40 30 20 10 0 -10
f1 (ppm)

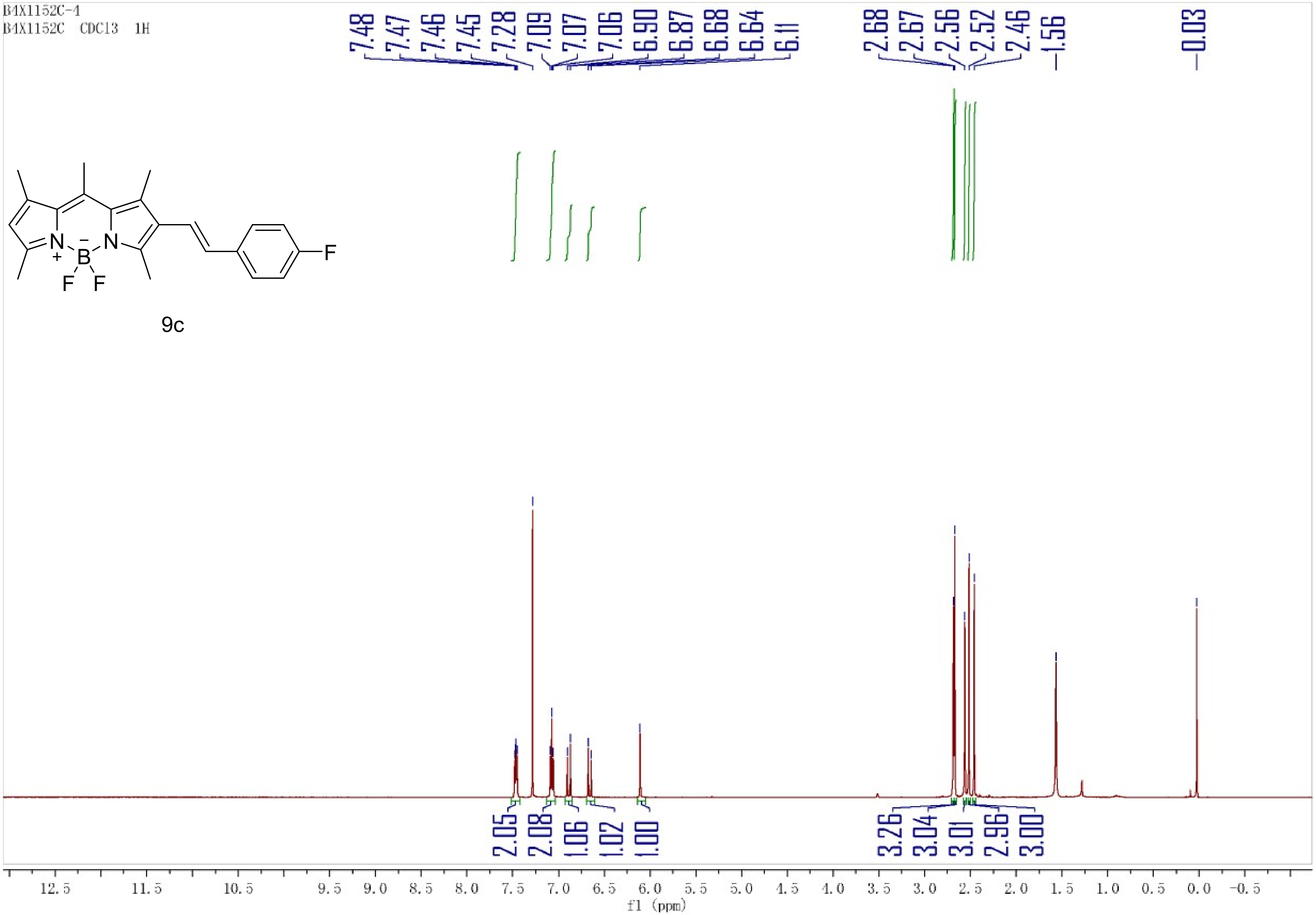

B4X1152C-4
B4X1152C CDCl3 1H
7.48 7.47 7.46 7.45 7.28 7.09 7.07 7.06 6.90 6.87 6.68 6.64 6.11
2.68 2.67 2.56 2.52 2.46 1.56
0.03
2.05 2.08 1.06 1.02 1.00
3.26 3.04 3.01 2.96 3.00
12.5 11.5 10.5 9.5 9.0 8.5 8.0 7.5 7.0 6.5 6.0 5.5 5.0 4.5 4.0 3.5 3.0 2.5 2.0 1.5 1.0 0.5 0.0 -0.5
f1 (ppm)

9c

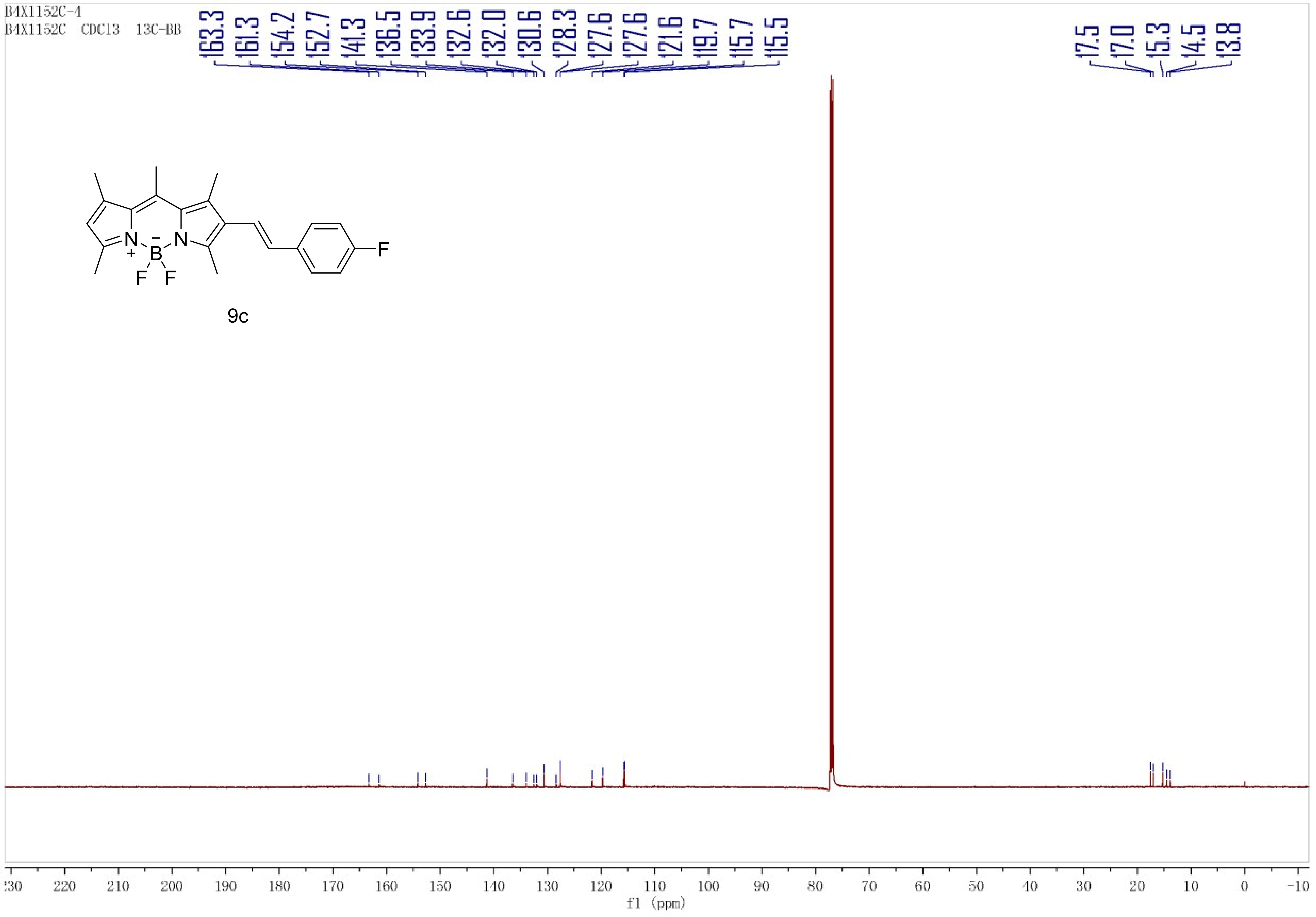
B4X1152C-4
B4X1152C CDCl3 13C-BB
163.3
161.3
154.2
152.7
141.3
136.5
133.9
132.6
132.0
130.6
128.3
127.6
127.6
121.6
119.7
115.7
115.5
17.5
17.0
15.3
14.5
13.8
N
N
B
F
F
F
9c
230
220
210
200
190
180
170
160
150
140
130
120
110
100
90
80
70
60
50
40
30
20
10
0
-10
f1 (ppm)

B4-X1152B5
B4-X1152B CDCl3 1H

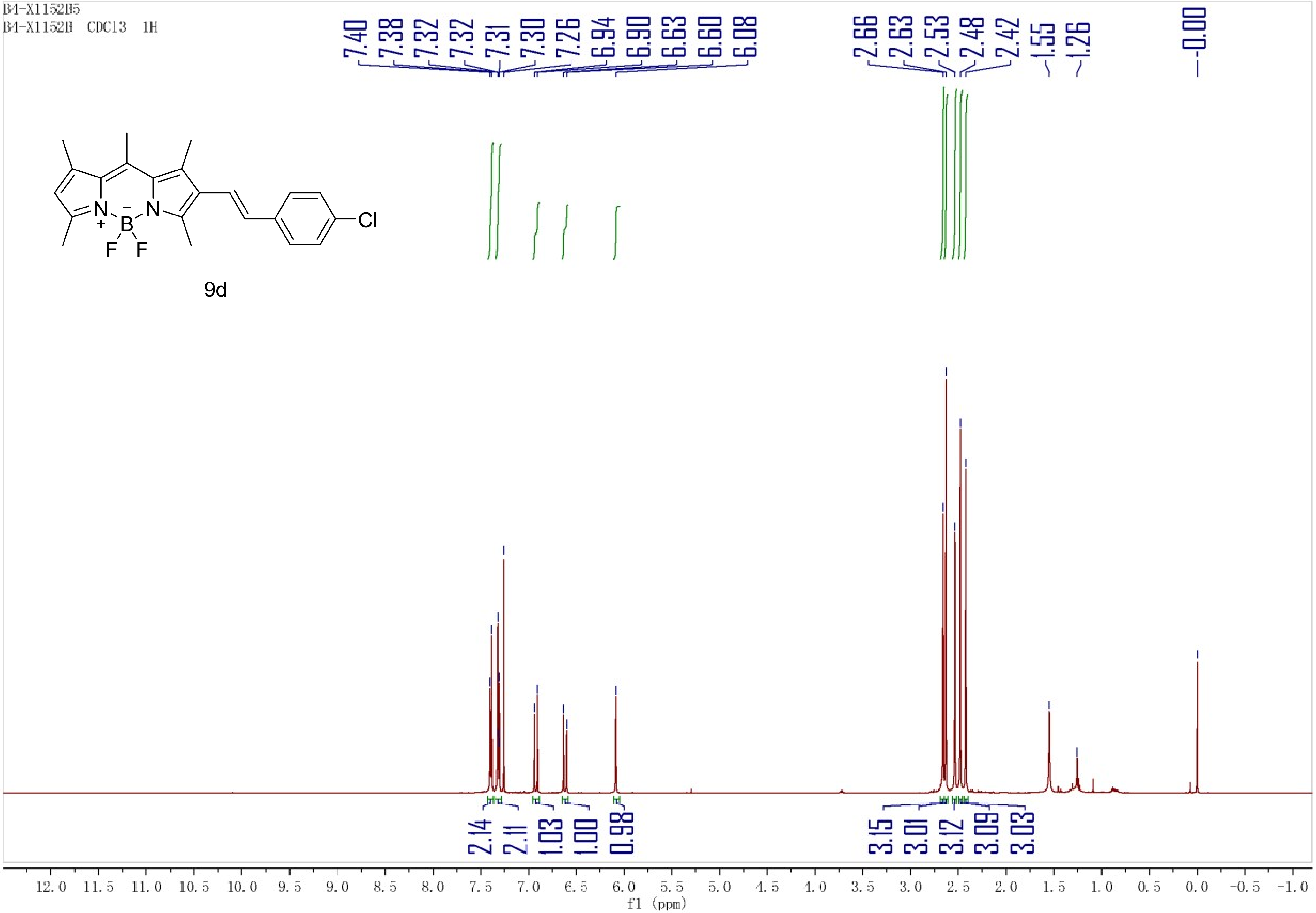

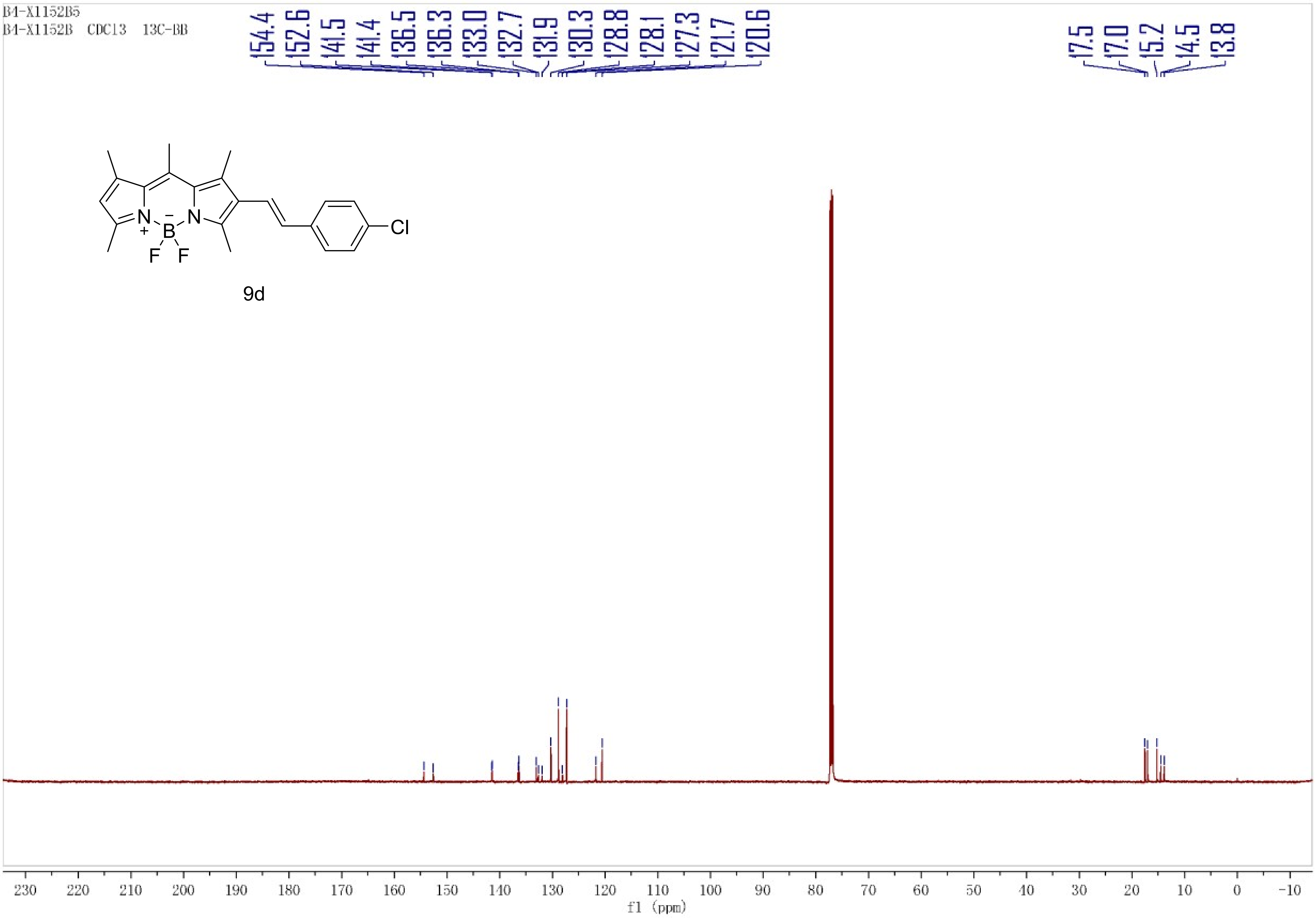
B4-X1152B5
B4-X1152B CDCl3 13C-BB
154.4
152.6
141.5
141.4
136.5
136.3
133.0
132.7
131.9
130.3
128.8
128.1
127.3
121.7
120.6
17.5
17.0
15.2
14.5
13.8
N
B
N
F
F
Cl
9d
230 220 210 200 190 180 170 160 150 140 130 120 110 100 90 80 70 60 50 40 30 20 10 0 -10
f1 (ppm)

b4-x1152a
0045-19 CDCl3 1H

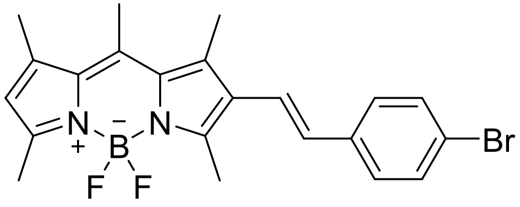

9e

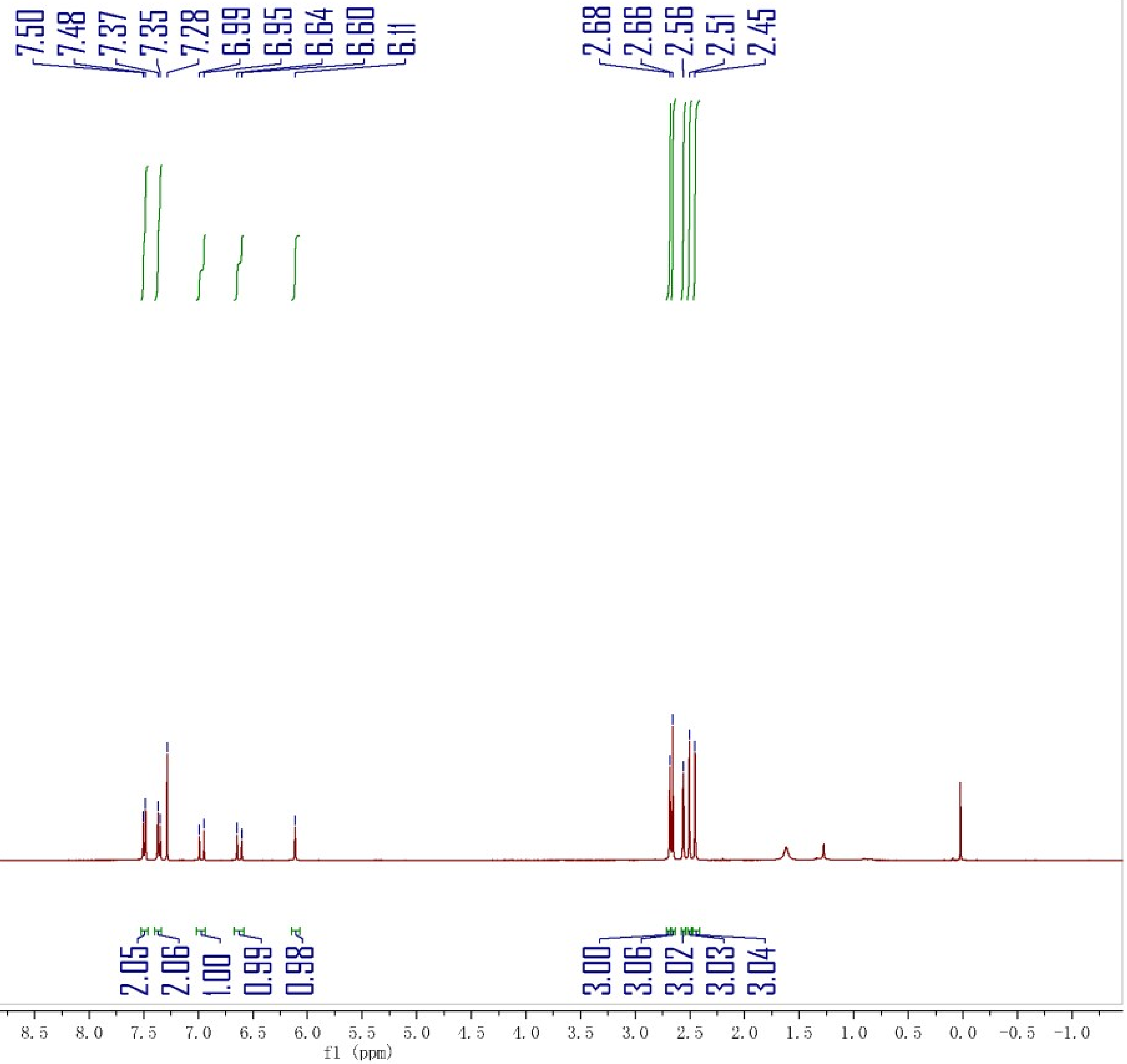

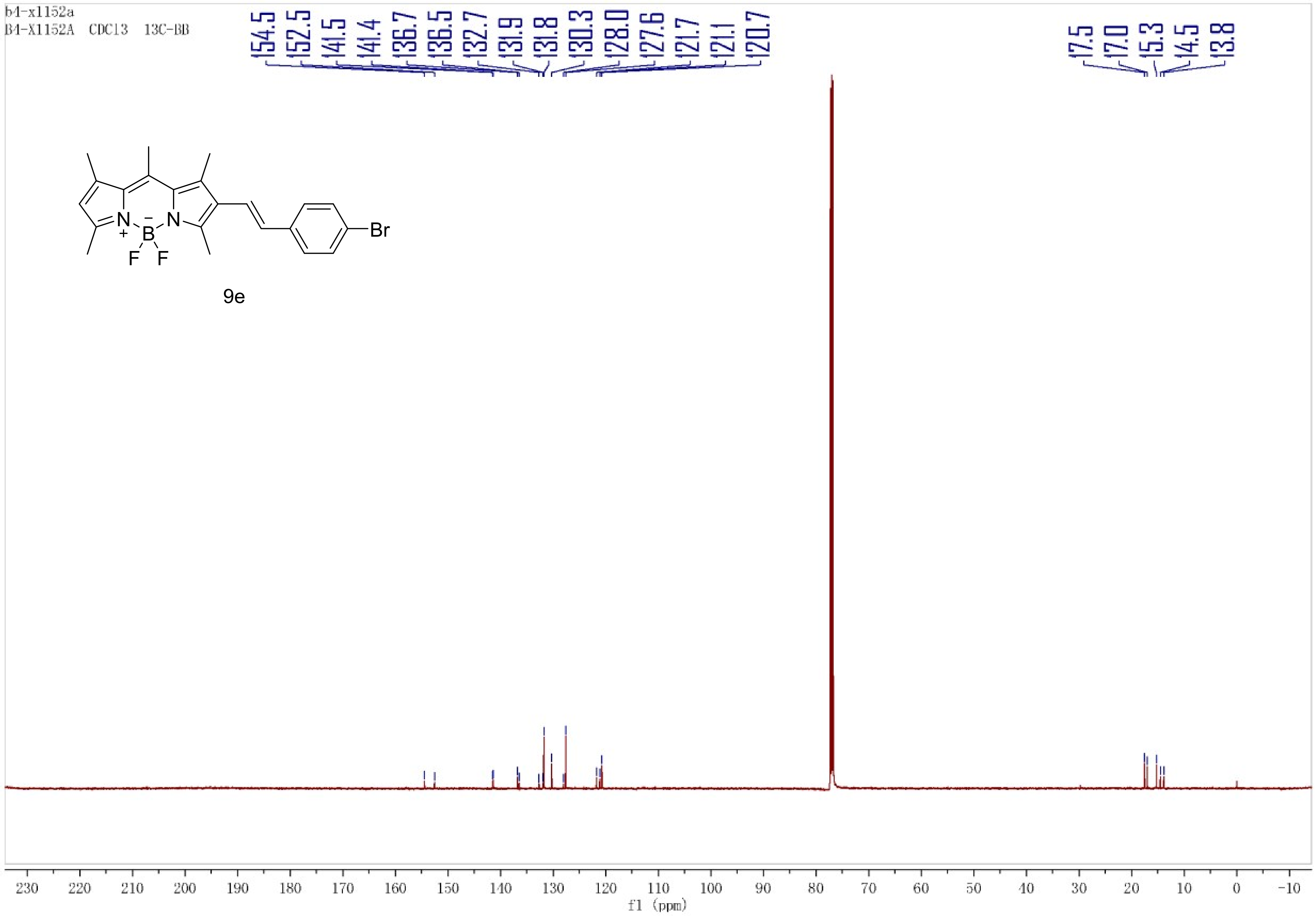

b4-x1152a
B4-X1152A CDCl3 13C-BB
154.5
152.5
141.5
141.4
136.7
136.5
132.7
131.9
131.8
130.3
128.0
127.6
121.7
121.1
120.7
17.5
17.0
15.3
14.5
13.8
230 220 210 200 190 180 170 160 150 140 130 120 110 100 90 80 70 60 50 40 30 20 10 0 -10
f1 (ppm)

9e

B4-X1152F
B4-X1152F CDCl3 1H

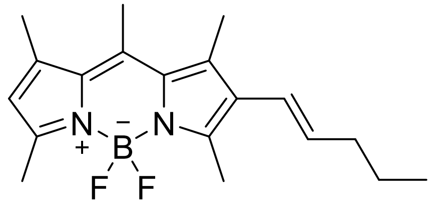

9f

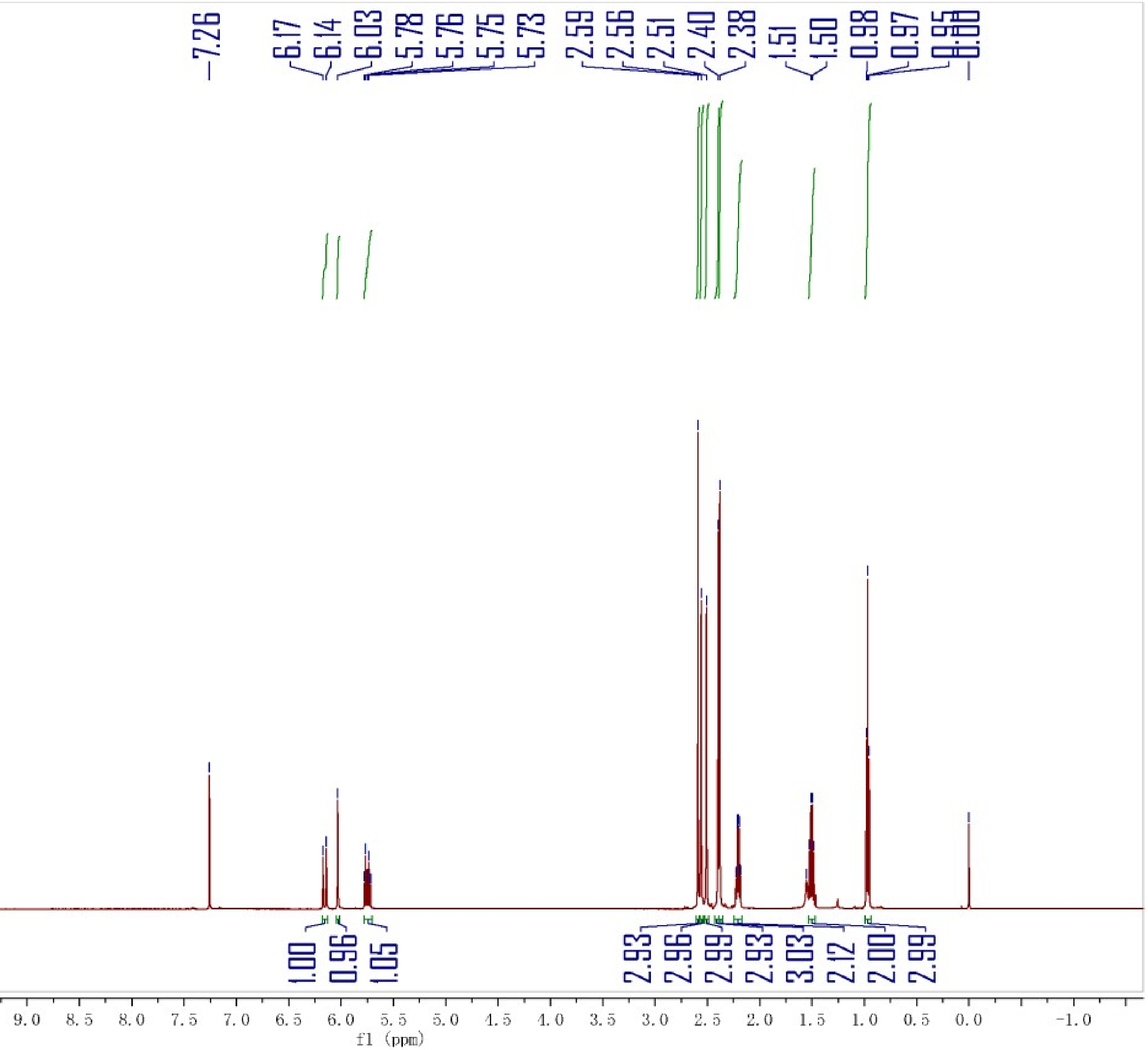

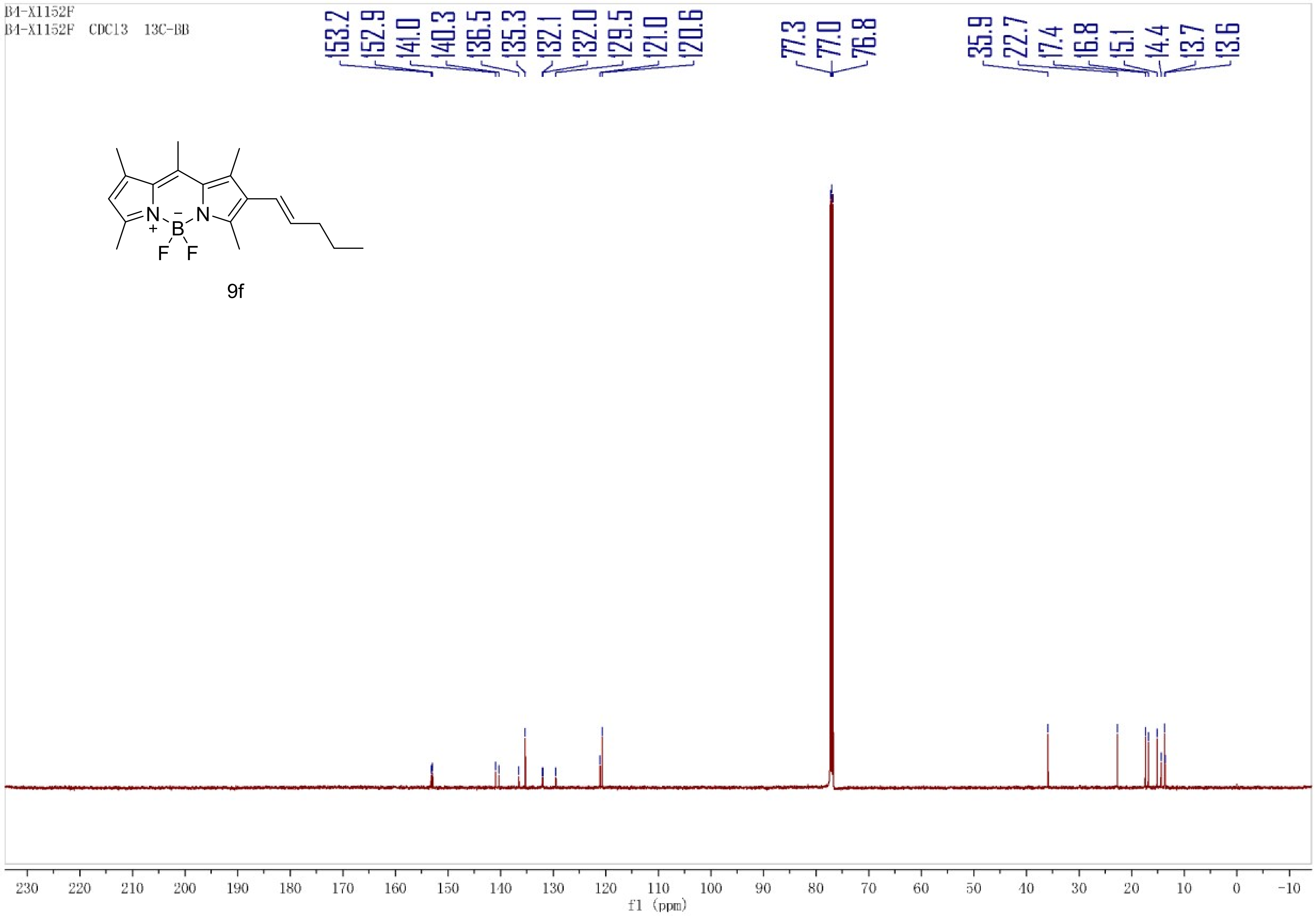
B4-X1152F
B4-X1152F CDC13 13C-BB
153.2
152.9
141.0
140.3
136.5
135.3
132.1
132.0
129.5
121.0
120.6
77.3
77.0
76.8
35.9
22.7
17.4
16.8
15.1
14.4
13.7
13.6
N
B
N
F
F
+
−
9f
230 220 210 200 190 180 170 160 150 140 130 120 110 100 90 80 70 60 50 40 30 20 10 0 -10
f1 (ppm)
